# AI-Based Retrosynthesis of Known and Unexplored Covalent Organic Framework Monomers

**DOI:** 10.1021/acs.chemmater.6c00008

**Published:** 2026-07-29

**Authors:** Chin-Fei Chang, Srinivas Rangarajan

**Affiliations:** Department of Chemical and Biomolecular Engineering, 1687 Lehigh University, 124 E Morton Street, Bethlehem Pennsylvania 18015, United States

## Abstract

Covalent organic frameworks (COFs), owing to their structural tunability, have a massive design space, of which only a fraction (∼1,300) has been experimentally reported. Using AI-based retrosynthetic tools for the first time in this context, we here address one of the inherent challenges in realizing hypothetical COFs, viz. the synthesis of identified monomers (building blocks). Two general retrosynthetic tools, ASCKOS and AiZynthFinder, showed a combined success rate of 76% in identifying routes to 359 experimentally reported monomers (of 600 2D COFs) with uniform performance across structural motifs. Further, these tools successfully identified synthesis routes to 83 unexplored monomers, capable of forming at least 1,200 hypothetical COFs. This work underlines the efficacy of modern retrosynthesis tools in addressing a challenge in the designed synthesis of COFs.

## Graphical abstract

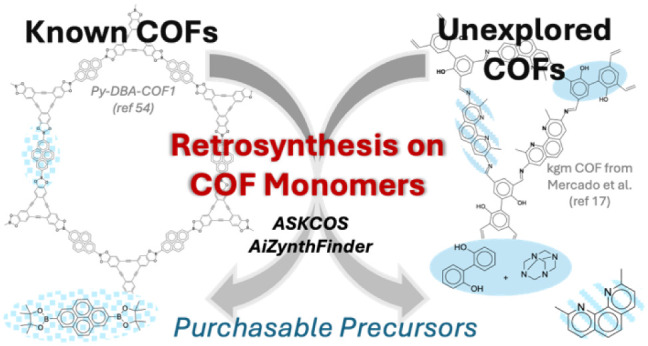

## Introduction

Covalent organic frameworks (COFs) form a subclass of crystalline reticular materials with a well-defined pore structure,^1^ which enables their application in catalysis,^2,3^ energy storage,^4–7^ drug delivery,^8,9^ etc. COFs possess a high degree of structural tunability, making them desirable as “designer” materials for targeted properties. The reticular synthesis of COFs has been systematically reviewed, and the building block monomers have been categorized in multiple works based on symmetry, chemical functionality, or both;^10–14^ this has resulted in guidance for the topological design of COFs, such as (1) hexagonal pore COFs can arise from combinations of tritopic (C_3_) and ditopic (C_2_) monomers or via a combination of two C_3_ molecules. (2) a triangular lattice can be derived from C_2_ and C_6_ monomers, etc. Computational studies have further leveraged these rules to automatically construct hypothetical COFs. Extensive libraries of 2D and 3D COFs have been proposed, including the ReDD-COFFEE database comprising >260,000 COFs assembled from 275 secondary building units,^15^ a database of >470,000 COFs using material genomics techniques with 130 generic structural units,^16^ and ∼70,000 COFs through virtual structural assembly from hundreds of plausible monomers^17,18^ many of which are yet to be synthesized.

Despite the vast space of hypothetical COFs, the number of known (synthesized) frameworks is still under 1,300,^19,20^ albeit steadily growing. This is due to two broad synthesis challenges: (1) identifying the right building blocks (monomers) and synthesizing them at high purity, and (2) reticulating them optimally to maximize the crystallinity of the resulting framework.^21–25^ While obtaining a crystalline COF is often considered the more difficult step in practice, the selection and synthesis of suitable monomers remain critical prerequisites for successful framework construction. Here, we address the challenge of evaluating the synthetic feasibility of identified monomers. When a monomer is not readily available off-the-shelf, retrosynthesis^26^ can be employed to assess its synthetic feasibility and assist in synthesis planning. Recent advances in artificial intelligence (AI) algorithms have enabled the development of modern retrosynthetic software that either trains neural networks to select proper reaction templates to deconstruct an unknown molecule or employs transformers to guide backward synthesis template-free.^27–30^ More recently, studies have focused on enhancing retrosynthesis performance by leveraging the chemical reasoning capability of large language models (LLMs), aiming to enable strategic synthesis planning that aligns more closely with expert chemical intuition and the availability of synthetic resources.^31^

Emerging AI-based retrosynthetic tools have shown remarkable performance in general organic chemistry; however, they have not been systematically applied in the context of COF-building-block monomers (Figure 1). To this end, we first use two state-of-the-art retrosynthesis platforms, ASKCOS^32–34^ and AiZynthFinder,^35,36^ to predict the synthetic routes for the experimentally validated 2D COF monomers and evaluate their efficacy by analyzing the resulting solutions based on structural motifs identified through molecular clustering. Once we establish the baseline performance, we then apply these tools to identify the synthesis routes to the monomers of hypothetical COFs, thereby providing an initial basis for experimental access to them.

**Figure 1:**
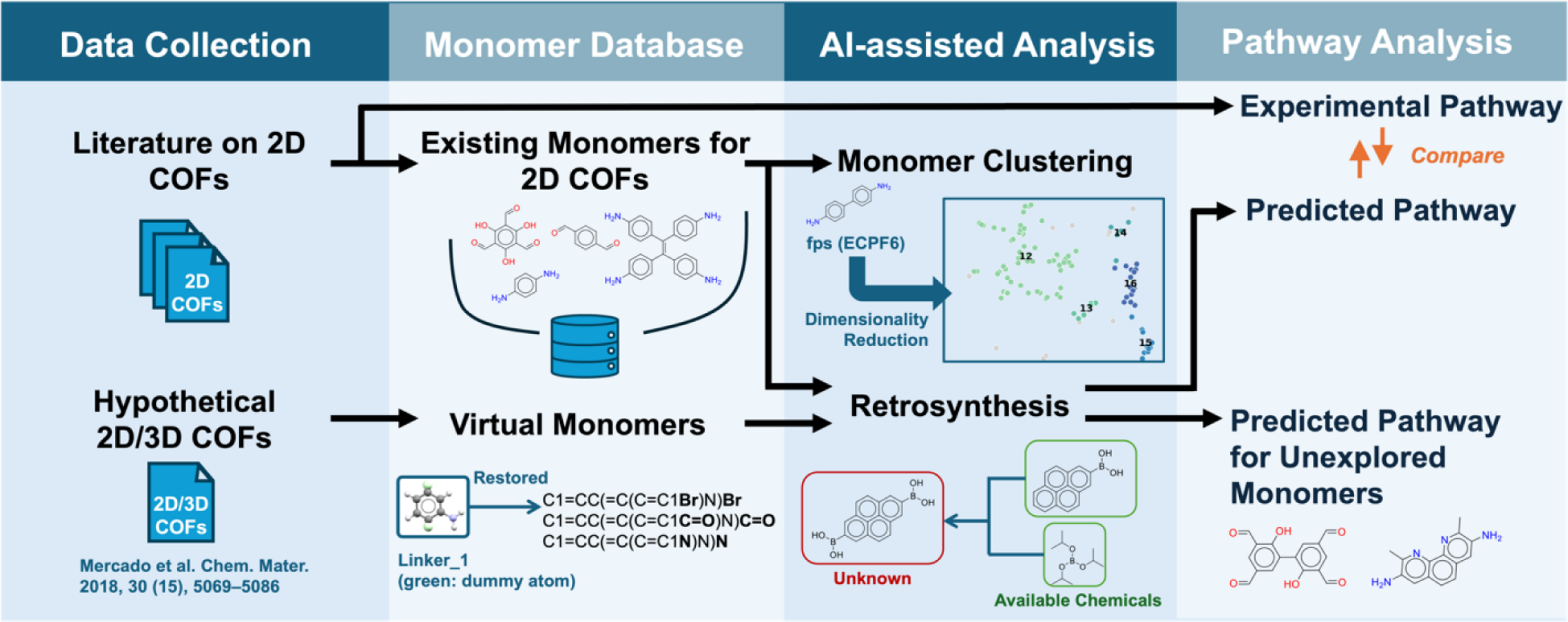
A workflow for 2D COFs monomer curation, structural clustering analysis, and comparison of synthesis pathways predicted by AI retrosynthesis tools with experimentally reported pathways.

## Methods

### COF Monomer Data Extraction

Five hundred and eighty-three (583) 2D COFs were collected from a well-curated public data set^37^ (accessed in March 2023), which, in turn, were extracted from two hundred and ninety-six (296) published papers. In the current study, we only considered monomers used directly in the synthesis of COF; studies focused on postsynthetic modifications were excluded. The SMILES strings of the monomers were extracted using MolScribe,^38^ a chemical image recognition tool, which had an ∼53% success rate; the remaining cases required manual intervention. In total, we extracted 843 SMILES strings from 282 publications. After removing the duplicates and entries unreadable by RDKit,^39^ 359 unique monomers remained (listed in Table S3), including vertex molecules and linkers. For scaffold identification, we utilized the built-in Murcko decomposition from RDKit. Details of the 2D COFs literature and JSON files translated by MolScribe can be referred to Section S2 of Supporting Information (SI).

### COF Monomers from 2D/3D Hypothetical COF Database

The hypothetical database of 69 840 COFs proposed by Mercado et al.^18^ was selected for analysis of their synthesizability down to the level of building block monomers. All 666 monomers used in the formation of hypothetical COF are presented in “connected” mode, where the connecting point is represented by a dummy atom. To determine whether these monomers are synthesizable, we restored the dummy atoms to explicit functional groups (bromide, aldehyde, amine) using RDKit SMARTS reaction templates. While the authors report 666 distinct monomers with four distinct covalent bonding types, the number of restored monomers is only 333 because −C=O/–CH/–CH2 terminations are all reverted to aldehyde (−C=O), and −NH/–N terminations are reverted to amine (−NH_2_); carbon–carbon bonds are represented using −Br terminations, as indicated in Tables 1 to 9 of the Supporting Information in the publication by Mercado et al.^18^ The restored monomers were then examined, and those not found in PubChem were identified for subsequent retrosynthetic analysis to obtain potential synthetic pathways.

### Monomer Clustering Analysis

To perform monomer clustering, we used RDKit to generate ECFP6 molecular fingerprints, with a radius set to 3, considering the fact that most monomers contain aromatic rings and have large scaffolds. To capture both local and global structural features, we applied UMAP,^40^ a dimensionality reduction technique, on the molecular fingerprints, followed by HDBSCAN^41^ for clustering the resulting 2D projection. We conducted a parameter ramp test on UMAP (Figure S8 in Section S3.1) and selected the configuration with values for the number of neighbors (n_neighbors = 15) and minimum distance (min_dist = 0.001). The selected parameters produced the fewest noise points (22 monomers) and yielded clusters with intracluster Dice similarity^42^ scores greater than 0.3, excluding the noise cluster (Tables S1 and S2 in Section S3.1). Once the clusters were defined, we computed the centroid molecules of each cluster to identify the dominant molecular features. The centroid was defined as the molecule with the highest cumulative similarity to all of the other monomers within its cluster. Finally, we applied the GPT-4o model^43^ as a supplementary tool to assist in interpreting the dominant scaffolds and key structural fragments within each cluster by providing a list of SMILES for each cluster. This analysis was used to support qualitative interpretation, and additional details of the procedure are provided in Section S4.

### Retrosynthesis Analysis

We applied two template-based retrosynthesis tools, ASKCOS^32–34,44^ and AiZynthFinder,^35,36^ to search for synthesis routes to both existing and unreported monomers. We picked these tools for their ability to generate complete multistep synthetic routes using search algorithms like Monte Carlo Tree Search (MCTS)^45^ and Retro*,^46^ rather than limiting the prediction to single-step transformations.

AiZynthFinder (accessed May 2023) is trained on the USPTO reaction dataset and uses ZINC as its stock resource. In contrast, ASKCOS (version 2, accessed July 2024) is trained on Reaxys reaction templates and uses Sigma–Aldrich and eMolecules as its stock libraries. For AiZynthFinder, we selected MCTS as a searching algorithm and specified a maximum of 6 transformation steps, a 60-s search time limit, and 500 iterations. For ASKCOS, we deployed a minimal configuration, including only the template-relevance model, in order to accommodate hardware constraints. We applied the Retro* algorithm with the same limits on search depth, search time, and iterations as those used in AiZynthFinder. These settings were chosen to align the tree-search portion of route construction across the two tools. Other tool-specific settings related to one-step expansion, that is, the generation and ranking of precursor suggestions at each search step, were not further tuned and were kept at their default configurations within each tool. Additional configuration details are provided in the scripts included in the Supporting Information. While both tools support the integration of custom reaction templates and stock molecules, we used only the default models and libraries to ensure a consistent comparison.

Although in the latest versions (August 2025), both tools support MCTS and Retro* as search algorithms, we observed that the MCTS-based AiZynthFinder achieved a higher solved ratio (47%) compared to its Retro* implementation (43%). Conversely, ASKCOS with Retro* yielded a significantly higher solved ratio (70%) than ASKCOS with MCTS (46%). Therefore, by combining the predicted routes from the MCTS-based AiZynthFinder and the Retro*-based ASKCOS, we maximized the overall number of successfully solved monomers.

## Results and Discussion

We begin with a data-driven analysis of monomers of known COFs and subsequently discuss the successes and challenges of retrosynthetic tools in identifying synthetic routes to these molecules. Finally, we present synthesis routes for unknown monomers of hypothetical COFs.

### Monomers of Known COFs

An analysis of monomer usage showed that the top 20 most frequently applied ones account for 42% of all curated instances (356 out of 843), as shown in Figure 2, which suggests a strong bias toward a limited structural space in the current COF design. Notably, the 2,4,6-triformylphloroglucinol (or 2,4,6-trihydroxybenzene-1,3,5-tricarbaldehyde) emerged as the most frequently used monomer (molecule labeled mol-0 in the right panel of Figure 2), appearing in 40 publications. Its high symmetry and planarity make it ideal for forming hexagonal COFs, as the aldehyde functional groups are separated by exactly 120°.

**Figure 2:**
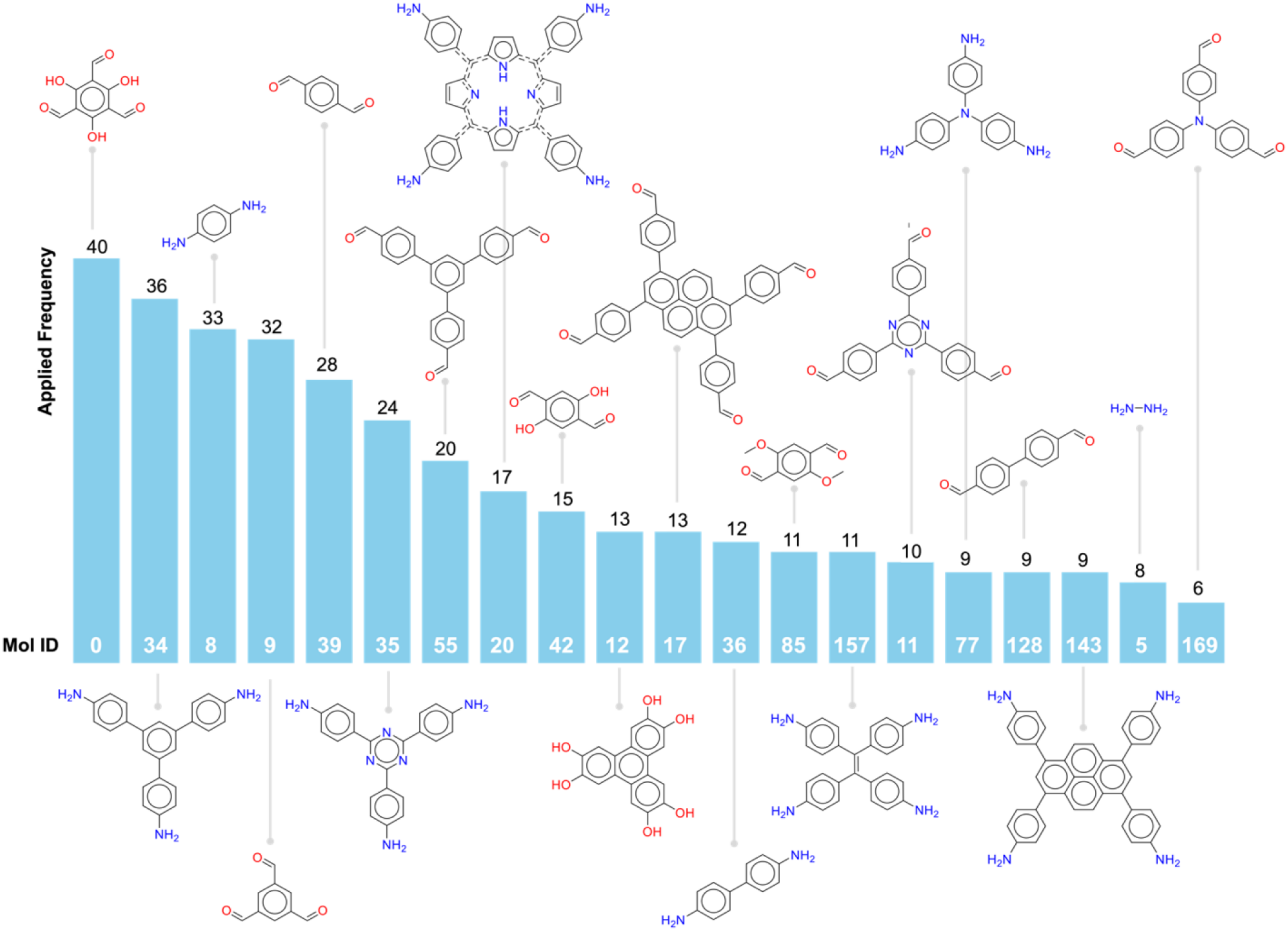
The Top 20 most frequently applied monomers for COF synthesis. The left bar chart shows the applied frequency (count of literature) for the top 20 monomers (molecules displayed on right).

To better understand the core structures of these frequently used monomers, we extracted their molecular scaffolds using RDKit (as described in Methods section). All monomers contain at least one aromatic ring, except for hydrazine (as listed in Figure S1) which is the shortest linear linker used in COFs literature. Unsurprisingly, benzene is the most dominant scaffold, appearing six times among the top 20 monomers. The remaining scaffolds reflect a diversity of structural connectivity, including 120° (triangular), 90° (square), and linear 180° geometries, which are also critical in directing the formation of COF topology.

Previously, the COF monomers have been grouped based on their functional groups, symmetry, or a combination of both.^11–13^ While these simple categorizations provide practical information when selecting molecules for COF assembly, here, a more detailed clustering analysis was carried out on existing 2D COF monomers based on molecular fingerprints to elicit structural and connectivity trends in COF monomers. Therefore, the existing 2D COF monomers were clustered based on the molecular fingerprints to elicit structural and connectivity trends in the COF monomers. We reiterate that monomers can either be vertices or linkers; typically, the vertex monomer determines the final topology, while the linker connects two vertex molecules and enables controlling pore sizes.^47^ Here, vertex molecules and linkers are considered together because they both determine the final COF structure, and some COFs are synthesized without linkers (BABN-DP COF^48^ and NiPc-NH-CoPcFs^49^), while a subgroup of COFs is synthesized solely with linkers (COF-1,^50^ PPy-COF,^51^ BLP-2­(H),^52^ and CTF-1 ^53^).

The cluster analysis using UMAP and HDBSCAN (Figure 3) results in 17 distinct clusters, with 22 monomers labeled as noise (cluster labeled as −1). Each group is fairly well-represented by the centroid molecule (center of Figure 3, also Section S3.2 and Figures S9–S26 of Supporting Information), except for cluster 14, where some of the constituting molecules could be classified into other structures. The Dice similarity score of each cluster is at least 0.3, indicating shared substructures within the group. The spatial proximity between each cluster reflects the structural similarity according to molecular fingerprints. Generally, we observed that the clusters are relatively well-separated, with some dense regions indicating a group of highly structurally similar monomer classes was present. Clusters 12–14 and 16 are somewhat densely clustered at the center; this is intuitive given that all of them are made up of aromatic amines (as vertices or linkers). In the same vein, (i) clusters 5 and 15 are oxygen-functionalized linkers and vertices, respectively, (ii) 8 and 9 are both boronic acids, (iii) 4, 6, and 7 are all aromatic aldehydes, and (iv) 10 and 11 both have hydrazide ends. On the other hand, clusters 0–3 are relatively isolated from the center, given their atypical motifs (e.g., cluster 0 comprises porphines, 1 has C–C triple bonds, which are rare in COFs, 2 has benzimidazoles, and 3 has nitrile groups). Aside from the manual interpretation of the key structural fragments in each cluster, we explored the use of the GPT-4o model^43^ as a supplementary tool for qualitative scaffold interpretation; the detailed procedure and result discussion can be referred to Section S4.

**Figure 3:**
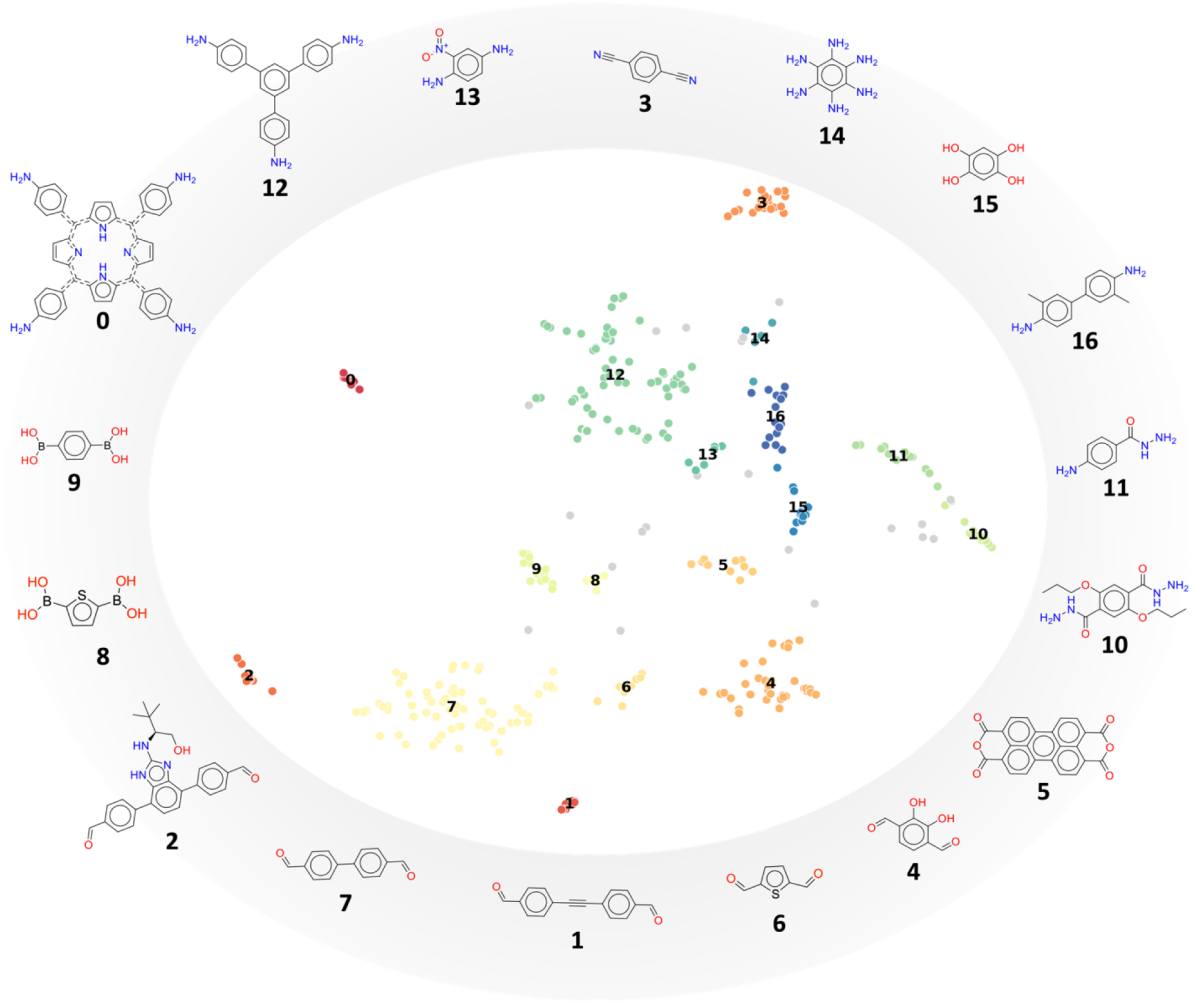
The Clustering of existing 359 monomers using UMAP and HDBSCAN is shown in the center; Each dot in the 2D plot represents one monomer. In total, 17 distinct clusters are identified, along with an additional noise cluster (gray dots). Molecules shown in the outer circle are the centroid molecules of each cluster.

### Retrosynthesis Analysis on Existing Monomers

Next, we examined the possible synthetic routes of these monomers using the ASKCOS and AiZynthFinder. These monomers are either available off-the-shelf or have been synthesized by the authors of these publications, thus providing a rigorous basis for evaluating these tools in the context of COFs. In the following sections, we selected one monomer for each case to demonstrate the success of these retrosynthesis tools and their challenges. While no expert validation was performed for every predicted retrosynthetic route, literature-reported syntheses were used as external checks, where available. More examples can be found in Figures S27 and S28 in Section S5.1 of the Supporting Information.

### Case I: Solved Monomer

The synthetic route of pyrene-2,7-diboronic acid (PDBA, mol ID 24) is successfully predicted by both tools. PDBA has been used in the synthesis of **TP-COF**,^54^ **PPy-COF**,^51^ **Py-DBA-COF 1**,**Py-DBA-COF 2**, and **Py-MV-DBA-COF**,^55^ as well as in a series of hexagonal and tetragonal **MC–COFs** reported by Huang et al.^47^ In the synthesis of TP-COF, Wan et al. reported the synthetic steps for PDBA using precursor **24a**.^54^ The transformation from boronic esters to boronic acids was achieved via oxidative hydrolysis using sodium periodate (NaIO_4_), a method originally described by Tzschucke and coworkers.^56^

The top-ranked predicted synthetic route from ASKCOS (Figure 4a) is identical with the synthesis reported in the literature. In contrast, the pathway predicted by AiZynthFinder suggests using **24b** and **24d** as precursors, followed by the oxidation of trimethyl borate to form the boronic acid (bottom pathway in Figure 4a). Another pathway, involving **24b** and **24c** as precursors, also appears among the solved solutions in both tools (middle pathway in Figure 4a).

**Figure 4:**
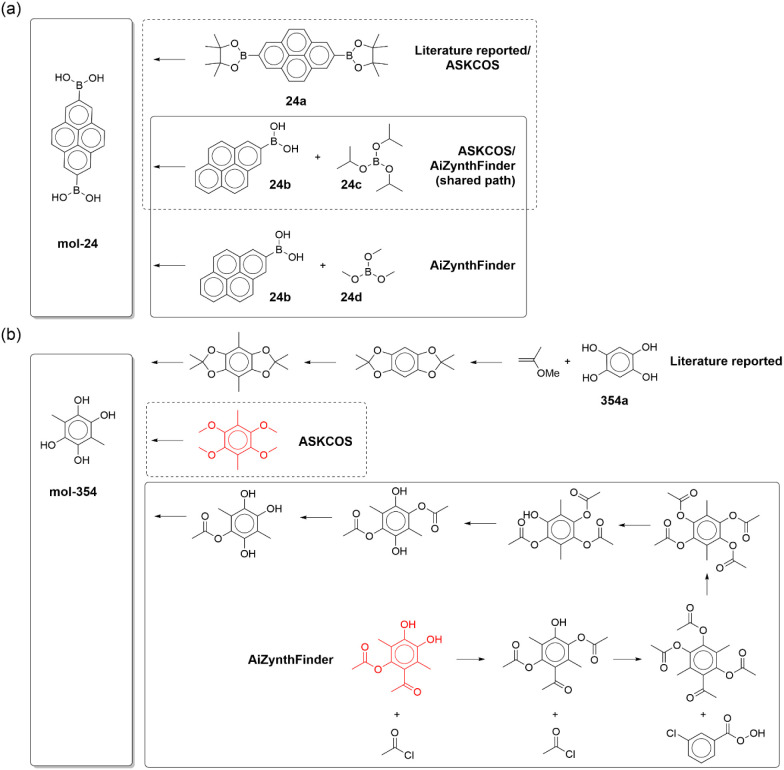
(a) Solved synthetic route predictions for Pyrene-2,7-diboronic acid (PDBA, mol ID 24) by ASKCOS and AiZynthFinder, are compared with the literature-reported route. (b) Unsolved synthetic route predictions for 3,6-dimethyl-1,2,4,5-tetrahydroxybenzene (mol ID 354) by ASKCOS and AiZynthFinder, are compared with the literature-reported route. Routes predicted by ASKCOS are circled with dashed lines, and those by AiZynthFinder are circled by solid line. Molecules highlighted in red represent starting molecules not available in stock libraries.

### Case II: Unsolved Monomer

On the other hand, both retrosynthesis tools failed to find a synthetic route to 3,6-dimethyl-1,2,4,5-tetrahydroxybenzene (mol ID 354), a linker used in the synthesis of COF-16A.^57^ As shown in Figure 4b, Tilford et al. reported the synthesis of this monomer starting from the commercially available 1,2,4,5-tetrahydroxybenzene (**354a**). The route involves a protection–deprotection strategy, wherein (a) the two pairs of hydroxyl groups are first protected by forming dioxole rings via acetalization, (b) the benzene rings are alkylated at the 3- and 6-positions of the benzene ring, and (c) the dioxole rings are cleaved to regenerate diols and yield the desired product.

ASKCOS was ultimately unsuccessful, however, it correctly recognized the need to protect the diol groups (via etherification). The prediction subsequently failed, potentially due to the unavailability of an appropriate reaction template for dealkylating the methyl-protected intermediates. AiZynthFinder also attempted to protect the hydroxyl groups, however, subsequently, it was repeatedly transforming the hydroxyls into derivatives such as ethers or methyl acetates and failing to progress.

### Synthesizability Across Clusters

Overall, the two retrosynthesis tools successfully predicted synthetic routes for 272 monomers under the default settings (methods section) and with the built-in molecular stock libraries. Among the remaining cases, 87 monomers remained unsolved, including 9 that had no predicted route but were already present in the stock libraries. For the successfully predicted cases, 150 monomers were identified as solvable by both tools, whereas 102 were uniquely solvable by ASKCOS and 20 solely by AiZynthFinder, as shown in Figure 5a. Overall, the success rates of both retrosynthesis tools are comparable to the results reported by Genheden et al.^35^ using AiZynthFinder (55% solved) and ASKCOS (62% solved) for predicting 100 molecules from ChEMBL.

**Figure 5:**
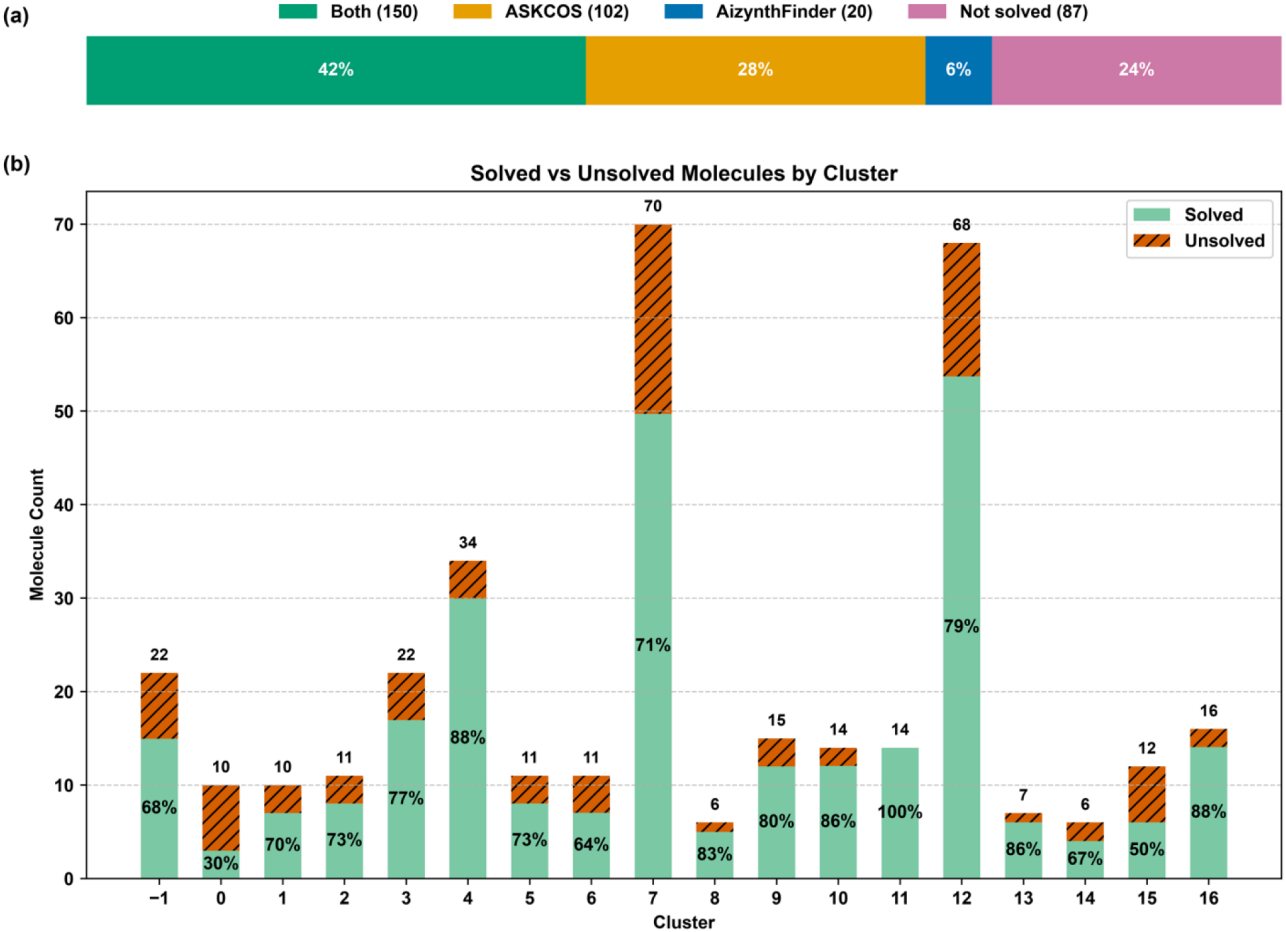
Retrosynthesis prediction success rate for 359 COF monomers. (a) Distribution of retrosynthesis results across all monomers. Predictions are categorized into four groups: solved by both ASKCOS and AiZynthFinder (green), uniquely solved by ASKCOS (orange), uniquely solved by AiZynthFinder (blue), and unsolved by either tool (pink). (b) Cluster-wise breakdown of solved (green) and unsolved (orange) monomers.

These tools have consistent predictive power across most clusters (see Figure 5b) with a uniform success rate of 70%, with the exception of four of them. Of these four clusters, cluster 0, dominated by a porphine backbone, stands out as the most challenging, with a solved ratio of only 30%. Porphines are ideal for square or T-shaped geometries (with vertex angles of 180°–90°–90°), however, their complex fused-ring structure increases their synthetic complexity beyond the reach of these tools.

One of the unsolved monomers of cluster 0, viz., tetra­(p-boronic acid-phenyl)­porphyrin (TBPP), was used in the synthesis of COF-66,^58^ , which was reported as the first COF incorporating porphyrin-based monomers with C_4_ symmetry. Wan et al.^51^ reported the synthesis of TBPP from pyrrole and 4-formylphenylboronic acid pinacol ester, which formed the porphyrin ring. In the same study, the authors also synthesized tetra­(*p*-amino-phenyl)­porphyrin (TAPP) for the construction of COF-366. In our analysis, TAPP has a predicted synthetic route from ASKCOS, but not from AiZynthFinder (Figure S2). The predicted route utilizes commercially available porphine-containing intermediates, thereby avoiding the need to construct the complex porphine macrocycle from scratch. In contrast to the predicted route, the authors in the original study synthesized TAPP following a published method^59^ in which pyrrole and p-nitrobenzaldehyde were used as precursors (similar to TBPP), with acetic anhydride as a dehydrating agent and propionic acid as a catalyst to assemble the porphine ring. A similar difficulty in retrosynthetic prediction is observed in cluster 15, which contains monomers composed of multiple benzene rings connected by alkynes.

In clusters with higher prediction success rates (>85%), including clusters 4, 10, 11, 13, and 16, monomers comprised simpler aromatic scaffolds such as benzene, biphenyl, or naphthalene, making them more solvable by retrosynthesis tools. These monomers also tend to carry common functional groups such as aldehydes, hydroxyl groups, and amines, which are well represented in commercial stock libraries and common reaction templates.

The synthetic accessibility, i.e., SAscore,^60^ is also calculated for all 359 monomers, which shows a range from 1 (easiest) to 7 (most difficult) in Figure S3. Monomers solvable by both ASKCOS and AiZynthFinder (flag = 2) generally have a lower average score (2.4), whereas those solvable by only one tool (flag = 1) average 2.7. In contrast, monomers without any successful predicted route (flag = 0) have an average score of 3.7. This trend is comparable to the solvable/unsolvable distribution of 100 ChEMBL compounds reported by Genheden et al.,^35^ indicating the potential for further improvement of these tools through larger collections of reaction templates and stock libraries. Prior work has also shown that dataset composition and stock availability can influence the number of routes identified by computer-assisted synthesis planning tools.^61^ Because ASKCOS and AiZynthFinder employ different default stock libraries and reaction template sets, the differences in solved ratios observed here should be interpreted cautiously and not as a direct comparison of the intrinsic qualities of the two algorithms. Nevertheless, both tools employ large default stock libraries of commercially available compounds, and the probability of missing well-known synthetic routes is likely low. Thus, while retrosynthesis tools are intuitively more successful with relatively simpler monomers, the generally high success rate of >70% indicates their ability to identify synthesis routes to unknown monomers of hypothetical COFs.

### Retrosynthesis Analysis of Unexplored Monomers

Next, we considered the synthetic feasibility of monomers employed by Mercado et al.^18^ Three hundred and thirty-three (333) distinct monomers were identified (although authors suggest 666 virtual monomers, only 333 are distinct, others are permutations or derivatives of these). Among the 333, 224 are documented in at least one of PubChem,^62^ ChemSpider,^63^ or eMolecules^64^ and are thus considered known. Retrosynthetic analysis was applied to the remaining 109 fictitious monomers. With the same search settings described in Methods section, 83 out of the 109 unknown monomers (Figures S4–S6) have at least one successful predicted synthetic route from ASKCOS (80 solved) or AiZynthFinder (50 solved); the remaining 26 monomers (Figure S7) could not be resolved using the default in-stock molecules and reaction templates. The 224 known and the 83 unknown (but solvable) monomers together cover about 81% (56,908) of the hypothetical COFs proposed in that study. All the 333 monomers, along with their IUPAC names extracted from PubChem (if applicable), are listed in Tables S4–S6. The potential synthetic routes of the 83 solved monomers can be retrieved from the Supporting Information (Section S5).

### Synthesizable Unexplored COF

The 83 unknown monomers can result in 1,267 hypothetical COFs spanning 39 distinct 3D nets and one 2D net (**kg m**). We selected one representative **kg m** COF (from a total of 45 plausible ones); this COF (Figure 6a) has two pyridinic nitrogens that can both coordinate with Pd (or transition metal atoms) to form a metal-loaded COF that can act as single-atom catalysts for a variety of reactions, including Suzuki coupling.^65,66^ This COF requires two monomers: (1)­2,2′-dihydroxy-[1,1′-biphenyl]-3,3″,5,5′-tetracarbaldehyde and (2)­2,9-dimethyl-1,10-phenanthroline-3,8-diamine (or specified as linker100_CH and linker33_N by Mercado et al.) to form the imine linkage. The synthetic pathways of the two monomers are predicted exclusively by ASKCOS (see Figure 6b). For linker100_CH (restored to aldehyde termination), ASKCOS predicts its synthesis from two purchasable precursors: 2,2″-biphenol (**V100a**) and methenamine (**V100b**), which undergo a formylation reaction (Duff reaction^67^). The resulting intermediate **V100c** is brominated to produce intermediate **V100d**, which further reacts with dimethylformamide (DMF, purchasable) to yield the target monomer. For linker33_N (restored to amine termination), the route begins with purchasable neocuproine (**V33a**), then proceeds to nitration^68,69^ to introduce a nitro group. This active electrophile group is subsequently reduced to an amine to form intermediate **V33b**.^70^ The same nitration reaction is then repeated on **V33b**, accompanied by an additional protection–deprotection steps using ethyl chloroformate (**V33c**) to form a carbamate derivative on the first amine group, and ultimately yielding the restored monomer. In addition to the 2D kg m COF shown in Figure 6, a representative 3D COF with dia topology formed by linker100_CH and linker46_N is provided in Figure S8, together with the predicted retrosynthetic routes for both monomers.

**Figure 6:**
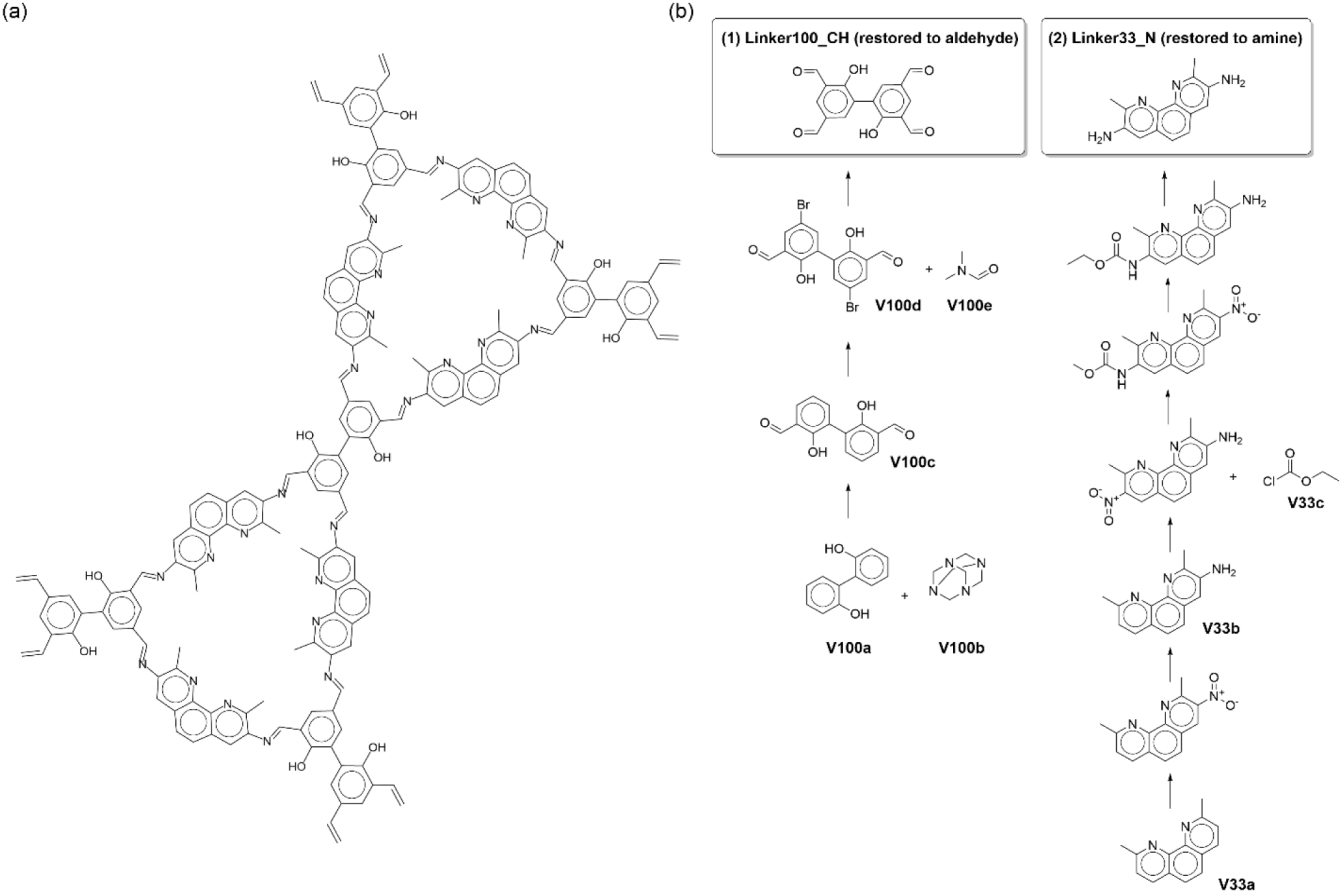
(a) A kg m COF formed by linker100_CH and linker33_N via imine linkage, a structure file (linker100_CH_linker33_N_kg m_relaxed.cif) can be referred to the Supporting Information of the literature by Mercado et al.^18^ Adapted from ref^17^. Copyright 2018 American Chemical Society. (b) The synthetic pathways of (1)­2,2′-dihydroxy-[1,1′-biphenyl]-3,3′,5,5′-tetracarbaldehyde (restored linker100_CH) and (2)­2,9-dimethyl-1,10-phenanthroline-3,8-diamine (restored linker33_N) with the highest route average plausibility, are predicted exclusively by ASKCOS.

## Conclusions

AI-based retrosynthesis tools were evaluated for their ability to identify known COF monomers. Cumulatively, these tools result in >70% success rate, motivating us to employ them to discover synthesis routes to unexplored monomers. To this end, synthesis routes to 83 unexplored monomers from a recent study on hypothetical COFs were successfully predicted; these monomers are likely to result in at least 1,200 hypothetical COFs.

Synthesis planning of COFs is currently largely a manual effort, heavily relying on the chemical intuition of the experimentalist; monomers are manually identified by disassembling the desired COF structure and synthesized using synthetic organic chemistry expertise.^23,47,51,54,55,57–59,71–75^ The synthesized monomers are then reticulated to form a crystalline framework by modulating a number of factors, such as the reactant concentrations, catalysts, solvents, temperature, amount of water, etc.^21,24,25^ We posit that accelerating the designed synthesis of COFs requires the adoption of automated tools, and AI and simulation algorithms. For instance, while identifying monomers for symmetric 2D/3D nets is relatively straightforward, simulations can help identify the right structures for more-complicated (less-symmetric) cases. In this context, prior work by Maula et al.,^76^ who employed Monte Carlo simulations of patchy particles, helps identify the right structure and chemical specificity needed to assemble monomers into crystalline COF for any topological net. Once such monomers are identified (using molecule generation techniques, generative methods,^77–79^ or reinforcement learning^80–83^ ), this work shows that their synthetic feasibility can be reliably evaluated for experimental validation. Once monomers are available, the synthesis of COF can benefit from autonomous tools^84–86^ that can employ techniques such as Bayesian optimization to obtain optimal conditions to maximize the crystallinity of the resulting framework. The detailed analysis in this work focused on 2D COF monomers, but the overall workflow is, in principle, extendable to 3D COF monomers. As described in the section of synthesizable unexplored COF, the unknown monomers can generate 39 distinct 3D COF topologies, with a representative dia example shown in Figure S8. While such an extension may involve additional challenges related to higher connectivity, stereochemical complexity, and conformational preferences, a systematic evaluation of known and hypothetical 3D COF monomers could be an important direction for future work. Overall, the present study represents a key step toward accelerating the discovery and construction of synthetically feasible COFs.

## Supplementary Material



## Notes

A list of all 359 COF monomer SMILES strings (text file). Predicted synthetic routes by two retrosynthesis tools (ASKCOS, AiZynthFinder) of all 359 COF monomers and all 109 virtual monomers (zip files). Context in Supporting Information (PDF) including: a collection of supplementary figures : (1) Scaffold images of the top 20 most frequently used COF monomers, (2) Synthetic routes of TAPP (mol-20), (3) the distribution of synthetic accessibility scores (SAscore) for all 359 monomers, and (4) 109 fictitious monomers categorized as solvable or unsolvable by two retrosynthesis tools. An excel list of the 296 2D COFs literature references. An excel list of the 843 extracted monomers and a folder of their corresponding JSON files generated by MolScribe. Clustering ramp test and a table of noise levels and the least Dice similarity score for each configuration. Additional molecular feature discussions by cluster and the monomers image by clusters. Monomers structure analysis by GPT-4o, including the prompt and all 17 responses generated by GPT-4o. Configuration files for the two retrosynthesis tools and an additional case study for solved and unsolved monomers. JSON files of retrosynthesis-predicted routes for all 359 known monomers and 109 virtual monomers. A list of the 359 unique monomers and their index. A list of the 666 virtual monomer SMILES strings, and their restored IUPAC name (ZIP)
