# Supplementary material for: AI-Based Retrosynthesis of Known and Unexplored Covalent Organic Framework Monomers
Source: Chem Mater. 2026 Jul 29;38(15):7579–89. doi: 10.1021/acs.chemmater.6c00008 (PMC13484925; doi:10.1021/acs.chemmater.6c00008)
Supplement: Supplementary file 1 [file cm6c00008_si_001.zip › SI-main/SI_main.pdf]

Supplementary Information

AI-based retrosynthesis of known and unexplored covalent organic framework monomers

Chin-Fei Chang and Srinivas Rangarajan*

Department of Chemical and Biomolecular Engineering, Lehigh University, 124 E Morton Street, Bethlehem PA 18015

Contents

S1 Supplementary Figures **2**

S2 COF Monomer Extraction **10**

S3 Clustering Analysis of the 359 Unique Monomers **11**

S3.1 Clustering Ramp Test 11

S3.2 Molecular Feature Discussion by Cluster 13

S4 Monomers Structure Analysis by GPT-4o **31**

S5 Parameter Setting and Results of Retrosynthesis Analysis **40**

S5.1 Additional Case Study for Solved/Unsolved Monomers 40

S5.1.1 Solved Monomer 40

S5.1.2 Unsolved Monomer 41

*Corresponding author. Email: srr516@lehigh.edu

S1 Supplementary Figures

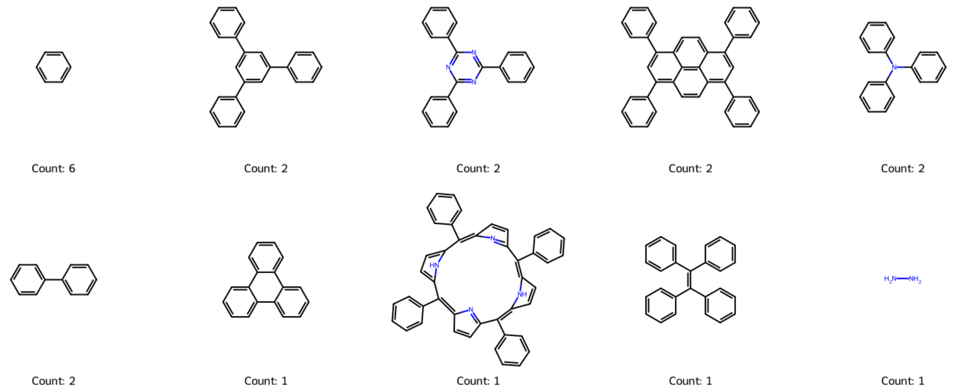

Figure S1: The scaffolds of the top 20 frequently used monomers in our collected 2D COF literatures. The usage count is labeled under each scaffold. These scaffolds were extracted using RDKit, as described in Methods Section 2.1. All scaffolds contain at least one aromatic ring, highlighting the dominance of conjugated systems in COF monomer design. An exception is hydrazine (bottom-right), which represents the shortest linear linker identified.

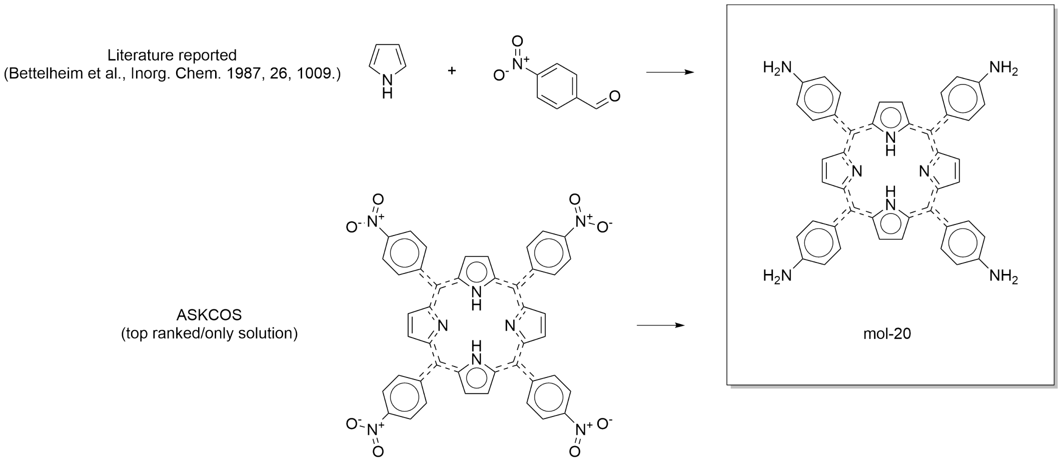

Figure S2: Synthetic routes comparison for tetra(p-aminophenyl)porphyrin (TAPP, mol-20), a solved monomer in cluster 0. The top synthetic route is reported by Bettelheim et al.[1], where TAPP was originally synthesized from pyrrole and p-nitrobenzaldehyde. In ASKCOS, the predicted route uses commercially available porphyrin-based precursor and reduce the nitro groups to amine groups which yield the TAPP. AiZynthFinder did not provide a solution for this compound.

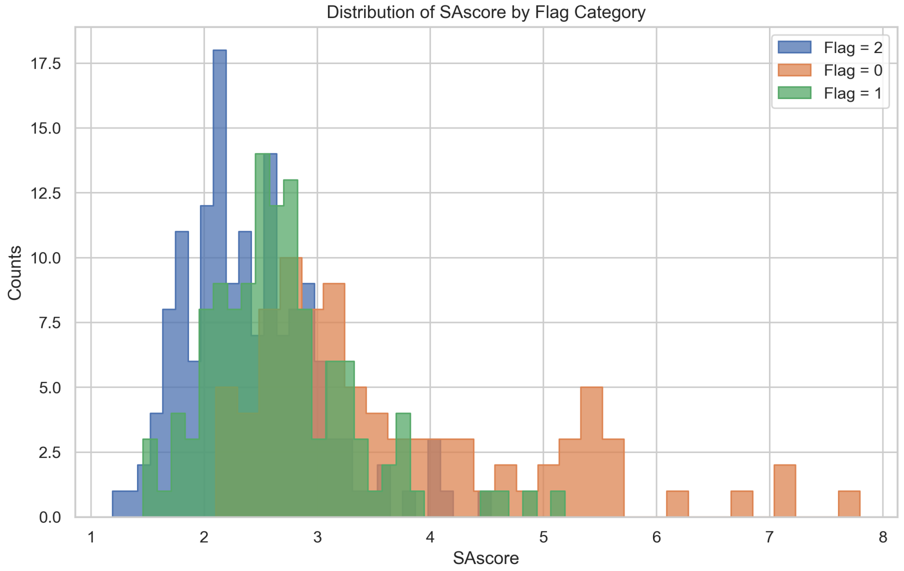

Figure S3: The distribution of synthetic accessibility scores (SAscore) for all 359 monomers, grouped by their retrosynthesis flag categories. SAscore ranges from 1 (easiest to synthesize) to 7 (most difficult). Monomers solvable by both ASKCOS and AiZynthFinder (blue, flag = 2) have the lowest average score of 2.4. Monomers solvable by only one tool (green, flag = 1) have an intermediate average of 2.7, while unsolved monomers (orange, flag = 0) have the highest average score of 3.7. This trend is comparable to the solvable/unsolvable distribution of 100 ChEMBL compounds reported by Genheden et al.[3], which supports the notion that structural simplicity strongly influences the success of retrosynthetic predictions.

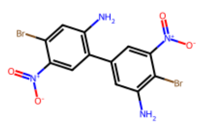

18_Br

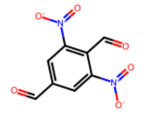

6_COH

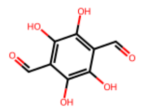

12_COH

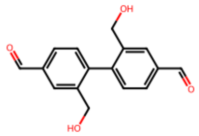

14_COH

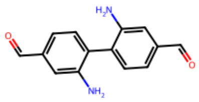

16_COH

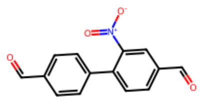

22_COH

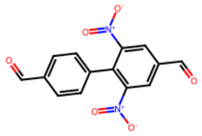

24_COH

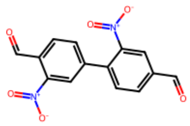

25_COH

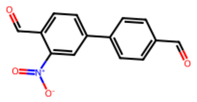

28_COH

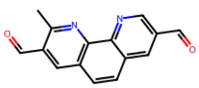

36_COH

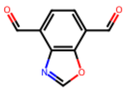

41_COH

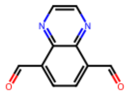

45_COH

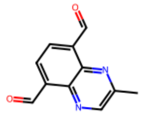

46_COH

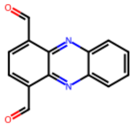

47_COH

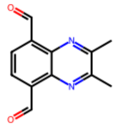

48_COH

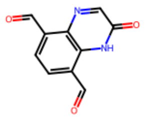

49_COH

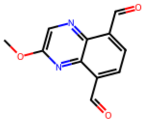

52_COH

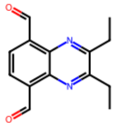

53_COH

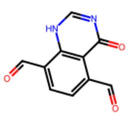

57_COH

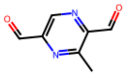

59_COH

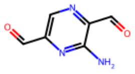

61_COH

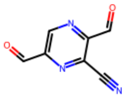

62_COH

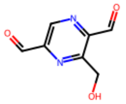

63_COH

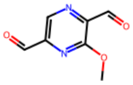

66_COH

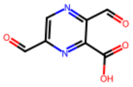

67_COH

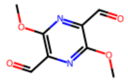

70_COH

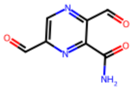

71_COH

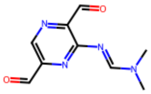

73_COH

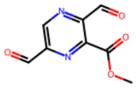

74_COH

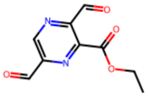

75_COH

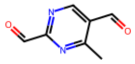

77_COH

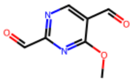

78_COH

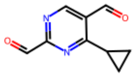

79_COH

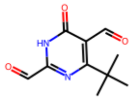

80_COH

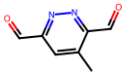

82_COH

Figure S4: Of the 109 novel monomers for hypothetical COFs, 47 are solvable by both ASKCOS and AiZynthFinder. Each molecule is labeled with its index (see Tables S4- S6) and the restored functional group.

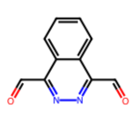

83_COH

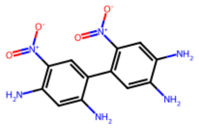

17_NH2

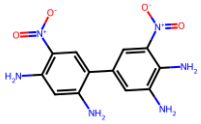

18_NH2

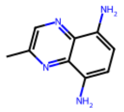

46_NH2

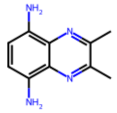

48_NH2

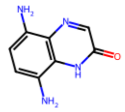

49_NH2

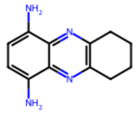

51_NH2

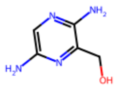

63_NH2

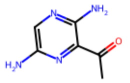

69_NH2

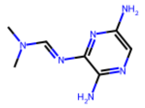

73_NH2

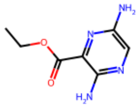

75_NH2

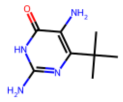

80_NH2

Figure S4(continued): Of the 109 novel monomers for hypothetical COFs, 47 are solvable by both ASKCOS and AiZynthFinder. Each molecule is labeled with its index (see Tables S4-S6) and the restored functional group.

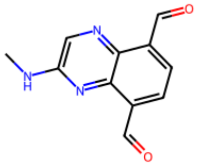

50_COH

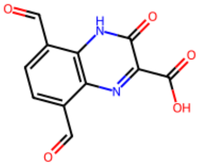

54_COH

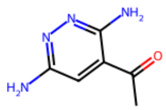

85_NH2

Figure S5: In addition to the 47 monomers in Figure S4, these 3 are solvable by AiZynthFinder but not ASKCOS. Each is labeled with its index (see Tables S4–S6) and the restored functional group.

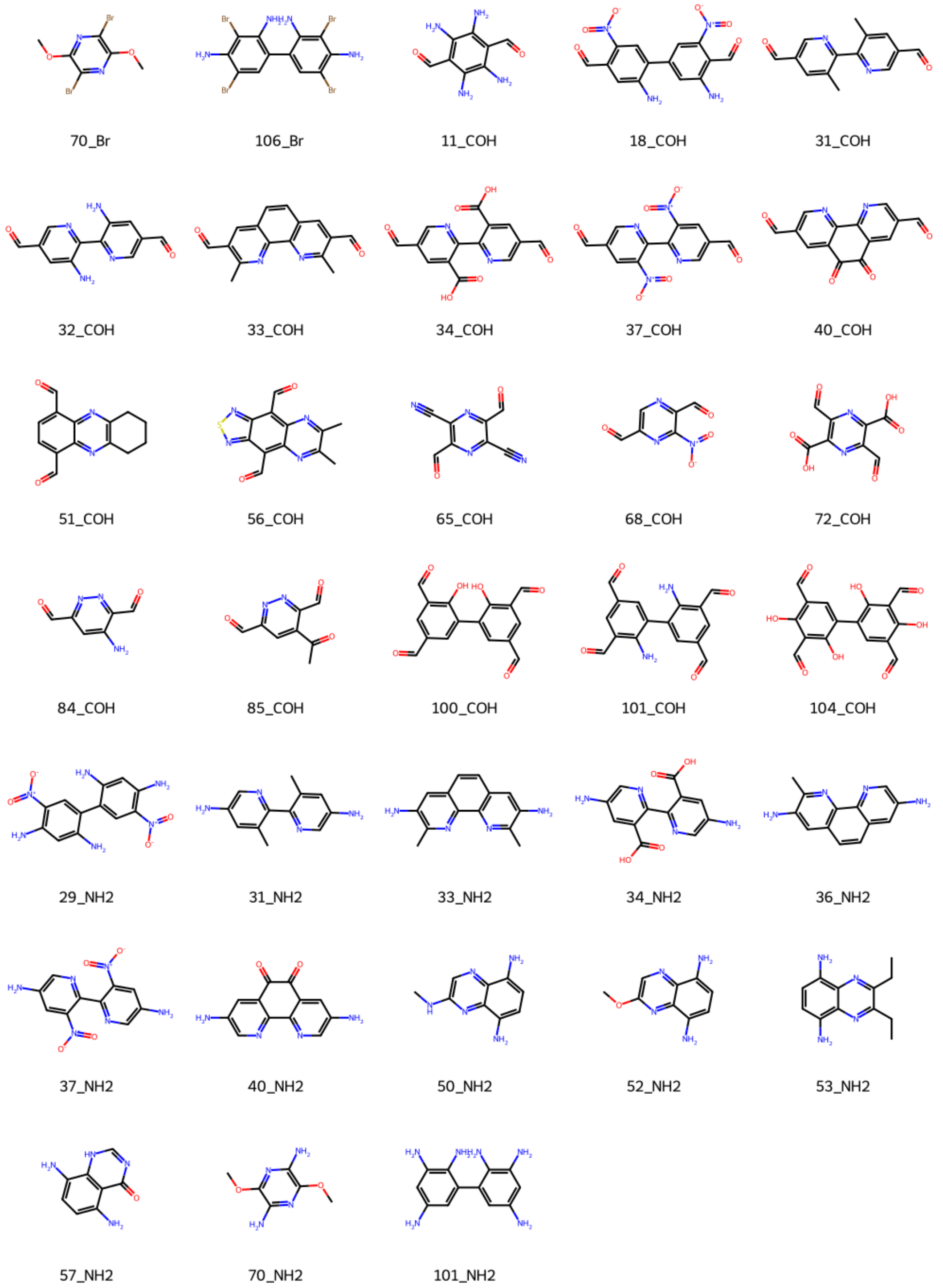

Figure S6: In addition to the 47 monomers in Figure S4, these 33 are solvable by ASKCOS but not AiZynthFinder. Each is labeled with its index (see Tables S4–S6) and the restored functional group.

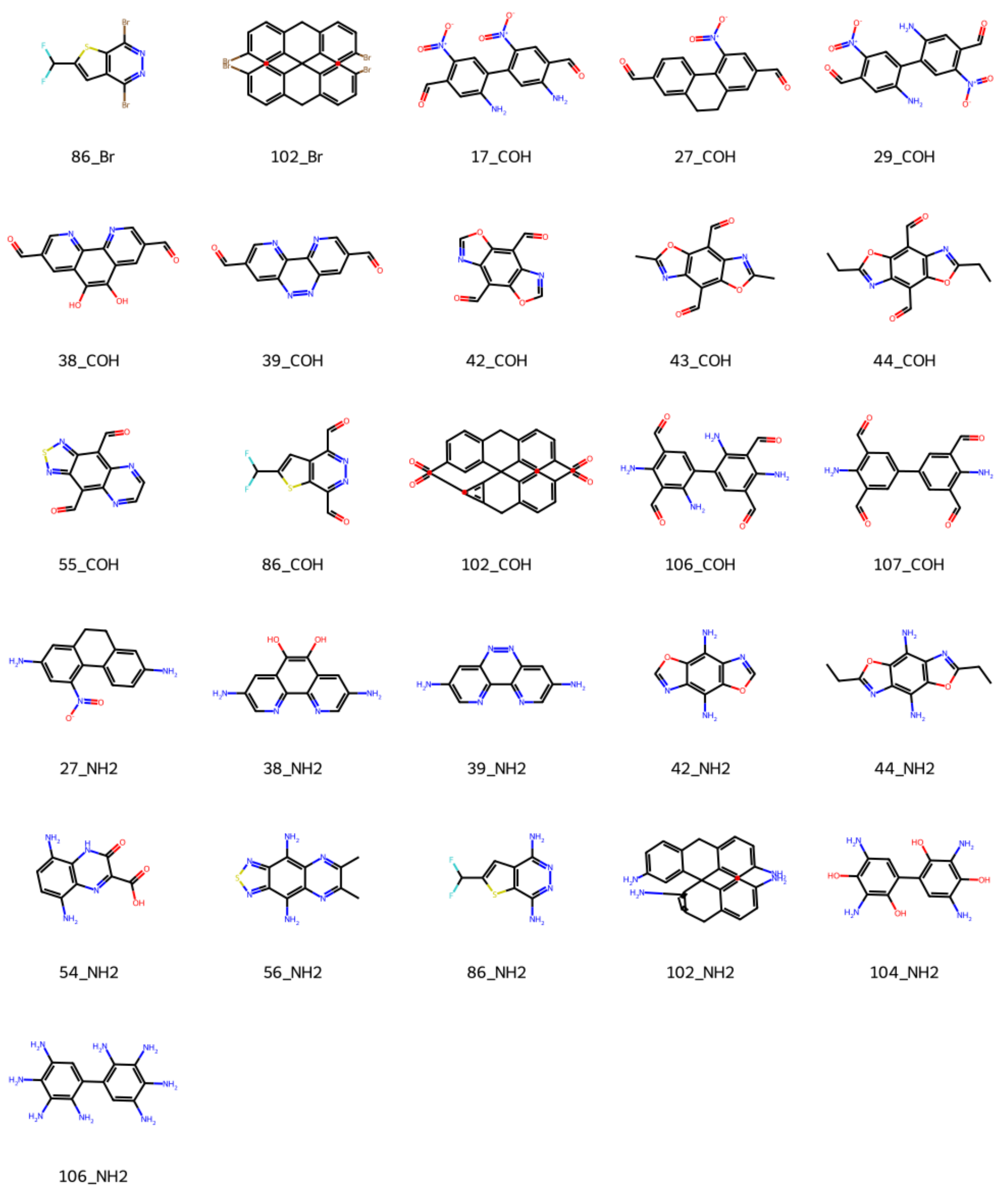

Figure S7: Of the 109 novel monomers for hypothetical COFs, 26 are unsolvable by both ASKCOS and AiZynthFinder. Each molecule is labeled with its index (see Tables S4- S6) and the restored functional group.

(a)

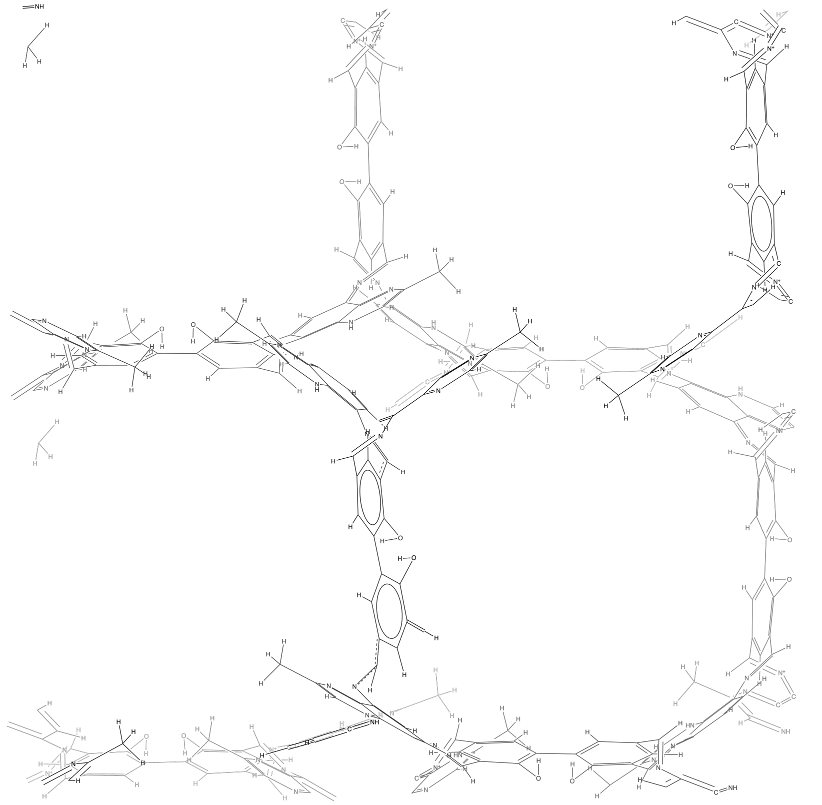

(b)

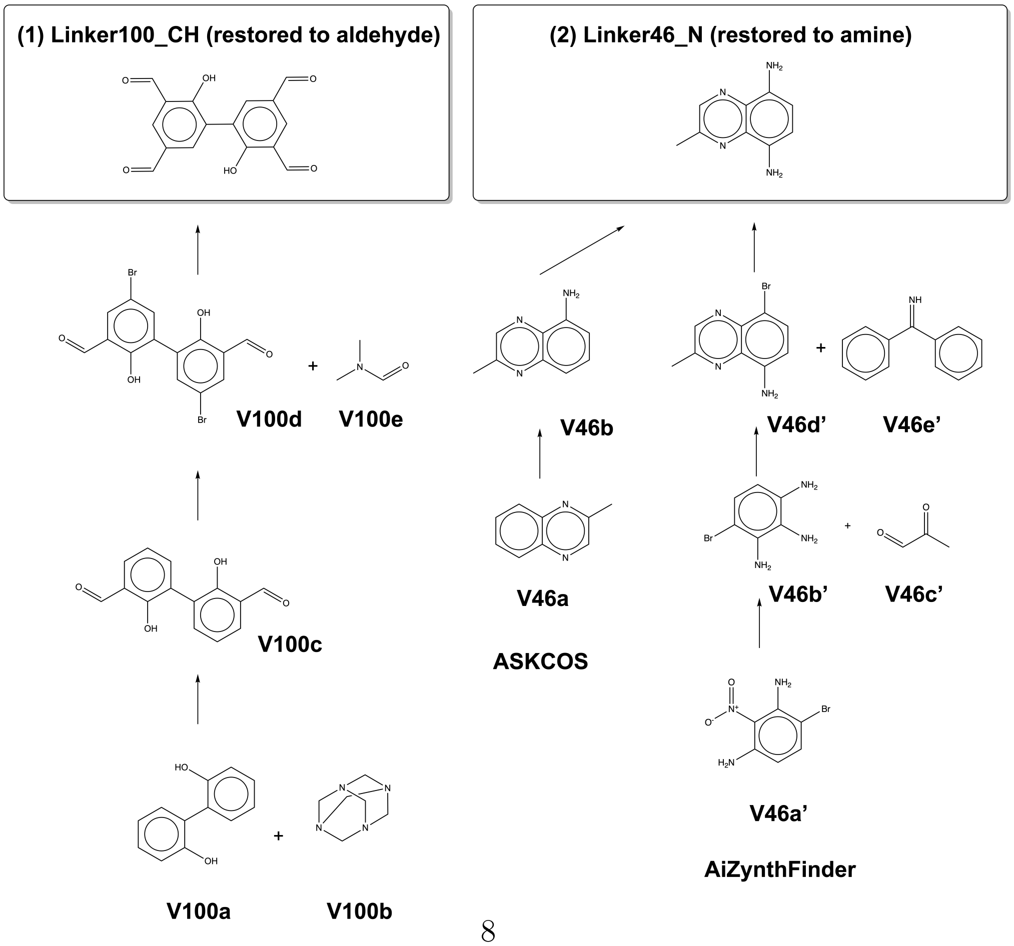

(1) Linker100_CH (restored to aldehyde)
(2) Linker46_N (restored to amine)
V100d
V100e
V100c
V100a
V100b
V46b
V46a
ASKCOS
V46d’
V46e’
V46b’
V46c’
V46a’
AiZynthFinder

Figure S8: (continued) (a) A 3D dia COF formed by linker100_CH and linker46_N via imine linkage. The structure file `linker100_CH_linker46_N_dia_relaxed.cif` can be found in the Supporting Information of Mercado et al.[6]. Adapted from ref 6. Copyright 2018 American Chemical Society. (b) Predicted synthetic pathways for (1) 2,2'-dihydroxy-[1,1'-biphenyl]-3,3',5,5'-tetracarbaldehyde (restored linker100_CH), which is identical to the route shown in Figure 6, and (2) 2-methylquinoxaline-5,8-diamine (restored linker46_N). Linker100_CH was predicted exclusively by ASKCOS, whereas linker46_N was solved by both ASKCOS and AiZynthFinder; one representative route from each tool is shown. For the restored linker46_N (2-methylquinoxaline-5,8-diamine), both ASKCOS and AiZynthFinder predict feasible multistep syntheses (Figure S8(b)). In the ASKCOS route, purchasable 2-methylquinoxaline (V46a) undergoes two steps of aromatic ring functionalization, transforming V46a into V46b and ultimately yielding 2-methylquinoxaline-5,8-diamine. In contrast, AiZynthFinder proposes a route beginning from 4-bromo-2-nitrobenzene-1,3-diamine (V46a′), in which the nitro group is first reduced to form 4-bromobenzene-1,2,3-triamine intermediate V46b′. Intermediate V46b′ then reacts with methylglyoxal (V46c′) to construct the quinoxaline ring and generate brominated intermediate V46d′. Finally, the bromine substituent is removed through hydrogenolysis using benzophenone imine (V46e′) as a coupling partner, affording the target monomer 2-methylquinoxaline-5,8-diamine.

S2 COF Monomer Extraction

A list of literature where the 2D COFs monomers were extracted is provided in the file `All_2D_COF_paper_index.xls`. Each article and its supplementary information (SI) is indexed using an article ID and a suffix: "0" refers to the main article and "1" refers to the SI document.

All the 843 extracted monomers are compiled in `2D_COF_843_mols.xlsx`. Each molecule is labeled using the format `mol-XXX-Y-ZZ`, where `XXX` indicates the article ID, `Y` represents the suffix (0 for main text and 1 for SI), and `ZZ` is the molecule's sequence number within the same article.

The spreadsheet includes three columns related to SMILES. The `detect_smiles` column contains the raw SMILES strings generated by MolScribe. The `corrected_smiles` column shows manually corrected versions of these SMILES. The `RDKit_clean` column contains the final validated SMILES strings that are successfully processed by RDKit. If the `RDKit_clean` entry is empty, the molecule is considered unprocessable by RDKit and is likely incompatible with retrosynthesis tools. The `flag` column notes if the molecule is solved 2 = by both ASKCOS and AiZynthFinder, 1 = by either one tool, or 0 = not solved.

All JSON files generated by MolScribe for these molecules are available in the folders `MolScribe_translate`.

S3 Clustering Analysis of the 359 Unique Monomers

S3.1 Clustering Ramp Test

To find suitable clustering parameters, we performed a parameter ramp test using (1) UMAP[5] to reduce molecular fingerprints to two dimensions and (2) HDBSCAN[2] to assign clusters. The number of neighbors (n_neighbors) and minimum distance (min_dist) were varied from 5 to 100 (y-axis in Figure S9) and 0 to 1 (x-axis in Figure S9), respectively, to assess their impact on cluster formation. HDBSCAN was applied with fixed settings: min_cluster_size = 5 and the `manhattan` metric.

Figure S9 shows the clustering results for each parameter combination. Table S1 lists the number of clusters and the number of noise points (monomers not assigned to any cluster). The column "Least Dice Similarity" refers to the lowest Dice similarity score within clusters at each setting, excluding the noise cluster.

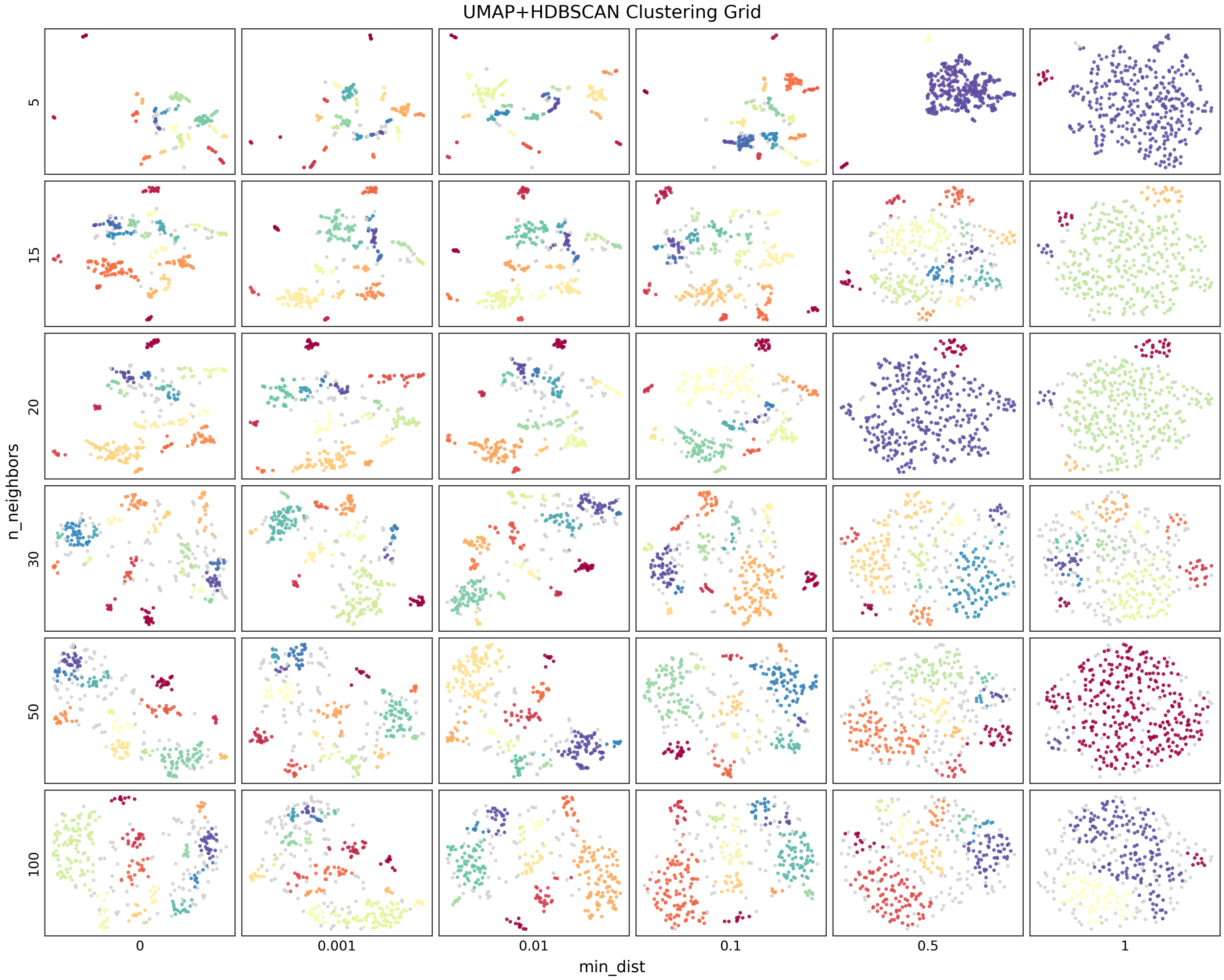

Figure S9: Clustering ramp test on the 359 monomers using UMAP and HDBSCAN with different combinations of n_neighbors (y-axis) and min_dist (x-axis). Parameters of HDBSCAN are fixed: min_cluster_size = 5 and metric = `manhattan`.

Table S1: Summary of clustering ramp test results on the 359 monomers using various combinations of UMAP n_neighbors and min_dist. For each combination: (1)the number of clusters assigned by HDBSCAN,(2) the number of monomers not assigned to any cluster (noise) and (3) the lowest Dice similarity score of all clusters (excluding noise cluster) are reported. The Dice similarity score measures structural similarity of all monomers clustering in the same group.

`n_neighbors`	`min_dist`	**Clusters**	**Noises**	**Least Dice Similarity***
5	0	23	30	0.303
	0.001	24	39	0.307
	0.01	20	19	0.307
	0.1	20	31	0.291
	0.5	3	0	0.179
	1	2	2	0.181
15	0	20	35	0.315
	0.001	17	22	0.317
	0.01	15	28	0.293
	0.1	21	53	0.327
	0.5	12	81	0.273
	1	4	12	0.190
20	0	17	42	0.276
	0.001	15	38	0.330
	0.01	15	60	0.291
	0.1	13	35	0.267
	0.5	2	2	0.188
	1	4	19	0.191
30	0	20	92	0.330
	0.001	12	43	0.257
	0.01	14	55	0.222
	0.1	14	51	0.273
	0.5	9	82	0.285
	1	11	151	0.284
50	0	13	88	0.288
	0.001	15	96	0.287
	0.01	11	49	0.288
	0.1	12	67	0.307
	0.5	10	108	0.290
	1	2	49	0.190
100	0	12	90	0.179
	0.001	16	98	0.306
	0.01	11	64	0.286
	0.1	12	65	0.274
	0.5	9	106	0.259
	1	3	124	0.275

We selected the configuration n_neighbors = 15 and min_dist = 0.001, as discussed in the Method section 2.1, because it produced the fewest noise points (22 monomers) and all clusters had Dice similarity scores greater than 0.3. Detailed statistics for each cluster, including the number of molecules, retrosynthesis flag (0 = unsolved by both tools, 1 = solved by one, 2 = solved by both), and success rate, are shown in Table S2.

Table S2: Cluster statistics for the selected parameters (n_neighbors = 15, min_dist = 0.001): molecule count, retrosynthesis flag distribution, and success rate are shown.

**Cluster**	**Dice Similarity**	**Molecule Count**	**Flag 0**	**Flag 1**	**Flag 2**	**Ratio**
-1	0.16	22	7	9	6	68%
0	0.66	10	7	3	0	30%
1	0.66	10	3	1	6	70%
2	0.58	11	3	3	5	73%
3	0.33	22	5	3	14	77%
4	0.34	34	4	9	21	88%
5	0.34	11	3	7	1	73%
6	0.45	11	4	4	3	64%
7	0.42	70	20	29	21	71%
8	0.47	6	1	3	2	83%
9	0.53	15	3	5	7	80%
10	0.62	14	2	5	7	86%
11	0.39	14	0	2	12	100%
12	0.34	68	14	29	25	79%
13	0.42	7	1	1	5	86%
14	0.32	6	2	2	2	67%
15	0.39	12	6	3	3	50%
16	0.35	16	2	4	10	88%

S3.2 Molecular Feature Discussion by Cluster

The monomers in each cluster are shown in Figure S10 to S27. The legend under each molecule stands for the retrosynthesis flag: if the molecule is solved 2 = by both ASKCOS and AiZynthFinder, 1 = by either one tool, or 0 = not solved.

Below is the extension of the manual cluster-wise molecular feature discussion in Section 3.1.

Porphine, benzaldehyde with ethyne(s) and 4,7-diphenyl-1H-benzimidazole are found as the main backbone for monomers in cluster 0, 1 and 2 respectively. Acetonitrile (or benzonitrile) is the dominant functional group possessed by monomers in cluster 3.

Monomers in cluster 4, 6 and 7 primarily possess aldehyde-rich benzene derivatives, with additional hydroxyl group(s) presented in cluster 4 and a major thiophene backbone observed in cluster 6. Cluster 5 contains monomers with dianhydride (or 2,4-cyclohexadienone) and tetraone fragments.

Clusters 8 and 9 are spatially close in the UMAP plot and share characteristics of boronic

acids; cluster 8 includes thiopheneboronic acids, while cluster 9 has boronic acids on benzene rings. An exception in cluster 8 is 2,5-dihydrazino-1,3,4-thiadiazole, the only unsolved monomer in that group; although its amine groups suggest it fits better in cluster 12 or 16, it may have been grouped here due to its thiophene scaffold.

Clusters 10 and 11 share benzhydrazide fragments and are located near each other in UMAP plot. Cluster 10 primarily consists of terephthalohydrazide derivatives with substitutions like diethoxy or nonafluorohexyl, while cluster 11 includes benzamide, phenylthiourea, and pyridyl benzoate structures.

Diamine (or aniline) groups are common in clusters 12, 13, 16 and the triphenylene-2,3,6,7,10,11-hexamine which may have been misclassified into cluster 15. In addition to amines, cluster 13 features nitro, carboxylic acid, or sulfonic acid groups, and cluster 16 includes o-toluidine. Lastly, most monomers in cluster 15 contain catechol or hydroquinone, aside from the above-mentioned exception.

Most clusters properly capture molecular features and discrete the monomers reasonably, however, mediocre performance is noticed in cluster 14 where the centroid molecule does not fully represent the whole monomers well, as part of the monomers (benzenehexamine, melamine and 2,2'-bipyridine-5,5'-diamine) could be merged in either cluster 12 or 16 where they shared the identical molecular features (amine).

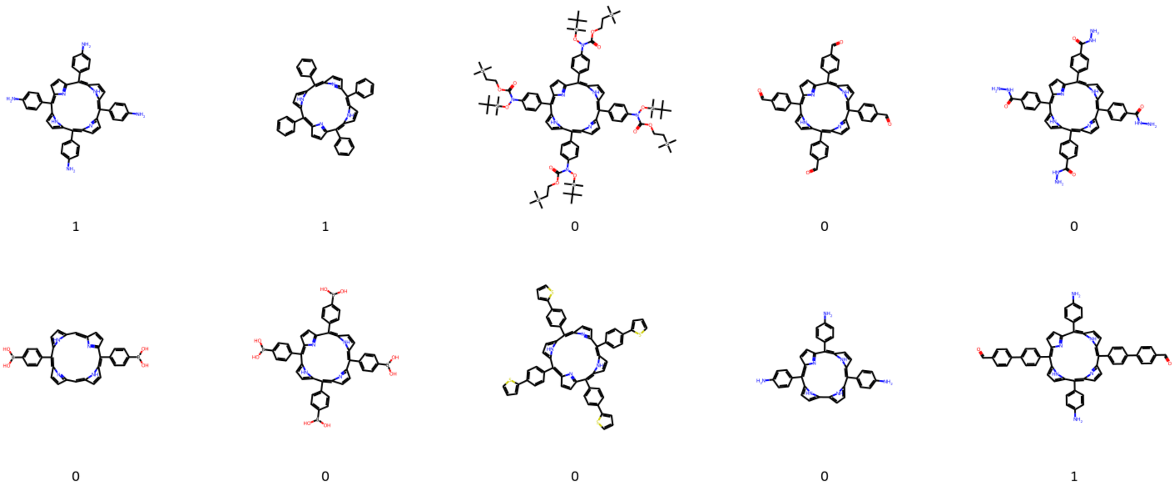

Figure S10: Cluster 0

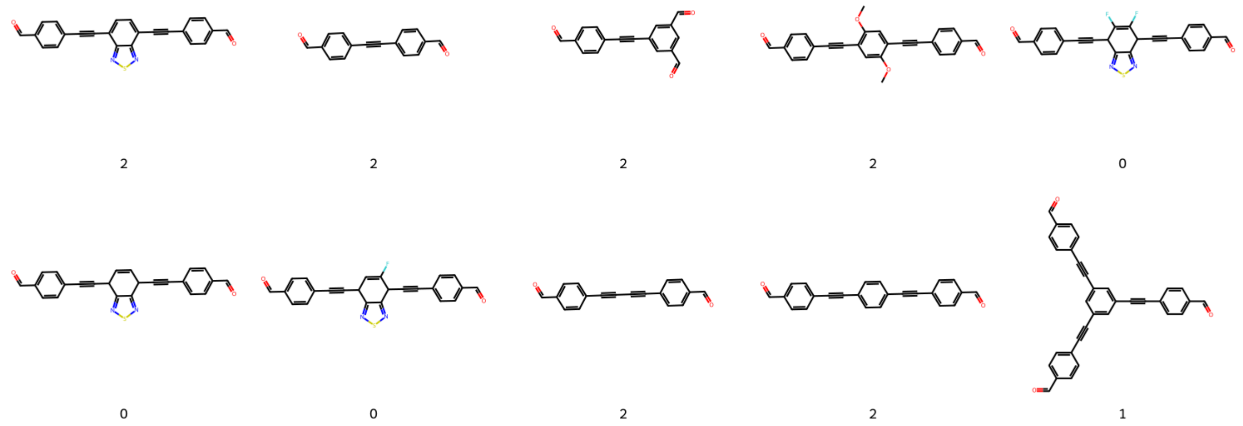

Figure S11: Cluster 1

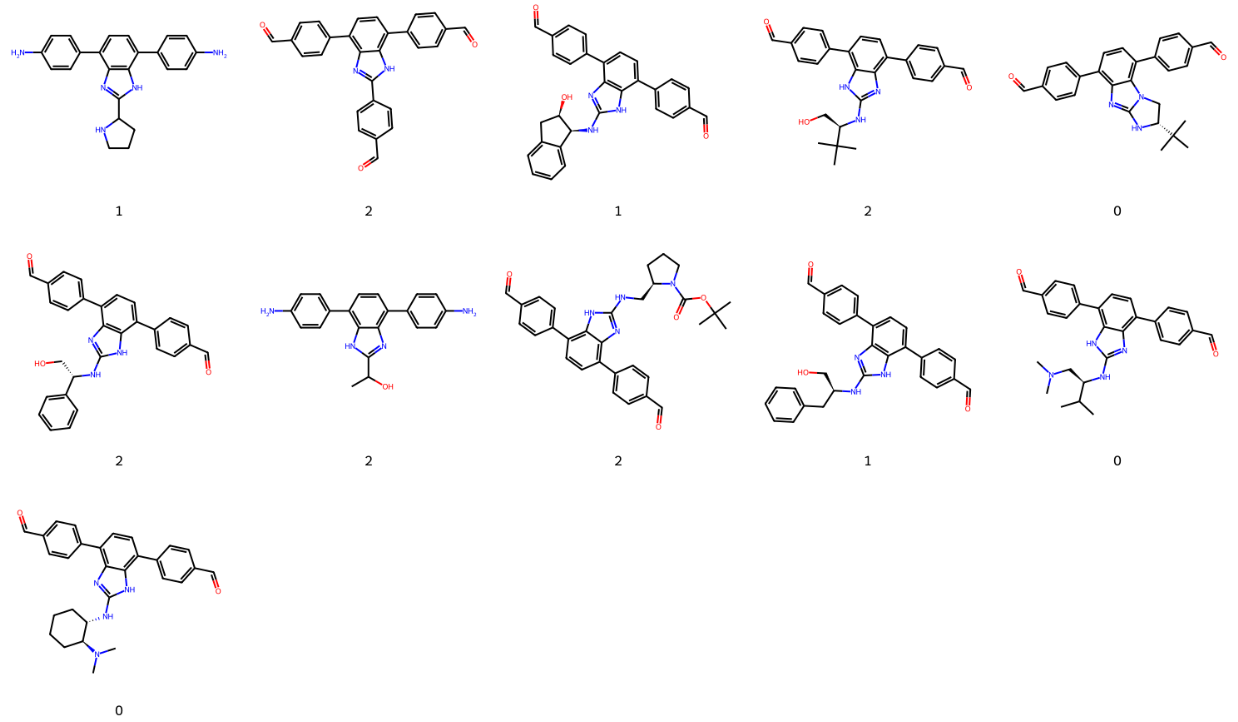

Figure S12: Cluster 2

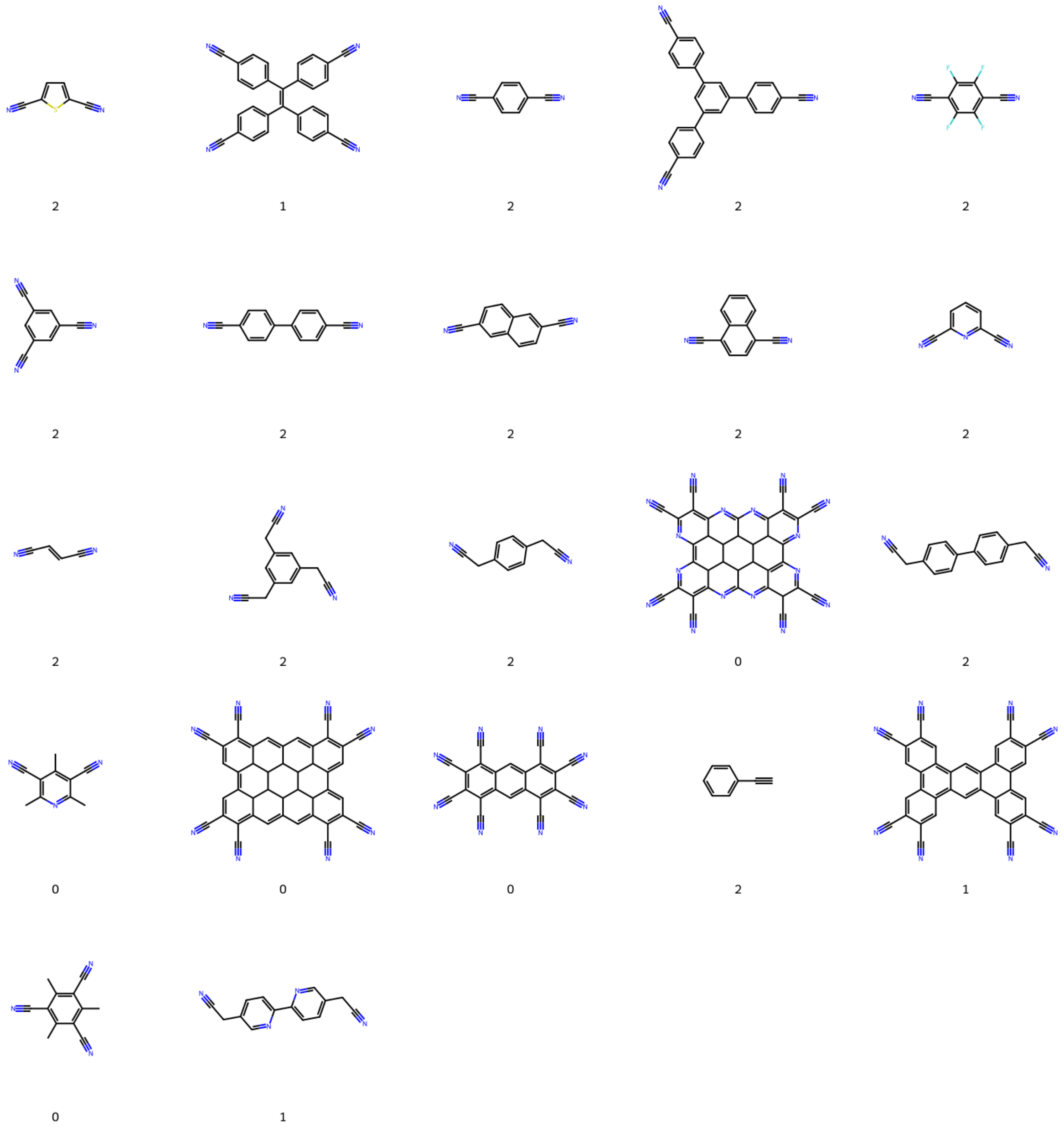

Figure S13: Cluster 3

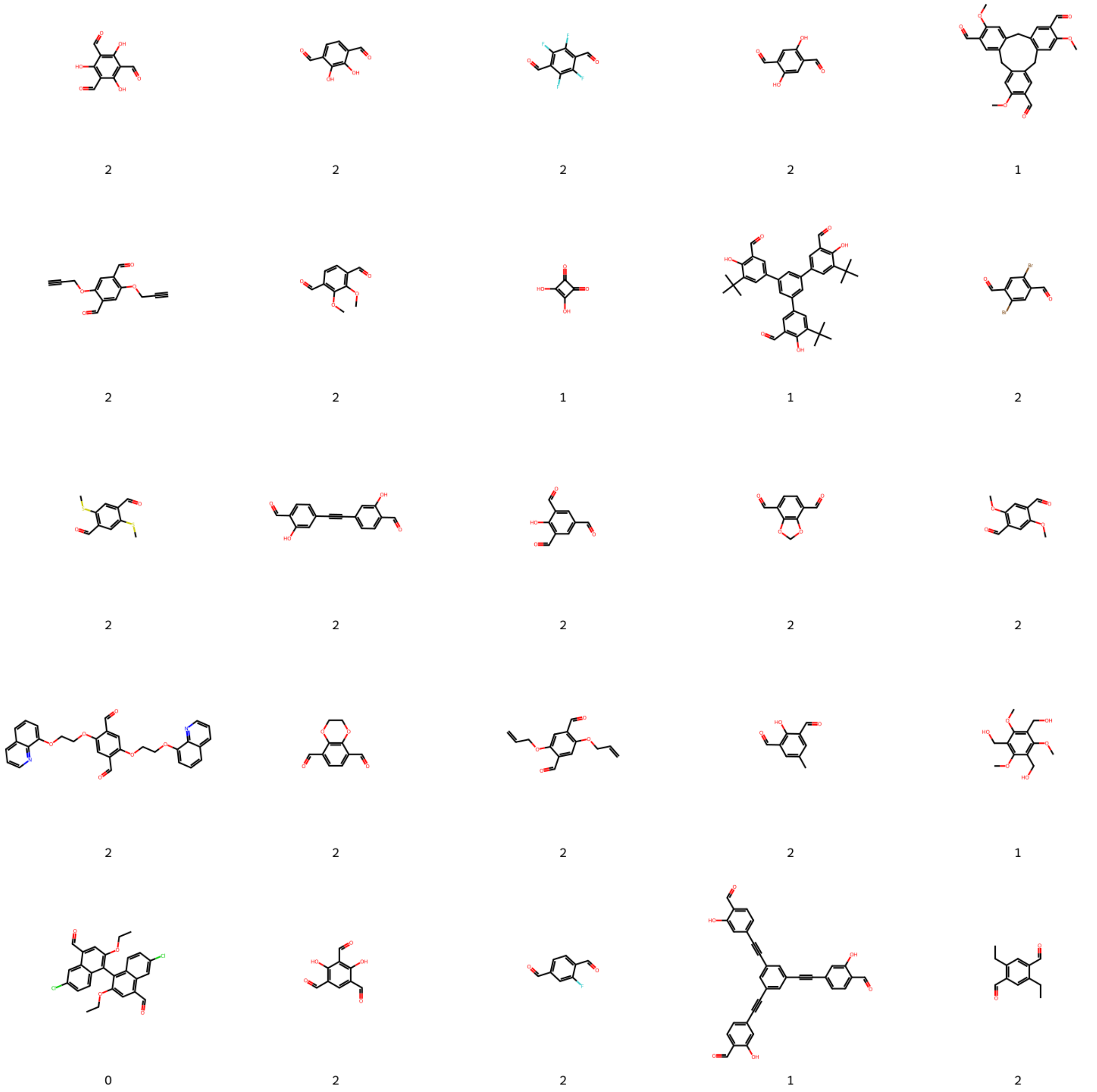

Figure S14: Cluster 4

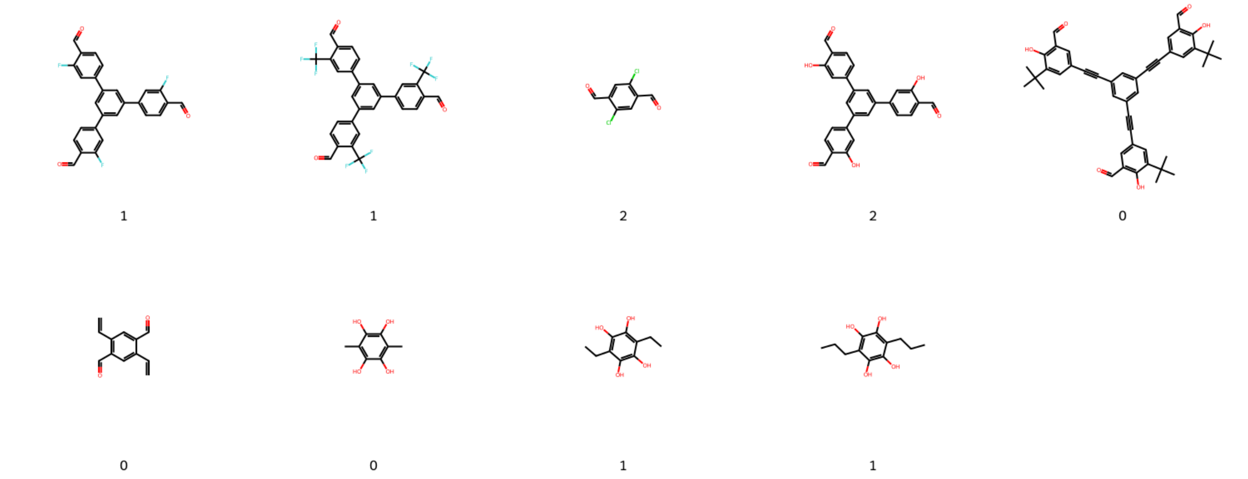

Figure S14(continued): Cluster 4

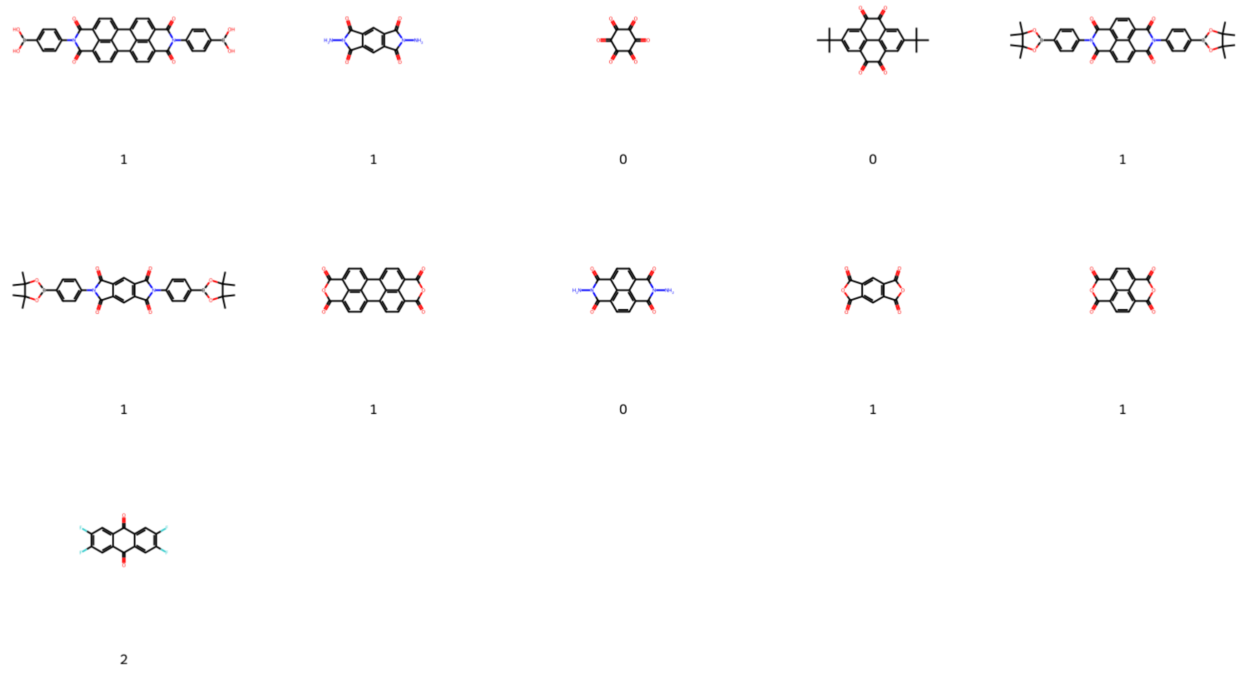

Figure S15: Cluster 5

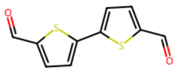

2

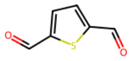

2

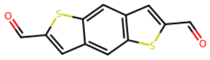

2

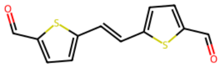

1

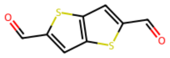

1

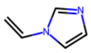

1

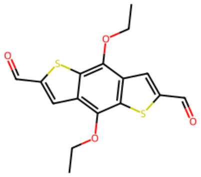

0

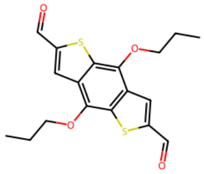

0

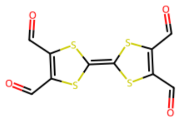

0

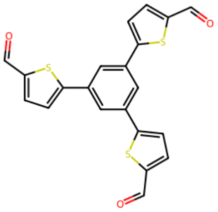

1

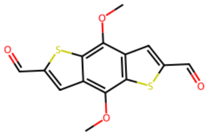

0

Figure S16: Cluster 6

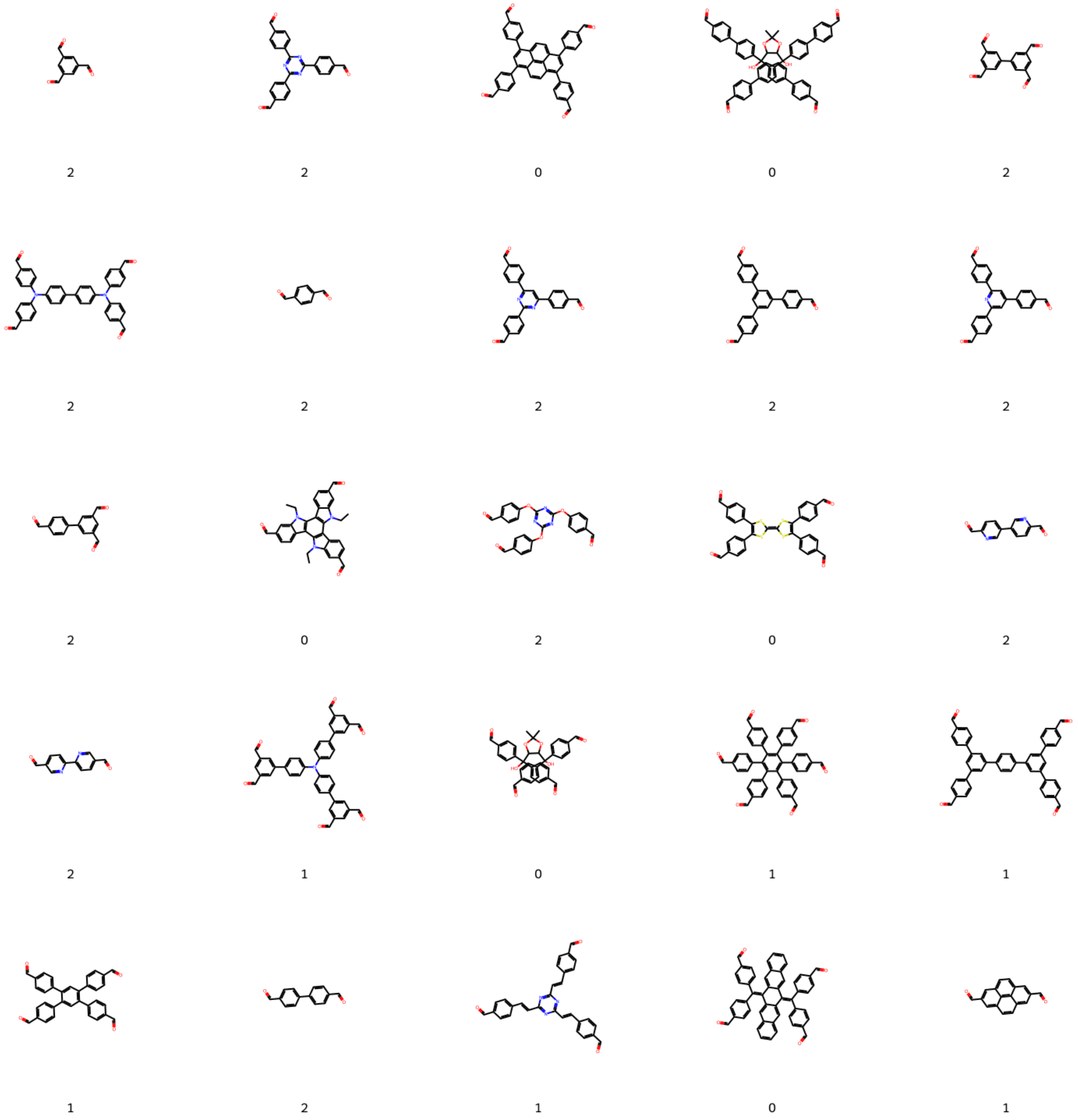

Figure S17: Cluster 7

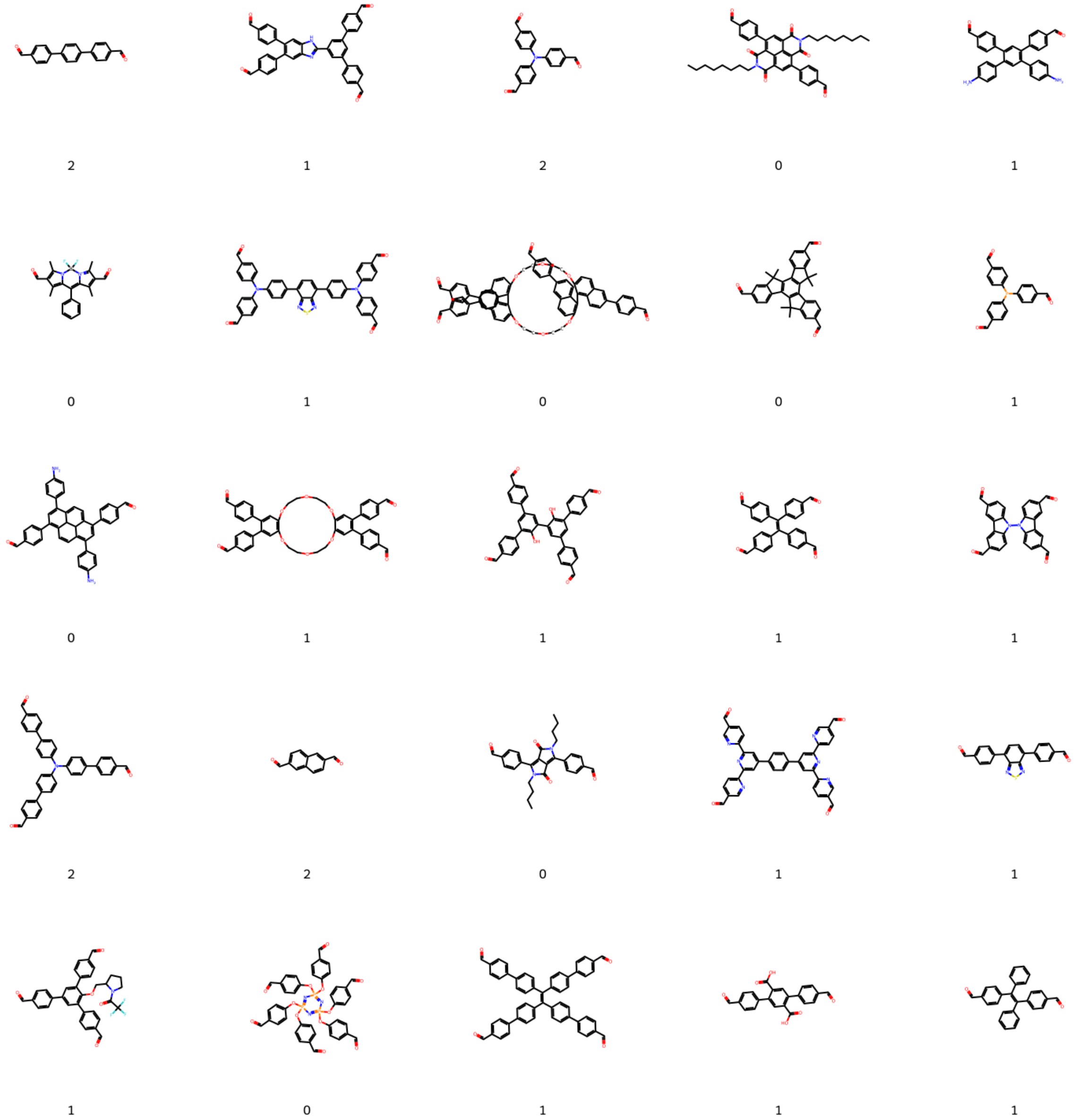

Figure S17(continued): Cluster 7

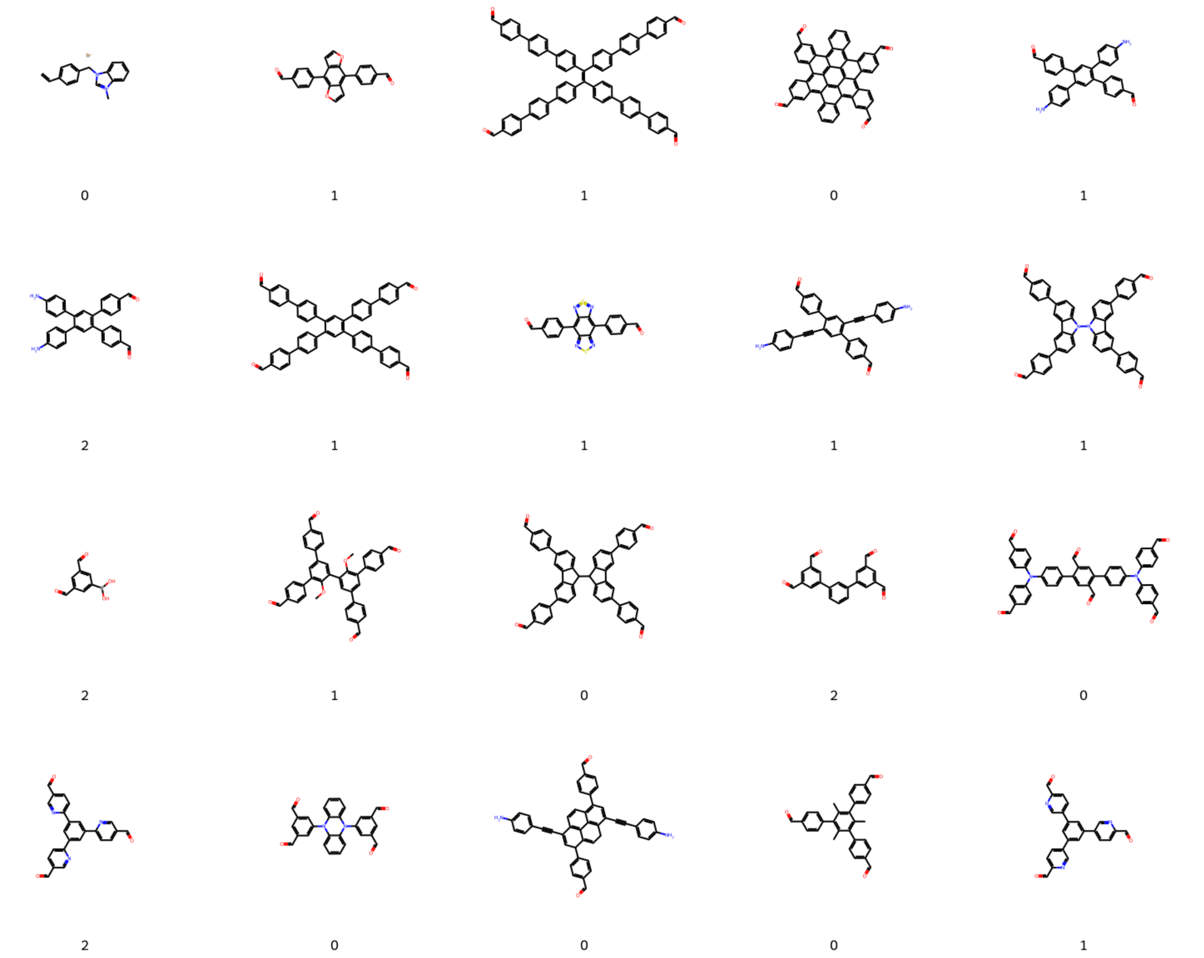

Figure S17(continued): Cluster 7

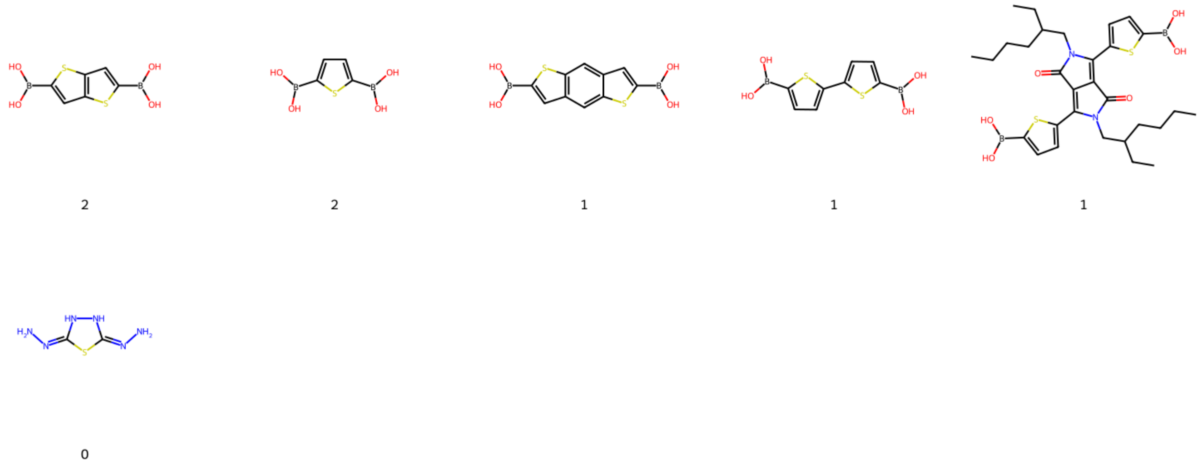

Figure S18: Cluster 8

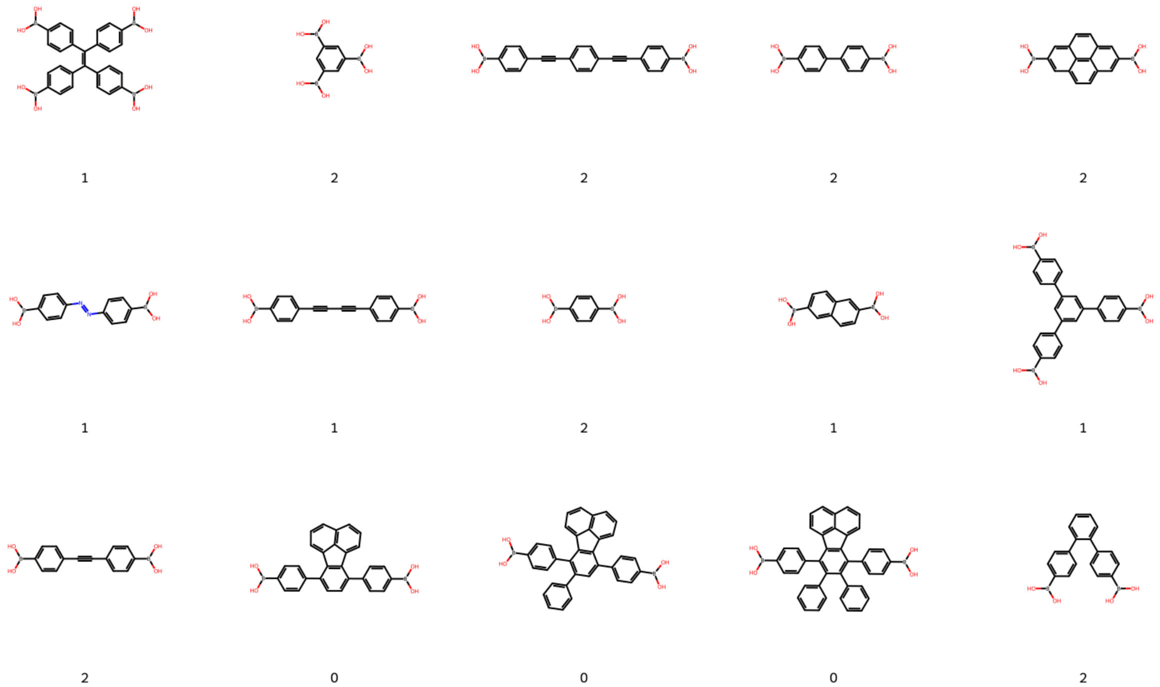

Figure S19: Cluster 9

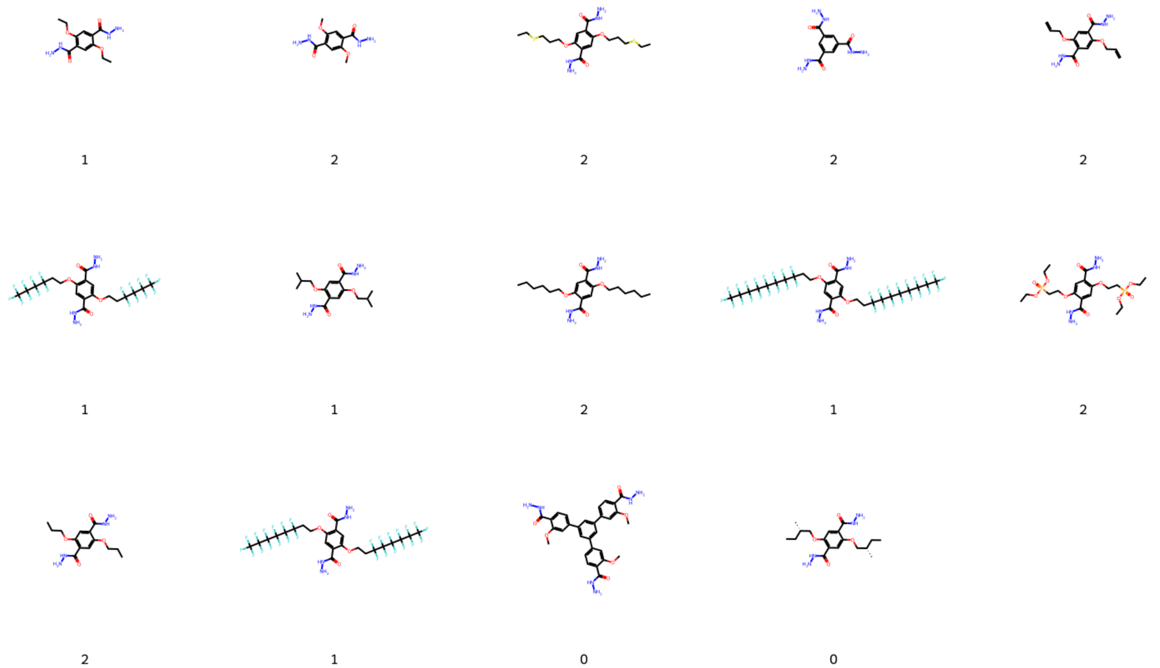

Figure S20: Cluster 10

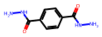

2

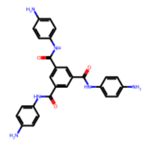

2

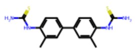

2

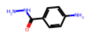

2

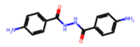

2

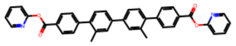

2

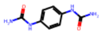

2

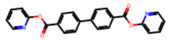

1

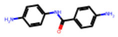

2

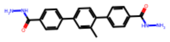

2

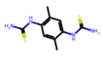

2

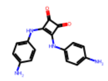

2

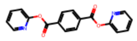

1

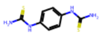

2

Figure S21: Cluster 11

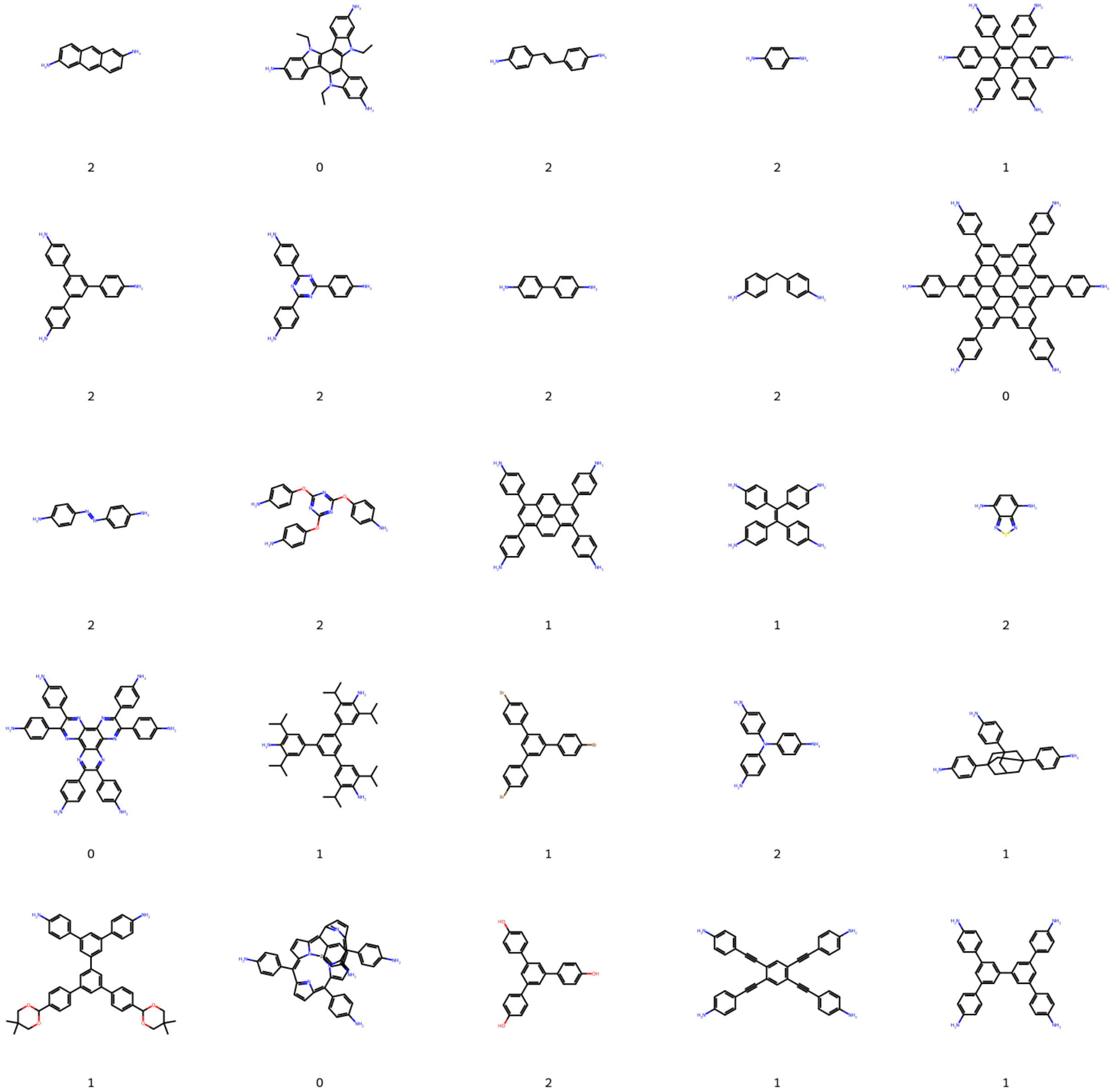

Figure S22: Cluster 12

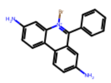

0

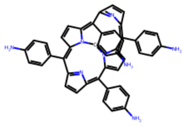

0

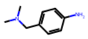

2

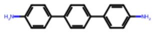

2

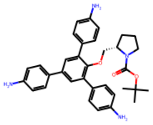

2

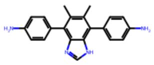

1

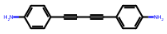

2

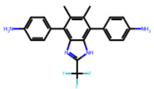

1

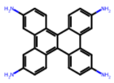

0

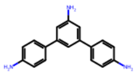

2

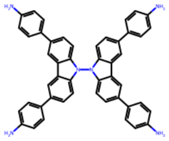

1

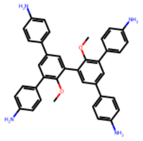

1

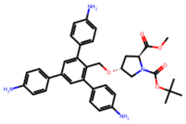

1

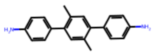

2

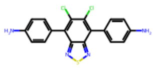

1

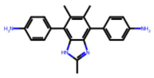

2

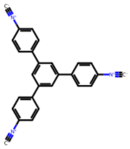

1

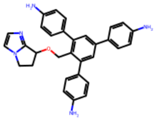

1

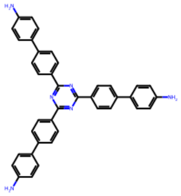

1

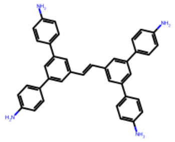

1

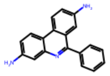

1

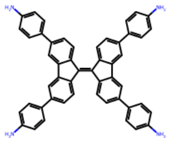

1

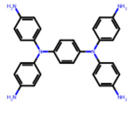

2

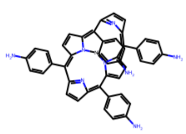

0

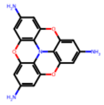

0

Figure S22(continued): Cluster 12

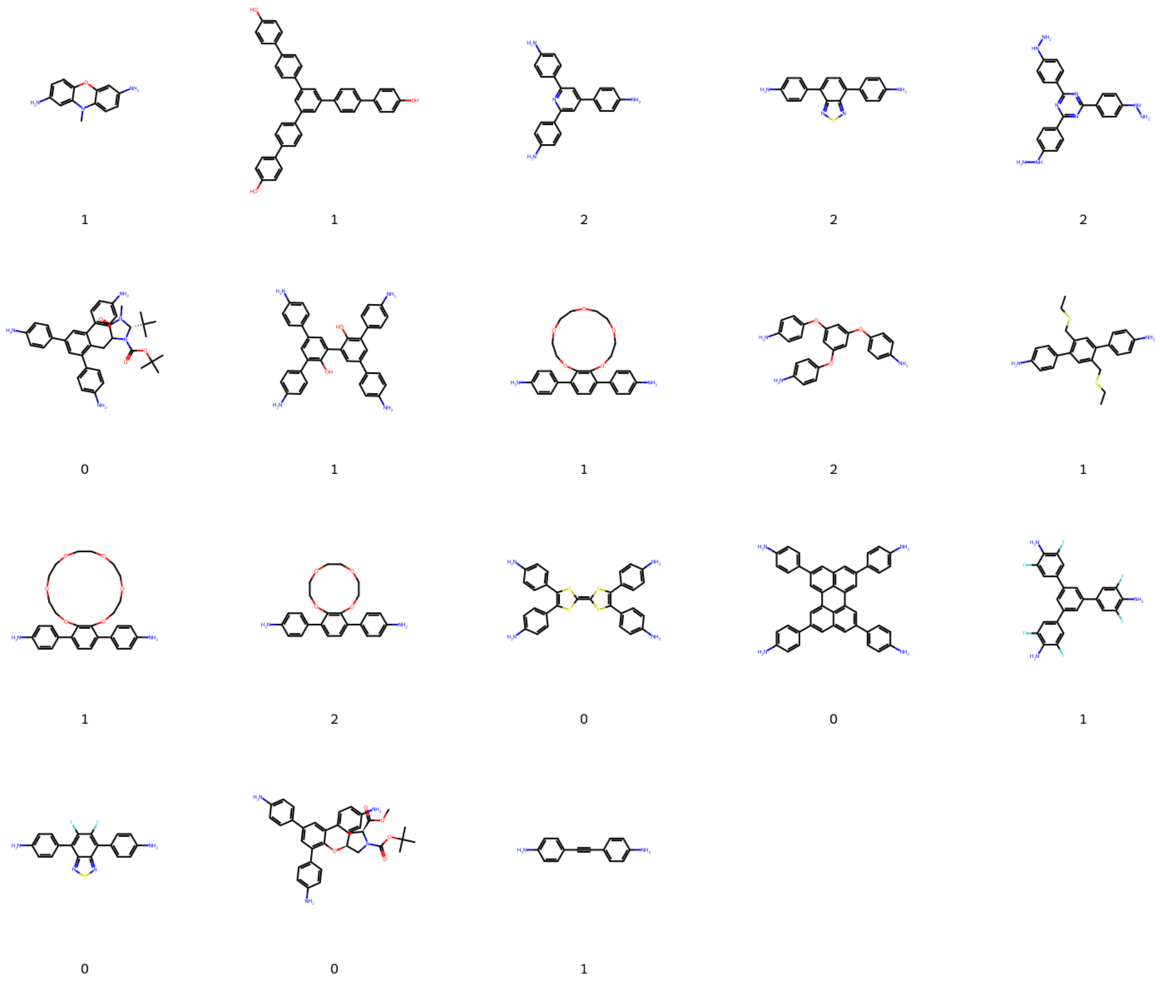

Figure S22(continued): Cluster 12

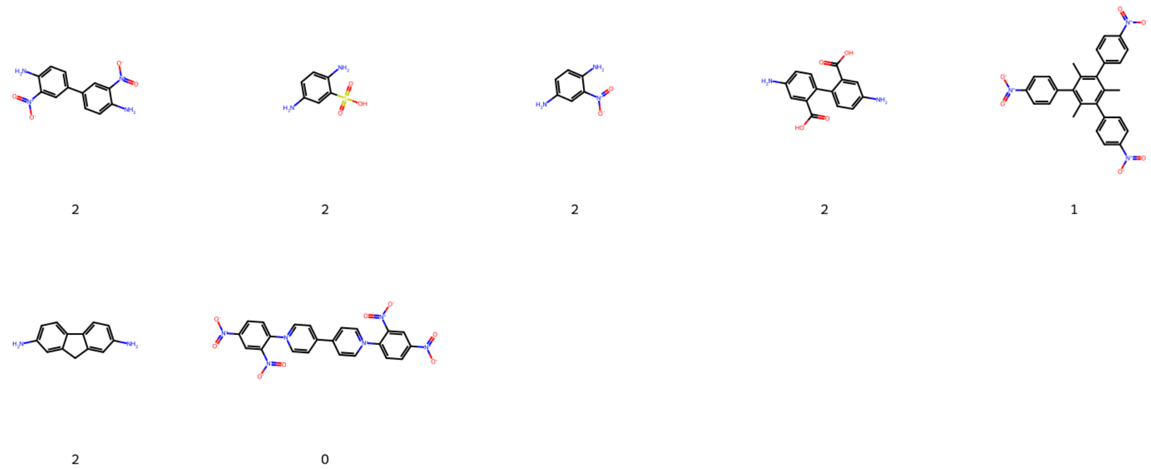

Figure S23: Cluster 13

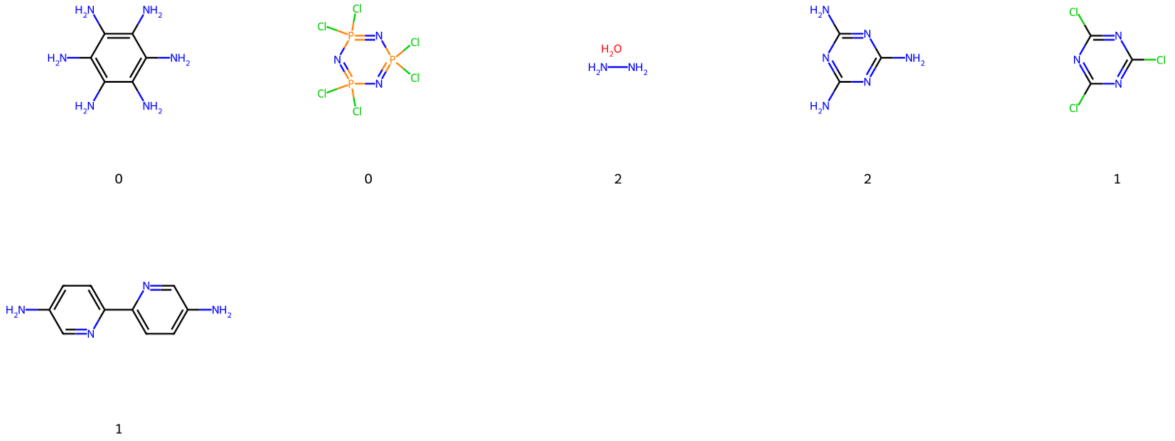

Figure S24: Cluster 14

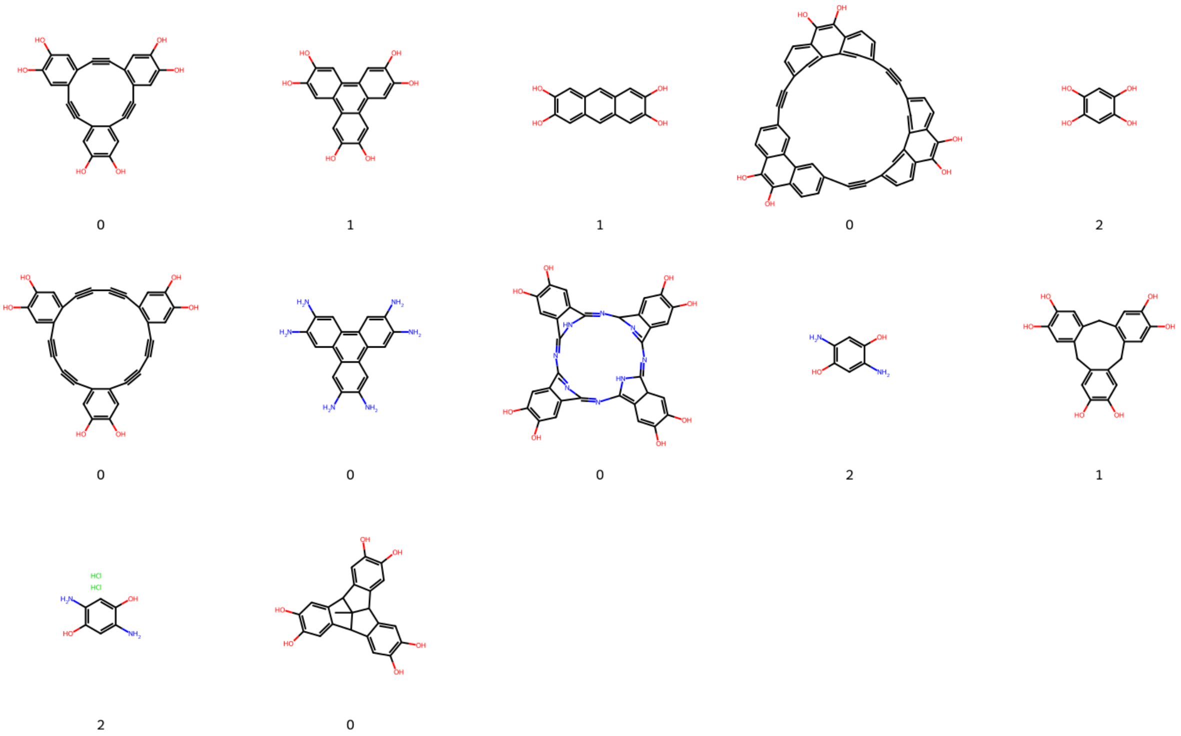

Figure S25: Cluster 15

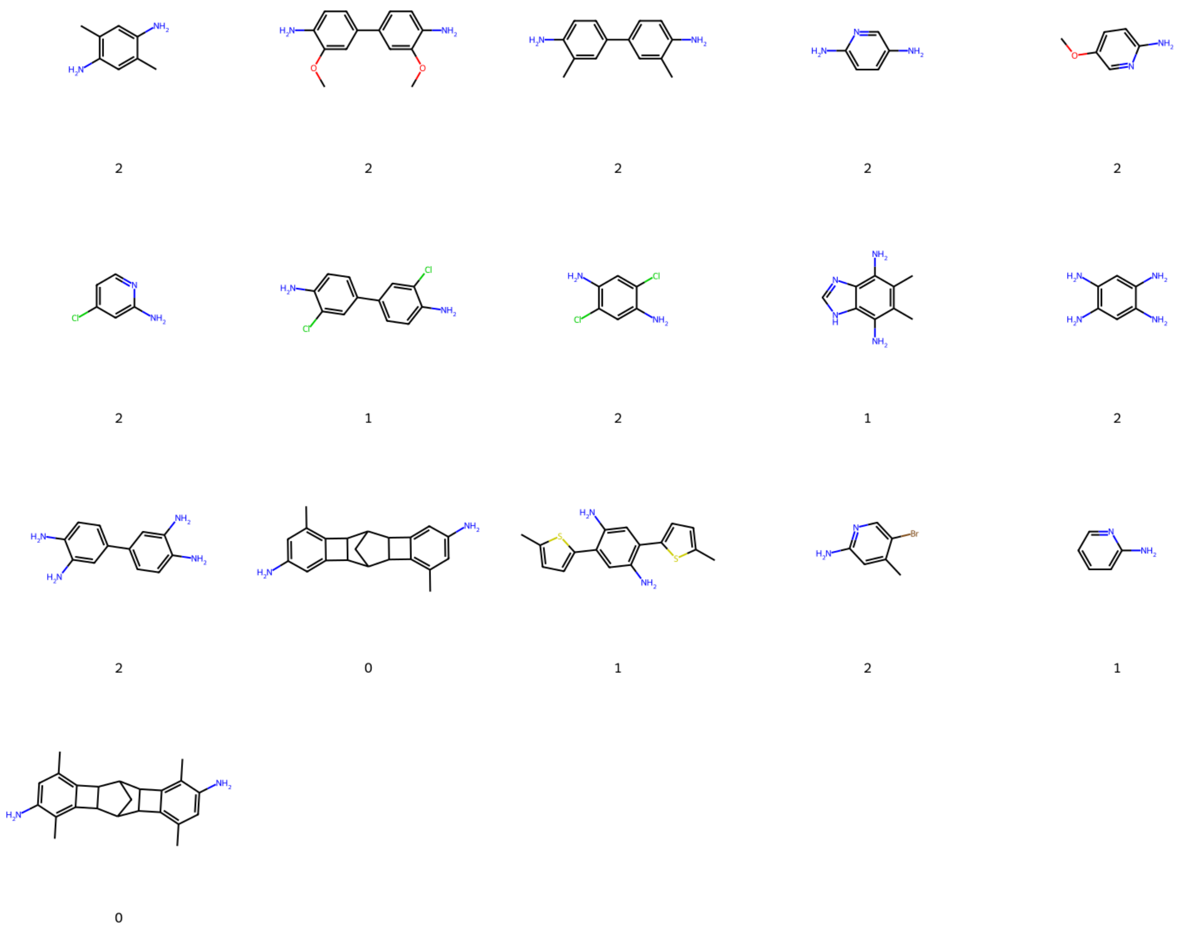

Figure S26: Cluster 16

2

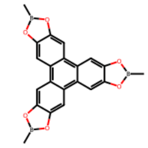

0

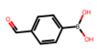

1

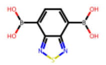

2

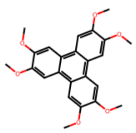

1

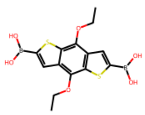

0

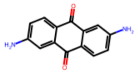

1

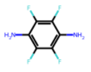

2

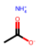

1

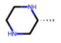

2

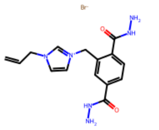

0

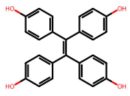

1

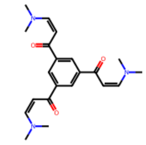

0

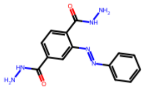

1

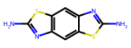

2

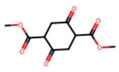

2

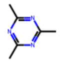

0

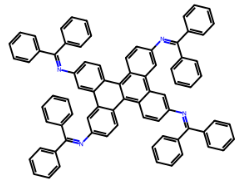

1

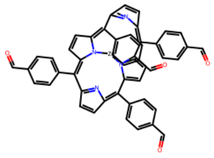

0

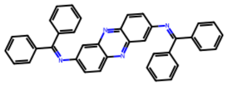

1

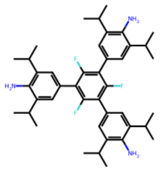

0

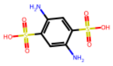

1

Figure S27: Cluster -1 (Noise)

S4 Monomers Structure Analysis by GPT-4o

In addition to manual interpretation of the dominant structural fragments within each cluster, we explored the use of the GPT-4o model for qualitative chemical structure analysis. The SMILES strings of monomers in each cluster were provided to the model together with a standardized prompt and same input format (to ensure consistency), without prior in-context learning or fine-tuning.

Interestingly, the model autonomously generated an RDKit script to compute the Murcko scaffolds of the input molecules before performing its analysis, which is consistent with the scaffold-based interpretation used in this work. Based on the extracted core structures, GPT-4o produced chemically meaningful descriptions for many clusters. For example, complex scaffolds such as 4,7-diphenyl-1H-benzimidazole in cluster 2 and common functional groups such as nitrile substituents in cluster 3 were correctly identified in the generated descriptions.

However, inconsistent or incorrect interpretations were occasionally observed, particularly for larger monomers. For instance, GPT-4o described cluster 7 as having "no fused aromatic systems or heterocycles," despite the presence of multiple monomers containing fused aromatic rings and heteroatoms. These limitations are consistent with previous evaluations of large language models for chemical reasoning, where Mirza et al.[7] reported that LLMs may struggle to interpret molecular structures represented by SMILES strings when the number of atoms becomes large.

The prompt used for GPT-4o analysis is shown below.

Prompt:

Analyze the molecules in the attached file to identify the dominant scaffold(s) and key structural features defining this cluster. Focus on major recurring motifs such as fused aromatic systems, heterocycles, or common linkers. Highlight specific substructures (e.g., indole, benzimidazole, benzaldehyde) if consistently observed. Briefly comment on the presence or absence of common functional groups (e.g., amines, carbonyls, nitriles, halogens) and whether these groups show uniformity across the cluster. Summarize the findings in a compact, narrative format, emphasizing the main structural elements and functional group patterns without excessive detail.

A python script generated autonomously by GPT-4o for structure analysis:

```python
# Re-import necessary modules after file upload
import pandas as pd
from rdkit import Chem
from rdkit.Chem.Scaffolds import MurckoScaffold
from collections import Counter

# Load the new uploaded file
file_path = "/mnt/data/cluster_0.csv"
df = pd.read_csv(file_path)
```

```python
# Function to extract Murcko scaffold
def get_murcko_scaffold(smiles):
    mol = Chem.MolFromSmiles(smiles)
    if mol:
        scaffold = MurckoScaffold.GetScaffoldForMol(mol)
        return Chem.MolToSmiles(scaffold)
    return None

# Apply scaffold extraction
df["scaffold"] = df["smiles"].apply(get_murcko_scaffold)
scaffold_counts = Counter(df["scaffold"].dropna())

# Show most common scaffolds
scaffold_counts.most_common(3)
```

Following is the response generated by GPT-4o per cluster:

- Cluster 0
  The molecules in this cluster are defined by a dominant scaffold centered on a fused polyaromatic system that resembles a substituted *1H*-indole or carbazole core. This tricyclic scaffold, often bearing multiple pendant phenyl rings, appears in over half the molecules, indicating high structural uniformity.

  Key recurring motifs include fused benzene-pyrrole systems (indole-like), extended with various phenyl or heteroaryl groups such as thiophenes. The prevalent scaffold architecture is planar and π-conjugated, forming large aromatic frameworks.

  Functionally, secondary amines (from the indole nitrogen) and aromatic carbonyl groups are common. No halogens or nitriles are observed across the cluster, and the functional groups tend to be consistent, reinforcing a narrow chemical profile.

  Overall, this cluster is structurally characterized by extended indole-like fused systems with heavy aromatic substitution and consistent incorporation of amines and carbonyl functionalities..

- Cluster 1
  The dominant structural features in this cluster are characterized by phenylacetylene-based scaffolds. The most common scaffold includes multiple ethynyl linkers (–C≡C–) connecting aromatic rings, such as phenyl or heteroaromatic units. Specifically, the leading scaffold observed in three molecules contains a fused thiazole–thiadiazole core substituted with terminal ethynyl-phenyl groups, suggesting an extended, rigid conjugated system.

  Across the cluster, ethynyl-phenyl motifs are prevalent, often appearing in symmetrical or linear arrangements. The consistent presence of triple bonds indicates a strong enrichment of sp-hybridized carbon linkers, likely introduced to rigidify the scaffold or enhance electronic delocalization.

  Functional group diversity is low: carbonyls, amines, nitriles, and halogens are mostly absent. Instead, the chemistry is dominated by carbon–carbon triple bonds and aromatic systems, suggesting a design focus on π-conjugation rather than polarity or hydrogen bonding.

  In summary, this cluster is defined by phenylacetylene scaffolds with recurring ethynyl linkers and minimal functional group variation, emphasizing linear, conjugated aromatic architectures.

- Cluster 2
  The molecules in this cluster are predominantly built around a 4,7-diphenyl-1*H*-benzimidazole core, a fused bicyclic heteroaromatic scaffold where the benzimidazole is symmetrically substituted at the 4 and 7 positions with phenyl groups. This motif, observed in multiple entries, serves as the rigid, planar backbone anchoring the molecular architecture.

Substitutions typically extend from the central benzimidazole through additional aryl groups, maintaining overall conjugation. Minor variations include incorporation of saturated side chains like pyrrolidine rings, but these are sparse.

Functionally, the structures consistently feature secondary amines from the benzimidazole nitrogen, while polar functionalities such as nitriles, carbonyls, and halogens are generally absent. This suggests a focus on aromatic π-conjugation over polar interaction motifs.

In short, this cluster is defined by the repeated presence of 4,7-diphenyl-1H-benzimidazole scaffolds with extended phenyl substitution and limited functional group diversity.

- Cluster 3
  The molecules in this cluster are primarily defined by simple aromatic systems, with benzene (phenyl) rings serving as the dominant scaffold. The most frequent structures include mono- and biphenyl motifs, as well as fused systems like naphthalene. These cores are typically unsubstituted or bear minimal functionalization.

  A common and distinguishing feature across this cluster is the presence of nitrile groups (–C≡N), frequently attached to aromatic rings. These electron-withdrawing groups are the primary functional handles observed and appear consistently across the dataset.

  Other functional groups such as amines, carbonyls, or halogens are largely absent, indicating a narrow chemical profile. The molecules are structurally simple and dominated by aromaticity with appended nitriles.

  In summary, this cluster is characterized by phenyl- and naphthyl-based scaffolds bearing nitrile substituents, with otherwise minimal structural or functional group complexity.

- Cluster 4
  This cluster is dominated by simple aromatic scaffolds, with unsubstituted phenyl (benzene) rings being the most common core. In addition to mono-phenyl structures, some molecules feature biphenyl units or symmetric trisubstituted benzene rings, often extended by additional phenyl groups.

  A recurring structural element is the presence of aryl–ethynyl linkers (–C≡C–Ar), though less frequent than in cluster 1. These linkers, when present, contribute to molecular linearity and conjugation.

  From a functional group perspective, this cluster is chemically minimal. Nitriles, carbonyls, amines, and halogens are largely absent. The molecules show high uniformity, defined by hydrophobic, π-conjugated frameworks with little polar or reactive functionality.

  In summary, cluster 4 is structurally characterized by phenyl- and biphenyl-based scaffolds, with sparse ethynyl linkages and minimal functional group diversity. The chemistry is dominated by simple aromatic units and extended π-systems.

- Cluster 5
  The molecules in this cluster are structurally diverse but unified by the presence of

complex polycyclic aromatic systems with high oxygen content. Several compounds feature highly fused ring systems with multiple embedded carbonyl groups, reminiscent of polyquinone or polyimide-like motifs.

A dominant structural theme is the presence of multiple ketone functionalities embedded within extended aromatic or heteroaromatic frameworks. These structures often include fused heterocycles, such as isoindole or naphthalimide-like cores. At least one molecule includes a triphenylene-like fused system heavily substituted with carbonyl groups and diaryl imide linkages.

Functionally, carbonyl groups—particularly ketones and imides—are strongly enriched across the cluster. Amines, nitriles, and halogens are absent, and the molecules lack aliphatic flexibility, reinforcing a rigid, planar chemical space.

In summary, this cluster is defined by fused aromatic or heteroaromatic ring systems with dense ketone and imide substitution, resulting in highly conjugated and oxygen-rich scaffolds with limited functional diversity.

- Cluster 6
  The dominant scaffold in this cluster is a fused dithienonaphthalene-like structure, composed of two thiophene rings integrated into a polycyclic aromatic system. This specific fused thiophene architecture appears consistently across several molecules, suggesting a deliberate design toward extended conjugation and electronic delocalization.

  The cluster also includes simpler thiophene derivatives and substituted bithiophenes, reinforcing the strong presence of sulfur-containing five-membered heterocycles. Aromaticity is a unifying feature, with all structures being fully conjugated and planar.

  From a functional group perspective, the molecules are minimalistic. There is a near-complete absence of polar groups such as carbonyls, nitriles, amines, or halogens, resulting in high uniformity across the dataset. This suggests the cluster is designed for electronic or optical applications where polarity is minimized.

  In summary, this cluster is defined by sulfur-rich fused thiophene systems and simple thiophene-based heterocycles, with no significant polar functionalities, yielding a chemically uniform, highly aromatic, and conjugated set of structures.

- Cluster 7
  This cluster is dominated by simple polyphenyl architectures, with triphenylbenzene and biphenyl scaffolds appearing most frequently. The leading structure features a 1,3,5-triphenylbenzene-like core where each position on a central benzene ring is substituted with a phenyl group, forming a rigid and symmetric aromatic framework.

  Most molecules consist exclusively of phenyl rings connected via direct C–C bonds, indicating a strong emphasis on π-conjugated systems. No fused aromatic systems or heterocycles are observed.

  In terms of functional groups, the cluster is uniformly non-polar. There is a complete absence of common functional groups such as carbonyls, amines, nitriles, or halogens. This lack of polarity, combined with extensive aromatic content, suggests molecules

tailored for structural rigidity and electronic delocalization rather than chemical reactivity or solubility modulation.

In summary, this cluster is defined by highly symmetric, rigid polyphenyl scaffolds like triphenylbenzene and biphenyl, with no significant functionalization beyond aromatic ring extension.

- Cluster 8
  Cluster 8 is characterized by sulfur-containing heterocycles, primarily composed of mono- and poly-thiophene systems. The key recurring motifs include thiophene (a five-membered ring with one sulfur atom), benzodithiophene (a fused system of two thiophenes with a central benzene), and extended fused thiophene-based frameworks.

  These scaffolds reflect a strong emphasis on π-conjugation and planarity, with sulfur atoms integrated into the conjugated systems to modulate electronic properties. The structures are compact and aromatic, suggesting use in organic semiconductors or related electronic materials.

  Functional group analysis shows minimal diversity. Common polar groups such as carbonyls, amines, nitriles, and halogens are absent. The molecules are uniformly nonpolar and consist almost entirely of aromatic carbon and sulfur atoms.

  In summary, this cluster is defined by simple and fused thiophene-based scaffolds, with no additional functionalization. The structures are planar, sulfur-rich, and fully aromatic, indicating a highly uniform and electronically tuned molecular set.

- Cluster 9
  Cluster 9 features a structurally diverse set of molecules with a general bias toward phenyl-based architectures. The most common scaffold is the unsubstituted phenyl ring, followed by more complex systems like triphenylmethane derivatives and aryl–ethynyl–aryl motifs.

  While aromaticity is consistent throughout the cluster, there is no single dominant polycyclic or fused heteroaromatic core. Several structures include extended conjugated systems with central sp^2- or sp-hybridized carbon linkers connecting multiple aryl units.

  In terms of functional groups, the cluster is largely non-polar. Carbonyls, amines, nitriles, and halogens are notably absent. The structures emphasize π-conjugation with minimal chemical handles, reflecting a hydrophobic, aromatic-rich space.

  In summary, cluster 9 is defined by loosely related aryl-based scaffolds, with some molecules incorporating ethynyl bridges and triaryl cores. Functional group uniformity is high, marked by a consistent absence of polar moieties.

- Cluster 10
  Cluster 10 is primarily defined by simple aryl-based structures, with the phenyl ring (benzene) being the overwhelmingly dominant scaffold. The majority of molecules consist of single phenyl rings, and a few contain more elaborate architectures such as trisubstituted benzene cores extended by additional phenyl units.

There is a complete absence of fused aromatic systems, heterocycles, or complex linkers. The structural diversity is minimal, centering almost entirely on direct aryl–aryl connectivity.

Functionally, this cluster is uniformly non-polar. No amines, carbonyls, nitriles, halogens, or other polar substituents are present. The molecules exhibit high aromatic character with no additional chemical functionality.

In summary, Cluster 10 is characterized by simple phenyl and polyphenyl scaffolds with extremely limited structural and functional variation, suggesting a highly uniform, non-polar aromatic space.

- Cluster 11
  Cluster 11 is primarily composed of simple aromatic frameworks, with the phenyl ring (benzene) being the most recurrent scaffold. Several molecules are either single phenyl rings or linked phenyl units such as biphenyls.

  One notable molecule introduces a more complex motif: a 1,3,5-trisubstituted benzene core bearing three benzamide groups, forming a tripodal scaffold with secondary amide linkages to additional phenyl rings. This structure suggests a more functionalized entry relative to the rest of the cluster.

  Overall, the majority of the molecules lack heterocycles or fused aromatic systems. Functional group analysis reveals a mostly uniform profile: the majority of the cluster lacks polar substituents, though a few molecules contain amide functionalities, contributing hydrogen-bonding capability and some polarity.

  In summary, Cluster 11 is dominated by phenyl and biphenyl scaffolds, with minimal structural complexity and functional group variation. The rare inclusion of benzamide linkers introduces limited polarity in an otherwise non-polar, aromatic-rich cluster.

- Cluster 12
  Cluster 12 is defined by extended polyphenyl scaffolds, with the dominant motif being a 1,3,5-trisubstituted benzene core connected to multiple phenyl rings. The most frequent structure is triphenylbenzene, and more complex analogs expand this pattern with further aryl substitutions, forming highly symmetric, branched aromatic frameworks.

  These molecules exhibit no fused rings or heterocycles. The scaffolds are entirely composed of sp^2 carbon atoms, emphasizing rigid, planar architectures and extended π-conjugation.

  Functionally, the cluster is uniformly non-polar. There are no amines, carbonyls, nitriles, or halogens present, and no heteroatoms appear in the dataset. The chemical space is strictly aryl-based, with minimal functional group diversity.

  In summary, Cluster 12 is characterized by highly substituted aryl scaffolds, particularly tris- and pentasubstituted benzenes with extended phenyl branches. The structures are rigid, symmetric, and purely aromatic, with no polar functionalities or heterocycles observed.

- Cluster 13
  Cluster 13 is composed primarily of simple aromatic scaffolds, with biphenyl and phenyl rings being the most recurring structures. A few molecules extend this motif into triphenylbenzene-type frameworks, forming rigid, planar, and conjugated systems.

  There are no fused ring systems or heterocycles present. The molecules exclusively feature aryl–aryl connections, typically through direct C–C bonds, without additional linkers or branching elements.

  Functionally, the cluster is entirely non-polar. No amines, carbonyls, nitriles, halogens, or other polar groups are present. The chemical space is uniform, centered around unsubstituted or symmetrically substituted phenyl rings.

  In summary, Cluster 13 is characterized by phenyl and biphenyl scaffolds with occasional triaryl extensions. The structures are fully aromatic, planar, and chemically simple, with no heteroatoms or polar functionalities.

- Cluster 14
  Cluster 14 presents a distinct structural profile compared to previous clusters, with the dominant motif being heteroaromatic and inorganic ring systems rather than simple aryl frameworks. The most frequently observed scaffold is a 1,2,4-triazine core (`c1ncncn1`), a six-membered ring containing three nitrogen atoms in alternating positions, known for its high electron deficiency and synthetic versatility.

  Additionally, the cluster includes a rare inorganic ring system, cyclotriphosphazene (N_3P_3), represented by the scaffold `N1=PN=PN=P1`, which is characteristic of phosphorus–nitrogen chemistry and often used in flame retardants or advanced materials.

  Aromatic phenyl rings are present in some molecules, but they are not the dominant motif. Functionally, the cluster contains several nitrogen-rich scaffolds, with heteroatoms incorporated directly into the core structures. However, there is little evidence of conventional functional groups like carbonyls, amines, nitriles, or halogens.

  In summary, Cluster 14 is defined by heterocyclic and inorganic ring systems, particularly 1,2,4-triazine and cyclotriphosphazene. The structures are electron-deficient and nitrogen-rich, setting this cluster apart from the predominantly aryl-based motifs of other groups.

- Cluster 15
  Cluster 15 includes a combination of simple phenyl rings and more complex polycyclic aromatic hydrocarbons. The most frequent scaffold is the phenyl ring, though it is closely followed by a fused tricyclic system resembling triphenylene or anthracene, composed of three linearly or angularly fused benzene rings.

  Another notable structure includes multiple phenylacetylene units, with triple bonds (–C≡C–) linking aromatic rings. This suggests an interest in extended π-conjugation and rigid, linear molecular architectures.

  No heterocycles or heteroatoms are observed, and common polar functional groups such as carbonyls, amines, nitriles, or halogens are absent. The cluster is uniformly non-polar and composed entirely of carbon-based aromatic and alkyne systems.

In summary, Cluster 15 is defined by phenyl, fused polycyclic aromatic hydrocarbons, and aryl–ethynyl linkers. The molecular structures emphasize planarity and π-conjugation with no polar functionalization, suggesting applications in organic electronics or materials chemistry.

- Cluster 16

  Cluster 16 is primarily defined by simple aromatic and heteroaromatic scaffolds, with pyridine (`c1ccncc1`) as the most common heterocycle. Phenyl and biphenyl units are also prominent, either isolated or appended to heterocycles.

  The pyridine ring introduces nitrogen as the only recurring heteroatom in this cluster, providing potential coordination or hydrogen-bonding capability. Despite this, the cluster remains largely non-polar, as no other functional groups—such as carbonyls, amines, nitriles, or halogens—are observed in the dominant motifs.

  There are no fused aromatic systems or complex linker groups present. The molecular structures favor linear or planar arrangements involving C–C and C–N aromatic connectivity.

  In summary, Cluster 16 is characterized by pyridine and phenyl-based scaffolds with simple aryl–aryl or aryl–heteroaryl connections. The functional group landscape is minimal and consistent, dominated by aromatic nitrogen heterocycles with no additional polar functionalities.

S5 Parameter Setting and Results of Retrosynthesis Analysis

The scripts (or configuration) applied in the two retrosynthesis tools for analyzing monomer synthetic routes are available in the SI folder (`retro_star_query.py` for ASKCOSv2 and `config_original.yaml` for AiZynthFinder) for reference. Both shared the same input file consisting of the 359 unique monomers `Unique_359.txt`.

Table S3 lists the index of all 359 unique monomers extracted from existing COFs. The `Frequency` column indicates how many times each monomer appeared in the sourced literature. The `Flag` column shows the status of the retrosynthesis analysis (2 = solved by both ASKCOS and AiZynthFinder; 1 = solved by either tool; 0 = no solution). The `AZF` and `ASKCOS` columns: 1 means the tool provided a solution, and 0 means no solution was found. All retrosynthesis output files are named using the corresponding monomer index. The predicted synthetic routes generated by ASKCOS and AiZynthFinder are provided in folder `Unique_359_ASKCOSv2` and `Unique_359_AiZynthFinder`.

Tables S4, S5, and S6 list the 333 restored monomers used in the construction of the 69,840 hypothetical COFs. The IUPAC names, exported from the PubChem database, are provided when available. The `Solution` column indicates which tool successfully predicted a synthetic route for each fictitious molecule (those without an IUPAC name), or whether the molecule remains unsolved. Retrosynthesis results for the 109 fictitious monomers are also provided in `Virtual_109_ASKCOSv2` and `Virtual_109_AiZynthFinder`.

S5.1 Additional Case Study for Solved/Unsolved Monomers

S5.1.1 Solved Monomer

1,3,5-tris(4-formylphenyl)benzene (mol ID 55) is a widely used building block, applied in the synthesis of more than 20 COFs.The synthetic routes predicted by both retrosynthesis tools differ from that reported in the literature (Figure S28). For COF-43[9], Uribe-Romo et al. first synthesized mol-55 by oxidizing 55a (1,3,5-tris(4-bromomethylphenyl)benzene) using tetrabutylammonium dichromate (TBADC) in chloroform[8]. ASKCOS proposes synthesizing mol-55 via a Suzuki–Miyaura cross-coupling between 55b and 55c. In contrast, AiZynthFinder predicts a route involving three successive Suzuki–Miyaura couplings using 55d and 55e as starting materials.

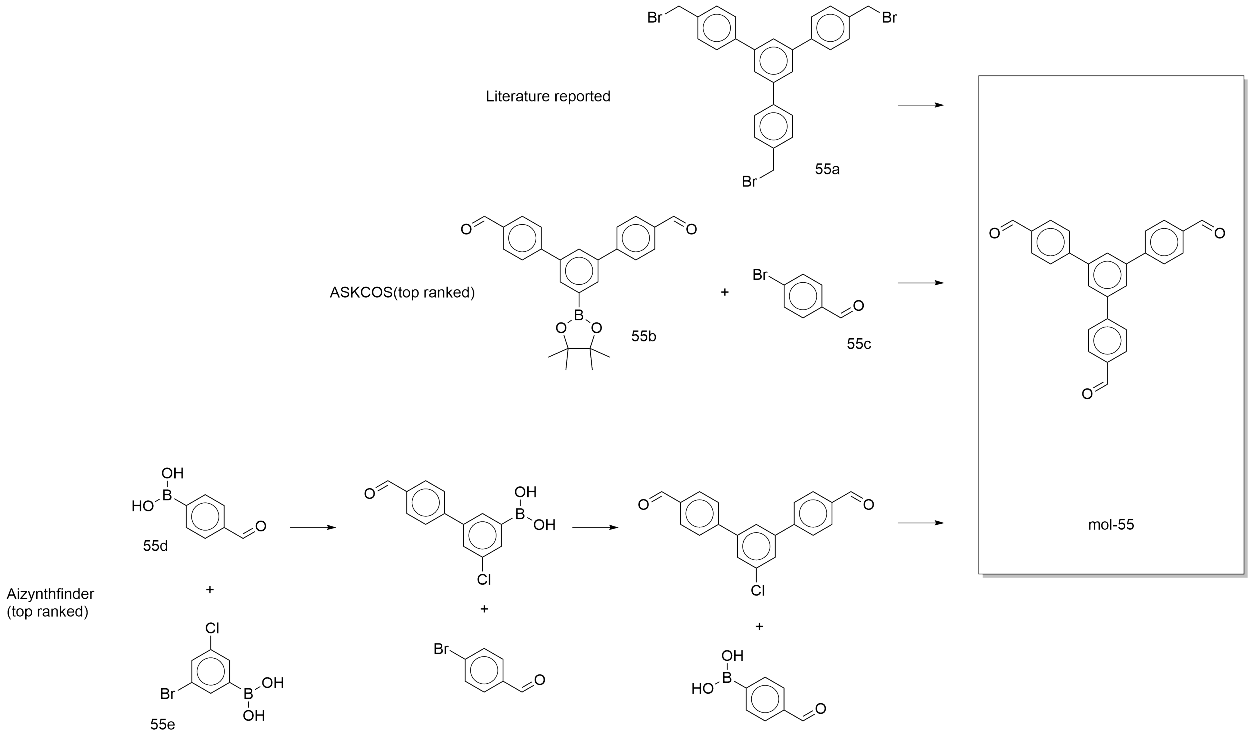

Figure S28: Synthetic route predictions for 1,3,5-tris(4-formylphenyl)benzene (mol ID 55) by ASKCOS and AiZynthFinder, compared with the literature-reported route.

S5.1.2 Unsolved Monomer

Another unsolved monomer from cluster 4, BINOL-DA (mol ID 231), which serves as the primary vertex in CCOF-7 and CCOF-8 reported by Wu et al.[11] As shown in Figure S29, the monomer was synthesized by the authors from (R)-DCDB (231b), following the procedure described by Valente et al.[10], where the synthesis of (R)-DCDB started from the molecule 1,1'-bi-2-naphthol (BINOL, 231a), originally reported by Lee and Lin[4]. ASKCOS partially captured part of the literature reported synthetic sequence, specifically identifying the step in which the aldehyde group is introduced via bromide substitution in the presence of DMF. However, the route still failed to complete because 231c was not recognized as a commercially available molecule and ASKCOS could not simplify it further ; AiZynthFinder met with the same issue.

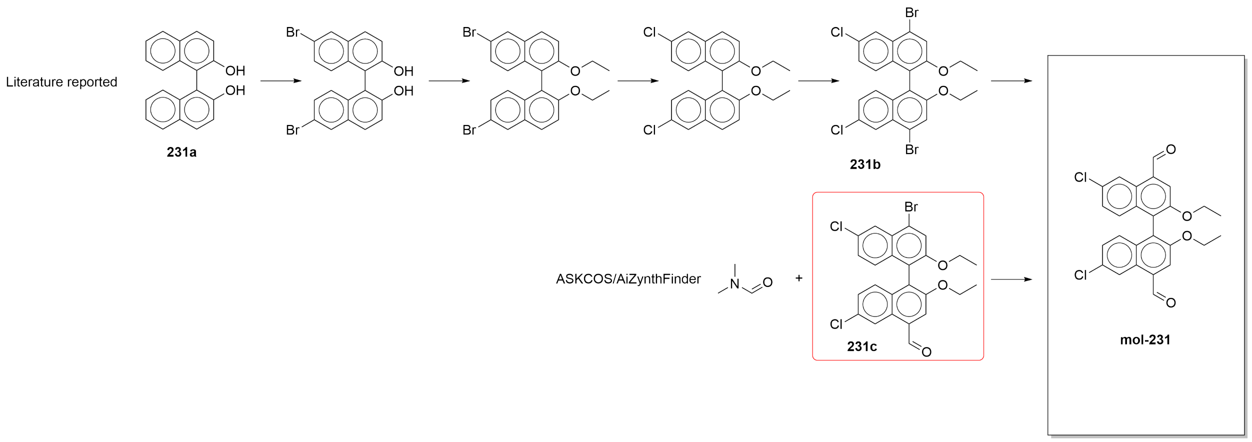

Figure S29: Unsolved synthetic route predictions for BINOL-DA (mol ID 231) by ASKCOS and AiZynthFinder, compared with the literature-reported route. Molecules blocked in red stands for starting molecules not available in stock libraries.

Table S3: List of the 359 unique monomers and index

Index	SMILES	Frequency	Flag	AZF	ASKCOS
0	O=Cc1c(O)c(C=O)c(O)c(C=O)c1O	40	2	1	1
1	Nc1ccc2cc3cc(N)ccc3cc2c1	4	2	1	1
2	CCn1c2cc(N)ccc2c2c1c1c3ccc(N)cc3n(CC)c1c1c3ccc(N)cc3n(CC)c21	1	0	0	0
3	OB(O)c1ccc(C(=C(c2ccc(B(O)O)cc2)c2ccc(B(O)O)cc2)c2ccc(B(O)O)cc2)cc1	1	1	0	1
4	Nc1ccc(-c2ccc(N)c([N+](=O)[O-])c2)cc1[N+](=O)[O-]	3	2	1	1
5	NN	8	2	1	1
6	OB(O)c1cc(B(O)O)cc(B(O)O)c1	3	2	1	1
7	Nc1ccc(/C=C/c2ccc(N)cc2)cc1	2	2	1	1
8	Nc1ccc(N)cc1	33	2	1	1
9	O=Cc1cc(C=O)cc(C=O)c1	32	2	1	1
10	Oc1cc2c(cc1O)C#Cc1cc(O)c(O)cc1C#Cc1cc(O)c(O)cc1C#C2	3	0	0	0
11	O=Cc1ccc(-c2nc(-c3ccc(C=O)cc3)nc(-c3ccc(C=O)cc3)n2)cc1	10	2	1	1
12	Oc1cc2c3cc(O)c(O)cc3c3cc(O)c(O)cc3c2cc1O	13	1	0	1
13	N#Cc1ccc(C#N)s1	1	2	1	1
14	Oc1cc2cc3cc(O)c(O)cc3cc2cc1O	2	1	0	1
15	Nc1c(N)c(N)c(N)c(N)c1N	1	0	0	0
16	O=Cc1ccc(C=O)c(O)c1O	5	2	1	1
17	O=Cc1ccc(-c2cc(-c3ccc(C=O)cc3)c3ccc4c(-c5ccc(C=O)cc5)cc(-c5ccc(C=O)cc5)c5ccc2c3c54)cc1	13	0	0	0
18	OB(O)c1ccc(C#Cc2ccc(C#Cc3ccc(B(O)O)cc3)cc2)cc1	1	2	1	1
19	N#Cc1ccc(C(=C(c2ccc(C#N)cc2)c2ccc(C#N)cc2)c2ccc(C#N)cc2)cc1	1	1	0	1
20	Nc1ccc(-c2c3nc(c(-c4ccc(N)cc4)c4ccc([nH]4)c(-c4ccc(N)cc4)c4nc(c(-c5ccc(N)cc5)c5ccc2[nH]5)C=C4)C=C3)cc1	17	1	0	1
21	Cc1cc(N)c(C)cc1N	5	2	1	1
22	Oc1c(O)c2ccc3c#cc4ccc5c(O)c(O)c6ccc(c#cc7ccc8c(O)c(O)c9ccc(c#cc%10ccc1c(c%10)c2c3)cc9c8c7)cc6c5c4	1	0	0	0
23	OB(O)c1ccc(-c2ccc(B(O)O)cc2)cc1	2	2	1	1
24	OB(O)c1cc2ccc3cc(B(O)O)cc4ccc(c1)c2c34	4	2	1	1
25	CCOc1cc(C(=O)NN)c(OCC)cc1C(=O)NN	4	1	0	1
26	Nc1ccc(-c2c(-c3ccc(N)cc3)c(-c3ccc(N)cc3)c(-c3ccc(N)cc3)c(-c3ccc(N)cc3)c2-c2ccc(N)cc2)cc1	3	1	0	1
27	ClP1(Cl)=NP(Cl)(Cl)=NP(Cl)(Cl)=N1	1	0	0	0
28	OB(O)c1ccc(/N=N/c2ccc(B(O)O)cc2)cc1	1	1	0	1
29	O=Cc1c(F)c(F)c(C=O)c(F)c1F	4	2	1	1

Table S3 (continued): List of the 359 unique monomers and index

Index	SMILES	Frequency	Flag	AZF	ASKCOS
30	C1=Cc2nc1c(-c1ccccc1)c1ccc([nH]1)c(-c1ccccc1)c1nc(c(-c3ccccc3)c3ccc([nH]3)c2-c2ccccc2)C=C1	1	1	0	1
31	CC1(C)OC(C(O)(c2ccc(-c3ccc(C=O)cc3)cc2)c2ccc(-c3ccc(C=O)cc3)cc2)C(C(O)(c2ccc(-c3ccc(C=O)cc3)cc2)c2ccc(-c3ccc(C=O)cc3)cc2)O1	1	0	0	0
32	CC(C)(C)[Si](C)(C)ON(C(=O)OCC[Si](C)(C)C)c1ccc(-c2c3nc(c(-c4ccc(N(O[Si](C)(C)C(C)(C)C)C(=O)OCC[Si](C)(C)C)cc4)c4ccc([nH]4)c(-c4ccc(N(O[Si](C)(C)C(C)(C)C)C(=O)OCC[Si](C)(C)C)cc4)c4nc(c(-c5ccc(N(O[Si](C)(C)C(C)(C)C)C(=O)OCC[Si](C)(C)C)cc5)c5ccc2[nH]5)C=C4)C=C3)cc1	1	0	0	0
33	N#Cc1ccc(C#N)cc1	3	2	1	1
34	Nc1ccc(-c2cc(-c3ccc(N)cc3)cc(-c3ccc(N)cc3)c2)cc1	36	2	1	1
35	Nc1ccc(-c2nc(-c3ccc(N)cc3)nc(-c3ccc(N)cc3)n2)cc1	24	2	1	1
36	Nc1ccc(-c2ccc(N)cc2)cc1	12	2	1	1
37	O=Cc1cc(C=O)cc(-c2cc(C=O)cc(C=O)c2)c1	1	2	1	1
38	O=Cc1ccc(N(c2ccc(C=O)cc2)c2ccc(-c3ccc(N(c4ccc(C=O)cc4)c4ccc(C=O)cc4)cc3)cc2)cc1	1	2	1	1
39	O=Cc1ccc(C=O)cc1	28	2	1	1
40	COc1cc(C(=O)NN)c(OC)cc1C(=O)NN	3	2	1	1
41	Nc1ccc(Cc2ccc(N)cc2)cc1	2	2	1	1
42	O=Cc1cc(O)c(C=O)cc1O	15	2	1	1
43	OB(O)c1ccc(C#CC#Cc2ccc(B(O)O)cc2)cc1	2	1	0	1
44	Oc1cc(O)c(O)cc1O	2	2	1	1
45	OB(O)c1ccc(B(O)O)cc1	5	2	1	1
46	N#Cc1ccc(-c2cc(-c3ccc(C#N)cc3)cc(-c3ccc(C#N)cc3)c2)cc1	1	2	1	1
47	O=C1c2ccc3c4ccc5c6c(ccc(c7ccc(c2c37)C(=O)N1c1ccc(B(O)O)cc1)c64)C(=O)N(c1ccc(B(O)O)cc1)C5=O	1	1	0	1
48	O=Cc1ccc(-c2c3nc(c(-c4ccc(C=O)cc4)c4ccc([nH]4)c(-c4ccc(C=O)cc4)c4nc(c(-c5ccc(C=O)cc5)c5ccc2[nH]5)C=C4)C=C3)cc1	2	0	0	0
49	CB1Oc2cc3c4cc5c(cc4c4cc6c(cc4c3cc2O1)OB(C)O6)OB(C)O5	1	0	0	0
50	NN.O	3	2	1	1
51	COc1cc2c(cc1C=O)Cc1cc(OC)c(C=O)cc1Cc1cc(OC)c(C=O)cc1C2	1	1	0	1
52	C#CCOc1cc(C=O)c(OCC#C)cc1C=O	3	2	1	1
53	O=Cc1ccc(-c2cc(-c3ccc(C=O)cc3)nc(-c3ccc(C=O)cc3)n2)cc1	1	2	1	1
54	OB(O)c1cc2sc(B(O)O)cc2s1	2	2	1	1

Table S3 (continued): List of the 359 unique monomers and index

Index	SMILES	Frequency	Flag	AZF	ASKCOS
55	O=Cc1ccc(-c2cc(-c3ccc(C=O)cc3)cc(-c3ccc(C=O)cc3)c2)cc1	20	2	1	1
56	Oc1cc2c#cc#cc3cc(O)c(O)cc3c#cc#cc3cc(O)c(O)cc3c#cc#cc2cc1O	2	0	0	0
57	Nc1ccc(-c2cc3c4cc(-c5ccc(N)cc5)cc5c6cc(-c7ccc(N)cc7)cc7c8cc(-c9ccc(N)cc9)cc9c%10cc(-c%11ccc(N)cc%11)cc%11c%12cc(-c%13ccc(N)cc%13)cc%13c(c2)c3c2c(c45)c(c67)c(c89)c(c%11%10)c2c%13%12)cc1	1	0	0	0
58	O=Cc1ccc(B(O)O)cc1	1	1	1	0
59	O=Cc1ccc(-c2cc(-c3ccc(C=O)cc3)nc(-c3ccc(C=O)cc3)c2)cc1	1	2	1	1
60	Nc1ccc(/N=N/c2ccc(N)cc2)cc1	3	2	1	1
61	NNC(=O)c1ccc(-c2c3nc(c(-c4ccc(C(=O)NN)cc4)c4ccc([nH]4)c(-c4ccc(C(=O)NN)cc4)c4nc(c(-c5ccc(C(=O)NN)cc5)c5ccc2[nH]5)C=C4)C=C3)cc1	1	0	0	0
62	OB(O)c1ccc(-c2c3nc(cc4ccc([nH]4)c(-c4ccc(B(O)O)cc4)c4nc(cc5ccc2[nH]5)C=C4)C=C3)cc1	1	0	0	0
63	O=Cc1ccc(-c2cc(C=O)cc(C=O)c2)cc1	1	2	1	1
64	CCn1c2cc(C=O)ccc2c2c1c1c3ccc(C=O)cc3n(CC)c1c1c3ccc(C=O)cc3n(CC)c21	1	0	0	0
65	OB(O)c1ccc(-c2c3nc(c(-c4ccc(B(O)O)cc4)c4ccc([nH]4)c(-c4ccc(B(O)O)cc4)c4nc(c(-c5ccc(B(O)O)cc5)c5ccc2[nH]5)C=C4)C=C3)cc1	1	0	0	0
66	Nc1ccc(N)c(S(=O)(=O)O)c1	2	2	1	1
67	Nc1ccc(N)c([N+](=O)[O-])c1	2	2	1	1
68	Nn1c(=O)c2cc3c(=O)n(N)c(=O)c3cc2c1=O	1	1	0	1
69	O=Cc1ccc(Oc2nc(Oc3ccc(C=O)cc3)nc(Oc3ccc(C=O)cc3)n2)cc1	5	2	1	1
70	O=C1C(=O)C(=O)C(=O)C(=O)C1=O	1	0	0	0
71	CC(C)(C)c1cc2c3c(c1)c(=O)c(=O)c1cc(C(C)(C)C)cc(c1-3)c(=O)c2=O	3	0	0	0
72	N#Cc1c(F)c(F)c(C#N)c(F)c1F	2	2	1	1
73	O=Cc1ccc(C#Cc2ccc(C#Cc3ccc(C=O)cc3)c3nsnc23)cc1	1	2	1	1
74	Nc1ccc(Oc2nc(Oc3ccc(N)cc3)nc(Oc3ccc(N)cc3)n2)cc1	1	2	1	1
75	OB(O)c1ccc(B(O)O)c2nsnc12	2	2	1	1
76	N#Cc1cc(C#N)cc(C#N)c1	1	2	1	1
77	Nc1ccc(-c2cc(-c3ccc(N)cc3)c3ccc4c(-c5ccc(N)cc5)cc(-c5ccc(N)cc5)c5ccc2c3c54)cc1	9	1	0	1
78	COc1c(C=O)ccc(C=O)c1OC	3	2	1	1
79	OB(O)c1ccc(B(O)O)s1	1	2	1	1
80	Nc1cc2c3cc(N)c(N)cc3c3cc(N)c(N)cc3c2cc1N	1	0	0	0
81	COc1cc(-c2ccc(N)c(OC)c2)ccc1N	4	2	1	1
82	O=c1c(O)c(O)c1=O	2	1	1	0

Table S3 (continued): List of the 359 unique monomers and index

Index	SMILES	Frequency	Flag	AZF	ASKCOS
83	CC1(C)OB(c2ccc(N3C(=O)c4ccc5c6c(ccc(c46)C3=O)C(=O)N(c3ccc(B4OC(C)(C)C(C)(C)O4)cc3)C5=O)cc2)OC1(C)C	1	1	0	1
84	COc1cc2c3cc(OC)c(OC)cc3c3cc(OC)c(OC)cc3c2cc1OC	1	1	0	1
85	Nc1ccc(C(=C(c2ccc(N)cc2)c2ccc(N)cc2)c2ccc(N)cc2)cc1	11	1	0	1
86	N#Cc1ccc(-c2ccc(C#N)cc2)cc1	1	2	1	1
87	N#Cc1ccc2cc(C#N)ccc2c1	1	2	1	1
88	OB(O)c1cc2cc3sc(B(O)O)cc3cc2s1	2	1	0	1
89	Nc1ccc(N)c2nsnc12	1	2	1	1
90	CC1(C)OB(c2ccc(-n3c(=O)c4cc5c(=O)n(-c6ccc(B7OC(C)(C)C(C)(C)O7)cc6)c(=O)c5cc4c3=O)cc2)OC1(C)C	1	1	0	1
91	OC1=CC2=C3/N=C4\N=C(/N=c5\[nH]/c(c6cc(O)c(O)cc56)=N\C5N=C(/N=C(\N3)C2C=C1O)c1cc(O)c(O)cc15)c1cc(O)c(O)cc14	1	0	0	0
92	O=Cc1ccc(C2=C(c3ccc(C=O)cc3)SC(=C3SC(c4ccc(C=O)cc4)=C(c4ccc(C=O)cc4)S3)S2)cc1	4	0	0	0
93	C1=Cc2nc1c(-c1ccc(-c3cccs3)cc1)c1ccc([nH]1)c(-c1ccc(-c3cccs3)cc1)c1nc(c(-c3ccc(-c4cccs4)cc3)c3ccc([nH]3)c2-c2ccc(-c3cccs3)cc2)C=C1	1	0	0	0
94	O=Cc1ccc(-c2ccc(C=O)nc2)cn1	1	2	1	1
95	O=C1OC(=O)c2ccc3c4ccc5c6c(ccc(c7ccc1c2c73)c64)C(=O)OC5=O	1	1	0	1
96	Nc1cc(O)c(N)cc1O	3	2	1	1
97	Cc1cc(-c2ccc(N)c(C)c2)ccc1N	5	2	1	1
98	N#Cc1ccc(C#N)c2ccccc12	1	2	1	1
99	O=Cc1ccc(C#Cc2ccc(C=O)cc2)cc1	1	2	1	1
100	Nc1ccc(-c2ccc(-c3ccc(N)cc3)c3[nH]c(C4CCCN4)nc23)cc1	1	1	0	1
101	Nc1ccc(-c2nc3c4nc(-c5ccc(N)cc5)c(-c5ccc(N)cc5)nc4c4nc(-c5ccc(N)cc5)c(-c5ccc(N)cc5)nc4c3nc2-c2ccc(N)cc2)cc1	1	0	0	0
102	CC(C)(C)c1cc(-c2cc(-c3cc(C=O)c(O)c(C(C)(C)C)c3)cc(-c3cc(C=O)c(O)c(C(C)(C)C)c3)c2)cc(C=O)c1O	1	1	0	1
103	N#Cc1cccc(C#N)n1	1	2	1	1
104	CCSCCCOc1cc(C(=O)NN)c(OCCCSCC)cc1C(=O)NN	1	2	1	1
105	OB(O)c1ccc2cc(B(O)O)ccc2c1	1	1	0	1
106	O=Cc1ccc(-c2ccc(C=O)cn2)nc1	2	2	1	1
107	CCOc1c2cc(B(O)O)sc2c(OCC)c2cc(B(O)O)sc12	1	0	0	0
108	OB(O)c1ccc(-c2ccc(B(O)O)s2)s1	1	1	1	0
109	Nc1ccc2c(c1)C(=O)c1ccc(N)cc1C2=O	2	1	0	1

Table S3 (continued): List of the 359 unique monomers and index

**Index**	**SMILES**	**Frequency**	**Flag**	**AZF**	**ASKCOS**
110	`OB(O)c1ccc(-c2cc(-c3ccc(B(O)O)cc3)cc(-c3ccc(B(O)O)cc3)c2)cc1`	1	1	0	1
111	`CC(C)c1cc(-c2cc(-c3cc(C(C)C)c(N)c(C(C)C)c3)cc(-c3cc(C(C)C)c(N)c(C(C)C)c3)c2)cc(C(C)C)c1N`	3	1	0	1
112	`O=Cc1ccc(C#Cc2cc(C=O)cc(C=O)c2)cc1`	1	2	1	1
113	`NN1C(=O)c2ccc3c4c(ccc(c24)C1=O)C(=O)N(N)C3=O`	1	0	0	0
114	`Oc1cc2c(cc1O)Cc1cc(O)c(O)cc1Cc1cc(O)c(O)cc1C2`	1	1	0	1
115	`O=Cc1ccc(-c2nc3c(-c4ccc(C=O)cc4)ccc(-c4ccc(C=O)cc4)c3[nH]2)cc1`	1	2	1	1
116	`Nc1ccc(N)nc1`	1	2	1	1
117	`Nc1c(F)c(F)c(N)c(F)c1F`	2	2	1	1
118	`COc1cc(C#Cc2ccc(C=O)cc2)c(OC)cc1C#Cc1ccc(C=O)cc1`	1	2	1	1
119	`N#C/C=C/C#N`	1	2	1	1
120	`Brc1ccc(-c2cc(-c3ccc(Br)cc3)cc(-c3ccc(Br)cc3)c2)cc1`	1	1	0	1
121	`OB(O)c1ccc(C#Cc2ccc(B(O)O)cc2)cc1`	1	2	1	1
122	`O=Cc1cc(C=O)cc(-c2ccc(N(c3ccc(-c4cc(C=O)cc(C=O)c4)cc3)c3ccc(-c4cc(C=O)cc(C=O)c4)cc3)cc2)c1`	1	1	0	1
123	`CC1(C)OC(C(O)(c2ccc(C=O)cc2)c2ccc(C=O)cc2)C(C(O)(c2ccc(C=O)cc2)c2ccc(C=O)cc2)O1`	1	0	0	0
124	`O=c1oc(=O)c2cc3c(=O)oc(=O)c3cc12`	5	1	0	1
125	`O=Cc1cc(Br)c(C=O)cc1Br`	2	2	1	1
126	`O=Cc1ccc(C#CC2C(F)=C(F)C(C#Cc3ccc(C=O)cc3)c3nsnc32)cc1`	1	0	0	0
127	`O=Cc1ccc(C#CC2C=CC(C#Cc3ccc(C=O)cc3)c3nsnc32)cc1`	1	0	0	0
128	`Nc1ccc(N(c2ccc(N)cc2)c2ccc(N)cc2)cc1`	9	2	1	1
129	`O=Cc1ccc(C#CC2C=C(F)C(C#Cc3ccc(C=O)cc3)c3nsnc32)cc1`	1	0	0	0
130	`CSc1cc(C=O)c(SC)cc1C=O`	1	2	1	1
131	`O=Cc1ccc(-c2c(-c3ccc(C=O)cc3)c(-c3ccc(C=O)cc3)c(-c3ccc(C=O)cc3)c(-c3ccc(C=O)cc3)c2-c2ccc(C=O)cc2)cc1`	1	1	0	1
132	`CC(=O)[O-].[NH4+]`	1	1	1	0
133	`Nc1ccc(C23CC4CC(c5ccc(N)cc5)(C2)CC(c2ccc(N)cc2)(C4)C3)cc1`	1	1	0	1
134	`O=Cc1ccc(C#CC#Cc2ccc(C=O)cc2)cc1`	1	2	1	1
135	`CC1(C)COC(c2ccc(-c3cc(-c4ccc(C5OCC(C)(C)CO5)cc4)cc(-c4cc(-c5ccc(N)cc5)cc(-c5ccc(N)cc5)c4)c3)cc2)OC1`	1	1	0	1
136	`Nc1nc(N)nc(N)n1`	2	2	1	1
137	`Nc1ccc(-c2c3nc(c(-c4ccc(N)cc4)c4ccc([nH]4)c4ccc([nH]4)c(-c4ccc(N)cc4)c4ccc2[nH]4)C=C3)cc1`	1	0	0	0

Table S3 (continued): List of the 359 unique monomers and index

Index	SMILES	Frequency	Flag	AZF	ASKCOS
138	O=Cc1ccc(-c2cc(-c3ccc(C=O)cc3)cc(-c3ccc(-c4cc(-c5ccc(C=O)cc5)cc(-c5ccc(C=O)cc5)c4)cc3)c2)cc1	1	1	0	1
139	Nc1ccc(/C2=C3\C=CC(=N3)/C(c3ccc(N)cc3)=c3/cc/c4n3[Zn]n3c2ccc3/C(c2ccc(N)cc2)=C2/C=CC(=N2)/C=4c2ccc(N)cc2)cc1	2	0	0	0
140	N#CCc1cc(CC#N)cc(CC#N)c1	2	2	1	1
141	O=Cc1ccc(-c2cc(-c3ccc(C=O)cc3)c(-c3ccc(C=O)cc3)cc2-c2ccc(C=O)cc2)cc1	1	1	0	1
142	O=Cc1ccc(C#Cc2ccc(C=O)c(O)c2)cc1O	1	2	1	1
143	O=Cc1ccc(-c2ccc(C=O)cc2)cc1	9	2	1	1
144	O=Cc1cc(C=O)c(O)c(C=O)c1	3	2	1	1
145	Cl.Cl.Nc1cc(O)c(N)cc1O	1	2	1	1
146	Oc1ccc(-c2cc(-c3ccc(O)cc3)cc(-c3ccc(O)cc3)c2)cc1	1	2	1	1
147	O=Cc1ccc(C=O)c2c1OCO2	1	2	1	1
148	O=Cc1ccc(/C=C/c2nc(/C=C/c3ccc(C=O)cc3)nc(/C=C/c3ccc(C=O)cc3)n2)cc1	1	1	0	1
149	O=Cc1ccc(C(c2ccc(C=O)cc2)=c2c3cc4ccccc4cc3c(=C(c3ccc(C=O)cc3)c3ccc(C=O)cc3)c3cc4ccccc4cc23)cc1	1	0	0	0
150	O=Cc1cc2ccc3cc(C=O)cc4ccc(c1)c2c34	2	1	0	1
151	NNC(=O)c1ccc(C(=O)NN)cc1	3	2	1	1
152	NNC(=O)c1cc(C(=O)NN)cc(C(=O)NN)c1	1	2	1	1
153	Nc1ccc(C#Cc2cc(C#Cc3ccc(N)cc3)c(C#Cc3ccc(N)cc3)cc2C#Cc2ccc(N)cc2)cc1	1	1	0	1
154	CC12C3c4cc(O)c(O)cc4C1c1cc(O)c(O)cc1C2c1cc(O)c(O)cc13	1	0	0	0
155	N#CCc1ccc(CC#N)cc1	4	2	1	1
156	O=Cc1ccc(-c2ccc(-c3ccc(C=O)cc3)cc2)cc1	2	2	1	1
157	COc1cc(C=O)c(OC)cc1C=O	11	2	1	1
158	COc1ccc(N)nc1	1	2	1	1
159	O=Cc1ccc(-c2ccc(C=O)s2)s1	3	2	1	1
160	Nc1ccc(-c2cc(-c3ccc(N)cc3)cc(-c3cc(-c4ccc(N)cc4)cc(-c4ccc(N)cc4)c3)c2)cc1	1	1	0	1
161	N#CC1=NC2=C3N=C(C#N)C(C#N)=C4N=C5N=C6C(C#N)=C(C#N)N=C7C8=C9C(=NC%10=NC(=C1C#N)C2C1C%10C9C(C67)C5C1C43)C(C#N)C(C#N)=N8	1	0	0	0
162	Nc1ccc2c(c1)c(-c1ccccc1)[n+](Br)c1cc(N)ccc21	1	0	0	0
163	Nc1ccc(/C2=C3\C=CC(=N3)/C(c3ccc(N)cc3)=c3/cc/c4n3[Cu]n3c2ccc3/C(c2ccc(N)cc2)=C2/C=CC(=N2)/C=4c2ccc(N)cc2)cc1	1	0	0	0
164	CCCCC(CC)CN1C(=O)C2=C(c3ccc(B(O)O)s3)N(CC(CC)CCCC)C(=O)C2=C1c1ccc(B(O)O)s1	1	1	0	1

Table S3 (continued): List of the 359 unique monomers and index

Index	SMILES	Frequency	Flag	AZF	ASKCOS
165	O=Cc1ccc(-c2cc(-c3ccc(C=O)cc3)cc(-c3nc4cc(-c5ccc(C=O)cc5)c(-c5ccc(C=O)cc5)cc4[nH]3)c2)cc1	1	1	0	1
166	CN(C)Cc1ccc(N)cc1	1	2	1	1
167	O=Cc1ccc(C=O)s1	1	2	1	1
168	O=Cc1cc(OCCOc2cccc3cccnc23)c(C=O)cc1OCCOc1cccc2cccnc12	1	2	1	1
169	O=Cc1ccc(N(c2ccc(C=O)cc2)c2ccc(C=O)cc2)cc1	6	2	1	1
170	CCCCCCCCN1C(=O)c2cc(-c3ccc(C=O)cc3)c3c4c(cc(-c5ccc(C=O)cc5)c(c24)C1=O)C(=O)N(CCCCCCCC)C3=O	1	0	0	0
171	Nc1ccc(-c2ccc(-c3ccc(N)cc3)cc2)cc1	2	2	1	1
172	Nc1ccc(-c2cc(-c3ccc(N)cc3)c(-c3ccc(C=O)cc3)cc2-c2ccc(C=O)cc2)cc1	1	1	0	1
173	C=CCOc1cc(C(=O)NN)c(OCC=C)cc1C(=O)NN	2	2	1	1
174	CC1=C(C=O)C(C)=[N+]2C1=C(c1ccccc1)c1c(C)c(C=O)c(C)n1[B-]2(F)F	1	0	0	0
175	CC(C)(C)OC(=O)N1CCC[C@H]1COc1c(-c2ccc(N)cc2)cc(-c2ccc(N)cc2)cc1-c1ccc(N)cc1	1	2	1	1
176	C[C@@H]1CNCCN1	1	2	1	1
177	O=Cc1ccc(C=O)c2c1OCCO2	1	2	1	1
178	C=CCn1cc[n+](Cc2cc(C(=O)NN)ccc2C(=O)NN)c1.[Br-]	1	0	0	0
179	O=Cc1ccc(-c2ccc(-c3ccc(C=O)cc3)c3[nH]c(N[C@H]4c5ccccc5C[C@H]4O)nc23)cc1	1	1	0	1
180	Nc1cc(Cl)ccn1	1	2	1	1
181	Nc1ccc(NC(=O)c2cc(C(=O)Nc3ccc(N)cc3)cc(C(=O)Nc3ccc(N)cc3)c2)cc1	2	2	1	1
182	C=CCOc1cc(C=O)c(OCC=C)cc1C=O	1	2	1	1
183	O=Cc1cc2cc3sc(C=O)cc3cc2s1	2	2	1	1
184	N#CCc1ccc(-c2ccc(CC#N)cc2)cc1	2	2	1	1
185	CC(C)(C)[C@@H](CO)Nc1nc2c(-c3ccc(C=O)cc3)ccc(-c3ccc(C=O)cc3)c2[nH]1	1	2	1	1
186	Cc1c(C)c(-c2ccc(N)cc2)c2[nH]cnc2c1-c1ccc(N)cc1	1	1	0	1
187	Nc1ccc(C#CC#Cc2ccc(N)cc2)cc1	1	2	1	1
188	NNC(=O)c1cc(OCCC(F)(F)C(F)(F)C(F)(F)C(F)(F)F)c(C(=O)NN)cc1OCCC(F)(F)C(F)(F)C(F)(F)C(F)(F)F	1	1	0	1
189	O=Cc1ccc(/C=C/c2ccc(C=O)s2)s1	1	1	0	1
190	Nc1ccc(-c2ccc(N)cc2C(=O)O)c(C(=O)O)c1	2	2	1	1
191	O=Cc1ccc(N(c2ccc(C=O)cc2)c2ccc(-c3ccc(-c4ccc(N(c5ccc(C=O)cc5)c5ccc(C=O)cc5)cc4)c4nsnc34)cc2)cc1	1	1	0	1

Table S3 (continued): List of the 359 unique monomers and index

Index	SMILES	Frequency	Flag	AZF	ASKCOS
192	O=Cc1ccc(-c2ccc3c4c(ccc3c2)OCCOCCOc2ccc3cc(-c5ccc(C=O)cc5)ccc3c2-c2c(ccc3cc(-c5ccc(C=O)cc5)ccc23)OCCOCCOc2ccc3cc(-c5ccc(C=O)cc5)ccc3c2-4)cc1	1	0	0	0
193	Cc1cc(C=O)c(O)c(C=O)c1	1	2	1	1
194	Cc1c(C)c(-c2ccc(N)cc2)c2[nH]c(C(F)(F)F)nc2c1-c1ccc(N)cc1	1	1	1	0
195	OB(O)c1ccc(-c2ccc(-c3ccc(B(O)O)cc3)c3c2-c2cccc4cccc-3c24)cc1	1	0	0	0
196	Cc1nc(C)c(C#N)c(C)c1C#N	1	0	0	0
197	CC1(C)c2cc(C=O)ccc2-c2c1c1c(c3c2C(C)(C)c2cc(C=O)ccc2-3)C(C)(C)c2cc(C=O)ccc2-1	1	0	0	0
198	O=Cc1cc2sc(C=O)cc2s1	4	1	1	0
199	C=Cn1ccnc1	1	1	1	0
200	Nc1ccc(-c2ccc(N)c(Cl)c2)cc1Cl	1	1	1	0
201	O=Cc1ccc(P(c2ccc(C=O)cc2)c2ccc(C=O)cc2)cc1	3	1	0	1
202	Nc1ccc2c(c1)c1cc(N)ccc1c1c3ccc(N)cc3c3cc(N)ccc3c21	1	0	0	0
203	Nc1ccc(-c2cc(N)cc(-c3ccc(N)cc3)c2)cc1	1	2	1	1
204	CC(C)(C)[C@H]1Cn2c(nc3c(-c4ccc(C=O)cc4)ccc(-c4ccc(C=O)cc4)c32)N1	1	0	0	0
205	O=C1OC(=O)c2ccc3c4c(ccc1c24)C(=O)OC3=O	2	1	0	1
206	Nc1ccc(C2=CC(c3ccc(C=O)cc3)=C3C=CC4=C(c5ccc(N)cc5)C=C(c5ccc(C=O)cc5)C5=CC=C2C3C54)cc1	1	0	0	0
207	Nc1cc(Cl)c(N)cc1Cl	1	2	1	1
208	Cc1c(C)c(N)c2[nH]cnc2c1N	1	1	0	1
209	O=Cc1ccc(-c2cc3c(cc2-c2ccc(C=O)cc2)OCCOCCOc2cc(-c4ccc(C=O)cc4)c(-c4ccc(C=O)cc4)cc2OCCOCCO3)cc1	1	1	0	1
210	O=Cc1ccc(-c2cc(-c3ccc(C=O)cc3)c(O)c(-c3cc(-c4ccc(C=O)cc4)cc(-c4ccc(C=O)cc4)c3O)c2)cc1	1	1	0	1
211	O=C1c2cc(F)c(F)cc2C(=O)c2cc(F)c(F)cc21	1	2	1	1
212	Nc1cc(N)c(N)cc1N	2	2	1	1
213	Cc1cc(-c2ccc(NC(N)=S)c(C)c2)ccc1NC(N)=S	1	2	1	1
214	O=Cc1ccc(C(=C(c2ccc(C=O)cc2)c2ccc(C=O)cc2)c2ccc(C=O)cc2)cc1	3	1	0	1
215	N#CC1=CC2=C3C=C(C#N)C(C#N)=C4C=C5C=C6C(C#N)=C(C#N)C=C7C8=CC(C#N)=C(C#N)C9=CC%10=CC(=C1C#N)C2C1C%10C(C89)C(C76)C5C1C43	1	0	0	0
216	Nc1ccc(-c2ccc3c(c2)c2cc(-c4ccc(N)cc4)ccc2n3-n2c3ccc(-c4ccc(N)cc4)cc3c3cc(-c4ccc(N)cc4)ccc32)cc1	2	1	0	1
217	COc1c(-c2ccc(N)cc2)cc(-c2ccc(N)cc2)cc1-c1cc(-c2ccc(N)cc2)cc(-c2ccc(N)cc2)c1OC	1	1	0	1

Table S3 (continued): List of the 359 unique monomers and index

Index	SMILES	Frequency	Flag	AZF	ASKCOS
218	O=Cc1ccc2c(c1)c1cc(C=O)ccc1n2-n1c2ccc(C=O)cc2c2cc(C=O)ccc21	1	1	0	1
219	O=Cc1ccc(C#Cc2ccc(C#Cc3ccc(C=O)cc3)cc2)cc1	1	2	1	1
220	COC(=O)[C@@H]1C[C@@H](OCc2c(-c3ccc(N)cc3)cc(-c3ccc(N)cc3)cc2-c2ccc(N)cc2)CN1C(=O)OC(C)(C)C	1	1	0	1
221	Cc1cc(-c2ccc(N)cc2)c(C)cc1-c1ccc(N)cc1	1	2	1	1
222	O=Cc1ccc(-c2ccc(N(c3ccc(-c4ccc(C=O)cc4)cc3)c3ccc(-c4ccc(C=O)cc4)cc3)cc2)cc1	1	2	1	1
223	NNC(=O)c1ccc(N)cc1	1	2	1	1
224	Nc1ccc(C(=O)NNC(=O)c2ccc(N)cc2)cc1	1	2	1	1
225	Cc1cc(-c2ccc(-c3ccc(C(=O)Oc4ccccn4)cc3)c(C)c2)ccc1-c1ccc(C(=O)Oc2ccccn2)cc1	1	2	1	1
226	CC(C)COc1cc(C(=O)NN)c(OCC(C)C)cc1C(=O)NN	1	1	0	1
227	NC(=O)Nc1ccc(NC(N)=O)cc1	1	2	1	1
228	COc1c(CO)c(OC)c(CO)c(OC)c1CO	1	1	1	0
229	O=C(Oc1ccccn1)c1ccc(-c2ccc(C(=O)Oc3ccccn3)cc2)cc1	1	1	1	0
230	N#Cc1c(C#N)c(C#N)c2cc3c(C#N)c(C#N)c(C#N)c(C#N)c3cc2c1C#N	1	0	0	0
231	CCOc1cc(C=O)c2cc(Cl)ccc2c1-c1c(OCC)cc(C=O)c2cc(Cl)ccc12	1	0	0	0
232	O=Cc1ccc2cc(C=O)ccc2c1	2	2	1	1
233	Nc1ccc(-c2c(Cl)c(Cl)c(-c3ccc(N)cc3)c3nsnc23)cc1	1	1	0	1
234	Nc1ccc(NC(=O)c2ccc(N)cc2)cc1	1	2	1	1
235	CCOc1c2cc(C=O)sc2c(OCC)c2cc(C=O)sc12	1	0	0	0
236	O=Cc1cc(C=O)c(O)c(C=O)c1O	2	2	1	1
237	O=Cc1ccc(-c2ccc(-c3ccc(C=O)cc3)c3[nH]c(N[C@@H](CO)c4ccccc4)nc23)cc1	1	2	1	1
238	Cc1nc2c(-c3ccc(N)cc3)c(C)c(C)c(-c3ccc(N)cc3)c2[nH]1	1	2	1	1
239	CC(O)c1nc2c(-c3ccc(N)cc3)ccc(-c3ccc(N)cc3)c2[nH]1	1	2	1	1
240	CCCCN1C(=O)C2=C(c3ccc(C=O)cc3)N(CCCC)C(=O)C2=C1c1ccc(C=O)cc1	1	0	0	0
241	O=Cc1ccc(-c2cc(-c3ccc(-c4cc(-c5ccc(C=O)cn5)nc(-c5ccc(C=O)cn5)c4)cc3)cc(-c3ccc(C=O)cn3)n2)nc1	1	1	0	1
242	O=Cc1ccc(-c2ccc(-c3ccc(C=O)cc3)c3nsnc23)cc1	1	1	0	1
243	[C-]#[N+]c1ccc(-c2cc(-c3ccc([N+]#[C-])cc3)cc(-c3ccc([N+]#[C-])cc3)c2)cc1	1	1	0	1
244	O=Cc1ccc(-c2cc(-c3ccc(C=O)cc3)c(OCC3CCCN3C(=O)C(F)(F)F)c(-c3ccc(C=O)cc3)c2)cc1	1	1	0	1
245	Nc1ccc(-c2cc(-c3ccc(N)cc3)c(COC3CCn4ccnc43)c(-c3ccc(N)cc3)c2)cc1	1	1	0	1
246	Oc1ccc(C(=C(c2ccc(O)cc2)c2ccc(O)cc2)c2ccc(O)cc2)cc1	1	1	0	1

Table S3 (continued): List of the 359 unique monomers and index

Index	SMILES	Frequency	Flag	AZF	ASKCOS
247	CN(C)/C=C\C(=O)c1cc(C(=O)/C=C\N(C)C)cc(C(=O)/C=C\N(C)C)c1	1	0	0	0
248	O=Cc1ccc(OP2(Oc3ccc(C=O)cc3)=NP(Oc3ccc(C=O)cc3)(Oc3ccc(C=O)cc3)=NP(Oc3 ccc(C=O)cc3)(Oc3ccc(C=O)cc3)=N2)cc1	1	0	0	0
249	O=Cc1ccc(C=O)c(F)c1	1	2	1	1
250	NNC(=O)c1ccc(C(=O)NN)c(/N=N/c2ccccc2)c1	1	1	0	1
251	O=Cc1ccc(-c2ccc(C(=C(c3ccc(-c4ccc(C=O)cc4)cc3)c3ccc(-c4ccc(C=O)cc4)cc3)c3ccc(-c4ccc(C=O)cc4)cc3)cc2)cc1	3	1	0	1
252	O=Cc1ccc(-c2cc(C(=O)O)c(-c3ccc(C=O)cc3)cc2C(=O)O)cc1	1	1	0	1
253	Nc1nc2cc3sc(N)nc3cc2s1	1	2	1	1
254	Nc1ccc(-c2ccc(-c3nc(-c4ccc(-c5ccc(N)cc5)cc4)nc(-c4ccc(-c5ccc(N)cc5)cc4)n3)cc2)cc1	1	1	0	1
255	CC(C)(C)OC(=O)N1CCC[C@@H]1CNc1nc2c(-c3ccc(C=O)cc3)ccc(-c3ccc(C=O)cc3)c2[nH]1	1	2	1	1
256	Nc1ccc(-c2cc(/C=C/c3cc(-c4ccc(N)cc4)cc(-c4ccc(N)cc4)c3)cc(-c3ccc(N)cc3)c2)cc1	1	1	0	1
257	Nc1ccc2c(c1)nc(-c1ccccc1)c1cc(N)ccc12	1	1	0	1
258	Cc1cc(-c2ccc(C(=O)NN)cc2)ccc1-c1ccc(C(=O)NN)cc1	1	2	1	1
259	OB(O)c1ccc(-c2cc(-c3ccccc3)c(-c3ccc(B(O)O)cc3)c3c2-c2cccc4cccc-3c24)cc1	1	0	0	0
260	O=Cc1ccc(-c2ccc(-c3ccc(C=O)cc3)c3[nH]c(N[C@H](CO)Cc4ccccc4)nc23)cc1	1	1	0	1
261	OB(O)c1ccc(-c2c(-c3ccccc3)c(-c3ccccc3)c(-c3ccc(B(O)O)cc3)c3c2-c2cccc4cccc-3c24)cc1	1	0	0	0
262	Nc1ccc(-c2c3nc(c(-c4ccc(-c5ccc(C=O)cc5)cc4)c4ccc([nH]4)c(-c4ccc(N)cc4)c4nc(c(-c5ccc(-c6ccc(C=O)cc6)cc5)c5ccc2[nH]5)C=C4)C=C3)cc1	1	1	0	1
263	O=Cc1ccc(/C(=C(\c2ccccc2)c2ccc(C=O)cc2)c2ccccc2)cc1	1	1	0	1
264	Cc1c(-c2ccc([N+](=O)[O-])cc2)c(C)c(-c2ccc([N+](=O)[O-])cc2)c(C)c1-c1ccc([N+](=O)[O-])cc1	1	1	0	1
265	C=Cc1ccc(Cn2c[n+](C)c3ccccc32)cc1.[Br-]	1	0	0	0
266	O=Cc1ccc(-c2c3ccoc3c(-c3ccc(C=O)cc3)c3ccoc23)cc1	1	1	0	1
267	Nc1ccc(-c2ccc3c(c2)-c2cc(-c4ccc(N)cc4)ccc2C3=C2c3cc(-c4ccc(N)cc4)cc3-c3cc(-c4ccc(N)cc4)ccc32)cc1	1	1	0	1
268	Nc1ccc(N(c2ccc(N)cc2)c2ccc(N(c3ccc(N)cc3)c3ccc(N)cc3)cc2)cc1	2	2	1	1
269	O=Cc1ccc(C#Cc2cc(C#Cc3ccc(C=O)c(O)c3)cc(C#Cc3ccc(C=O)c(O)c3)c2)cc1O	1	1	0	1

Table S3 (continued): List of the 359 unique monomers and index

Index	SMILES	Frequency	Flag	AZF	ASKCOS
270	O=Cc1ccc(-c2ccc(-c3ccc(C(=C(c4ccc(-c5ccc(-c6ccc(C=O)cc6)cc5)cc4)c4ccc(-c5ccc(-c6ccc(C=O)cc6)cc5)cc4)c4ccc(-c5ccc(-c6ccc(C=O)cc6)cc5)cc4)cc3)cc2)cc1	1	1	0	1
271	CCc1cc(C=O)c(CC)cc1C=O	1	2	1	1
272	Nc1ccc(/C2=C3\C=CC(=N3)/C(c3ccc(N)cc3)=c3/cc/c4n3[Ni]n3c2ccc3/C(c2ccc(N)cc2)=C2/C=CC(=N2)/C=4c2ccc(N)cc2)cc1	1	0	0	0
273	COC(=O)C1CC(=O)C(C(=O)OC)CC1=O	1	2	1	1
274	CCCCCCOc1cc(C(=O)NN)c(OCCCCCC)cc1C(=O)NN	1	2	1	1
275	CCCOc1c2cc(C=O)sc2c(OCCC)c2cc(C=O)sc12	1	0	0	0
276	O=Cc1ccc2c(c1)c1c3ccccc3c3c4cc(C=O)ccc4c4c5ccc(C=O)cc5c5c6ccccc6c6c7cc(C=O)ccc7c2c2c1c3c4c5c62	1	0	0	0
277	Clc1nc(Cl)nc(Cl)n1	1	1	1	0
278	Cc1nc(C)nc(C)n1	2	0	0	0
279	O=Cc1ccc(-c2cc(-c3ccc(C=O)c(F)c3)cc(-c3ccc(C=O)c(F)c3)c2)cc1F	2	1	0	1
280	Nc1ccc(-c2cc(-c3ccc(C=O)cc3)c(-c3ccc(N)cc3)cc2-c2ccc(C=O)cc2)cc1	1	1	0	1
281	Nc1cc2c3c(c1)Oc1cc(N)cc4c1N3c1c(cc(N)cc1O4)O2	1	0	0	0
282	Nc1ccc(-c2ccc(N)cn2)nc1	1	1	0	1
283	c1ccc(C(=Nc2ccc3c(c2)c2cc(N=C(c4ccccc4)c4ccccc4)ccc2c2c4ccc(N=C(c5ccccc5)c5ccccc5)cc4c4cc(N=C(c5ccccc5)c5ccccc5)ccc4c32)c2ccccc2)cc1	1	1	0	1
284	O=Cc1ccc(-c2cc(-c3ccc(C=O)c(C(F)(F)F)c3)cc(-c3ccc(C=O)c(C(F)(F)F)c3)c2)cc1C(F)(F)F	1	1	0	1
285	Cc1cc(NC(N)=S)c(C)cc1NC(N)=S	1	2	1	1
286	CN1c2ccc(N)cc2Oc2ccc(N)cc21	1	1	0	1
287	Nc1ccc(-c2cc(-c3ccc(C=O)cc3)c(-c3ccc(C=O)cc3)cc2-c2ccc(N)cc2)cc1	1	2	1	1
288	Oc1ccc(-c2ccc(-c3cc(-c4ccc(-c5ccc(O)cc5)cc4)cc(-c4ccc(-c5ccc(O)cc5)cc4)c3)cc2)cc1	1	1	0	1
289	NNC(=O)c1cc(OCCC(F)(F)C(F)(F)C(F)(F)C(F)(F)C(F)(F)C(F)(F)C(F)(F)C(F)(F)F)c(C(=O)NN)cc1OCCC(F)(F)C(F)(F)C(F)(F)C(F)(F)C(F)(F)C(F)(F)C(F)(F)C(F)(F)F	1	1	0	1
290	O=Cc1ccc(-c2ccc(-c3cc(-c4ccc(-c5ccc(C=O)cc5)cc4)c(-c4ccc(-c5ccc(C=O)cc5)cc4)cc3-c3ccc(-c4ccc(C=O)cc4)cc3)cc2)cc1	1	1	0	1
291	O=Cc1cc(Cl)c(C=O)cc1Cl	1	2	1	1
292	O=Cc1ccc(-c2c3c(c(-c4ccc(C=O)cc4)c4nsnc24)N=S=N3)cc1	1	1	0	1
293	Nc1ccc(Nc2c(Nc3ccc(N)cc3)c(=O)c2=O)cc1	1	2	1	1
294	CCOP(=O)(CCOc1cc(C(=O)NN)c(OCCP(=O)(OCC)OCC)cc1C(=O)NN)OCC	1	2	1	1

Table S3 (continued): List of the 359 unique monomers and index

Index	SMILES	Frequency	Flag	AZF	ASKCOS
295	Nc1ccc(-c2cc(-c3ccc(N)cc3)nc(-c3ccc(N)cc3)c2)cc1	2	2	1	1
296	C#Cc1ccccc1	1	2	1	1
297	Nc1ccc(-c2ccc(-c3ccc(N)cc3)c3nsnc23)cc1	1	2	1	1
298	Nc1ccc(C#Cc2cc(-c3ccc(C=O)cc3)c(C#Cc3ccc(N)cc3)cc2-c2ccc(C=O)cc2)cc1	1	1	0	1
299	NNc1ccc(-c2nc(-c3ccc(NN)cc3)nc(-c3ccc(NN)cc3)n2)cc1	1	2	1	1
300	Nc1ccc2c(c1)Cc1cc(N)ccc1-2	1	2	1	1
301	O=Cc1ccc(-c2ccc3c(c2)c2cc(-c4ccc(C=O)cc4)ccc2n3-n2c3ccc(-c4ccc(C=O)cc4)cc3c3cc(-c4ccc(C=O)cc4)ccc32)cc1	1	1	0	1
302	CCCOc1cc(C(=O)NN)c(OCCC)cc1C(=O)NN	1	2	1	1
303	O=Cc1cc(C=O)cc(B(O)O)c1	1	2	1	1
304	O=[N+]([O-])c1ccc(-[n+]2ccc(-c3cc[n+](-c4ccc([N+](=O)[O-])cc4[N+](=O)[O-])cc3)cc2)c([N+](=O)[O-])c1	1	0	0	0
305	CN1C(=O)C(Cc2c(-c3ccc(N)cc3)cc(-c3ccc(N)cc3)cc2-c2ccc(N)cc2)N(C(=O)OC(C)(C)C)[C@H]1C(C)(C)C	1	0	0	0
306	Nc1ccc(-c2ccc(N)c(N)c2)cc1N	1	2	1	1
307	Cc1cc(N)cc2c1C1C3CC(C21)C1c2c(C)cc(N)cc2C31	1	0	0	0
308	N#Cc1cc2c3cc(C#N)c(C#N)cc3c3cc4c5cc(C#N)c(C#N)cc5c5cc(C#N)c(C#N)cc5c4cc3c2cc1C#N	1	1	0	1
309	Nc1ccc(-c2cc(-c3ccc(N)cc3)c(O)c(-c3cc(-c4ccc(N)cc4)cc(-c4ccc(N)cc4)c3O)c2)cc1	1	1	0	1
310	O=C(Oc1ccccn1)c1ccc(C(=O)Oc2ccccn2)cc1	1	1	1	0
311	NNC(=O)c1cc(OCCC(F)(F)C(F)(F)C(F)(F)C(F)(F)C(F)(F)C(F)(F)F)c(C(=O)NN)cc1OCCC(F)(F)C(F)(F)C(F)(F)C(F)(F)C(F)(F)C(F)(F)F	1	1	0	1
312	Cc1c(C#N)c(C)c(C#N)c(C)c1C#N	1	0	0	0
313	COc1c(-c2ccc(C=O)cc2)cc(-c2ccc(C=O)cc2)cc1-c1cc(-c2ccc(C=O)cc2)cc(-c2ccc(C=O)cc2)c1OC	1	1	0	1
314	O=Cc1ccc(-c2ccc3c(c2)-c2cc(-c4ccc(C=O)cc4)ccc2C3C2c3ccc(-c4ccc(C=O)cc4)cc3-c3cc(-c4ccc(C=O)cc4)ccc32)cc1	1	0	0	0
315	Nc1ccc(-c2ccc(-c3ccc(N)cc3)c3c2OCCOCCOCCOCCO3)cc1	1	1	0	1
316	N#CCc1ccc(-c2ccc(CC#N)cn2)nc1	1	1	0	1
317	Nc1ccc(Oc2cc(Oc3ccc(N)cc3)cc(Oc3ccc(N)cc3)c2)cc1	1	2	1	1
318	O=Cc1cc(C=O)cc(-c2cccc(-c3cc(C=O)cc(C=O)c3)c2)c1	1	2	1	1
319	Cc1ccc(-c2cc(N)c(-c3ccc(C)s3)cc2N)s1	1	1	0	1
320	Cc1cc(N)ncc1Br	1	2	1	1
321	CCSCc1cc(-c2ccc(N)cc2)c(CSCC)cc1-c1ccc(N)cc1	1	1	0	1

Table S3 (continued): List of the 359 unique monomers and index

Index	SMILES	Frequency	Flag	AZF	ASKCOS
322	O=Cc1ccc(-c2cc(-c3ccc(C=O)c(O)c3)cc(-c3ccc(C=O)c(O)c3)c2)cc1O	1	2	1	1
323	O=Cc1ccc(N(c2ccc(C=O)cc2)c2ccc(-c3cc(C=O)c(-c4ccc(N(c5ccc(C=O)cc5)c5ccc(C=O)cc5)cc4)cc3C=O)cc2)cc1	1	0	0	0
324	NC(=S)Nc1ccc(NC(N)=S)cc1	1	2	1	1
325	O=Cc1ccc(/C2=C3\C=CC(=N3)/C(c3ccc(C=O)cc3)=c3/cc/c4n3[Zn]n3c2ccc3/C(c2ccc(C=O)cc2)=C2/C=CC(=N2)/C=4c2ccc(C=O)cc2)cc1	1	0	0	0
326	CC(C)[C@@H](CN(C)C)Nc1nc2c(-c3ccc(C=O)cc3)ccc(-c3ccc(C=O)cc3)c2[nH]1	1	0	0	0
327	c1ccc(C(=Nc2ccc3nc4cc(N=C(c5ccccc5)c5ccccc5)ccc4nc3c2)c2ccccc2)cc1	1	1	0	1
328	Nc1ccc(-c2ccc(-c3ccc(N)cc3)c3c2OCCOCCOCCOCCOCCO3)cc1	1	1	0	1
329	Nc1ccc(-c2ccc(-c3ccc(N)cc3)c3c2OCCOCCOCCO3)cc1	1	2	1	1
330	N/N=c1\[nH][nH]/c(=N\N)s1	1	0	0	0
331	O=Cc1ccc(-c2cc(-c3ccc(C=O)cn3)cc(-c3ccc(C=O)cn3)c2)nc1	1	2	1	1
332	O=CC1=C(C=O)SC(=C2SC(C=O)=C(C=O)S2)S1	1	0	0	0
333	OB(O)c1ccc(-c2ccccc2-c2ccc(B(O)O)cc2)cc1	1	2	1	1
334	O=Cc1ccc(-c2cc(-c3ccc(C=O)s3)cc(-c3ccc(C=O)s3)c2)s1	1	1	0	1
335	CC(C)c1cc(-c2c(F)c(-c3cc(C(C)C)c(N)c(C(C)C)c3)c(F)c(-c3cc(C(C)C)c(N)c(C(C)C)c3)c2F)cc(C(C)C)c1N	1	0	0	0
336	Nc1ccc(C2=C(c3ccc(N)cc3)SC(=C3SC(c4ccc(N)cc4)=C(c4ccc(N)cc4)S3)S2)cc1	1	0	0	0
337	Nc1ccc(-c2cc3cc(-c4ccc(N)cc4)cc4c5cc(-c6ccc(N)cc6)cc6cc(-c7ccc(N)cc7)cc(c(c2)c34)c65)cc1	1	0	0	0
338	COc1cc(-c2cc(-c3ccc(C(=O)NN)c(OC)c3)cc(-c3ccc(C(=O)NN)c(OC)c3)c2)ccc1C(=O)NN	1	0	0	0
339	Nc1ccccn1	1	1	1	0
340	COc1c2cc(C=O)sc2c(OC)c2cc(C=O)sc12	1	0	0	0
341	O=Cc1cc(C=O)cc(N2c3ccccc3N(c3cc(C=O)cc(C=O)c3)c3ccccc32)c1	1	0	0	0
342	Nc1c(F)cc(-c2cc(-c3cc(F)c(N)c(F)c3)cc(-c3cc(F)c(N)c(F)c3)c2)cc1F	1	1	1	0
343	Nc1ccc(C#CC2=CC(c3ccc(C=O)cc3)C3=CCc4c(C#Cc5ccc(N)cc5)cc(-c5ccc(C=O)cc5)c5ccc2c3c45)cc1	1	0	0	0
344	Cc1cc(N)c(C)c2c1C1C3CC(C4c5c(C)cc(N)c(C)c5C34)C21	1	0	0	0
345	Nc1ccc(-c2c(F)c(F)c(-c3ccc(N)cc3)c3nsnc23)cc1	1	0	0	0
346	CC(C)(C)c1cc(C#Cc2cc(C#Cc3cc(C=O)c(O)c(C(C)(C)C)c3)cc(C#Cc3cc(C=O)c(O)c(C(C)(C)C)c3)c2)cc(C=O)c1O	1	0	0	0
347	C=Cc1cc(C=O)c(C=C)cc1C=O	1	0	0	0
348	COC(=O)[C@@H]1C[C@H](Oc2c(-c3ccc(N)cc3)cc(-c3ccc(N)cc3)cc2-c2ccc(N)cc2)CN1C(=O)OC(C)(C)C	1	0	0	0

Table S3 (continued): List of the 359 unique monomers and index

Index	SMILES	Frequency	Flag	AZF	ASKCOS
349	CC[C@H](C)COc1cc(C(=O)NN)c(OC[C@@H](C)CC)cc1C(=O)NN	1	0	0	0
350	CN(C)[C@H]1CCCC[C@@H]1Nc1nc2c(-c3ccc(C=O)cc3)ccc(-c3ccc(C=O)cc3)c2[nH]1	1	0	0	0
351	O=Cc1ccc(C#Cc2cc(C#Cc3ccc(C=O)cc3)cc(C#Cc3ccc(C=O)cc3)c2)cc1	1	1	1	0
352	Cc1c(-c2ccc(C=O)cc2)c(C)c(-c2ccc(C=O)cc2)c(C)c1-c1ccc(C=O)cc1	1	0	0	0
353	Nc1ccc(C#Cc2ccc(N)cc2)cc1	1	1	1	0
354	Cc1c(O)c(O)c(C)c(O)c1O	1	0	0	0
355	CCc1c(O)c(O)c(CC)c(O)c1O	1	1	1	0
356	CCCc1c(O)c(O)c(CCC)c(O)c1O	1	1	1	0
357	Nc1cc(S(=O)(=O)O)c(N)cc1S(=O)(=O)O	1	1	1	0
358	O=Cc1ccc(-c2cc(-c3ccc(C=O)nc3)cc(-c3ccc(C=O)nc3)c2)cn1	1	1	1	0

Table S4: List of bromide linkers (Linker_C)

Linker Name	SMILES	PubChem IUPAC Name	Solution
linker1_C	C1=CC(=C(C=C1Br)N)Br	2,5-dibromoaniline	
linker2_C	C1=CC(=C(C=C1Br)O)Br	2,5-dibromophenol	
linker3_C	CC1=C(C=CC(=C1)Br)Br	1,4-dibromo-2-methylbenzene	
linker4_C	C1=CC(=C(C=C1Br)[N+](=O)[O-])Br	1,4-dibromo-2-nitrobenzene	
linker5_C	CC1=CC(=CC(=C1Br)C)Br	2,5-dibromo-1,3-dimethylbenzene	
linker6_C	BrC1=CC([N+]([O-])=O)=C(Br)C([N+]([O-])=O)=C1	2,5-dibromo-1,3-dinitrobenzene	
linker7_C	CC1=CC(=C(C=C1Br)C)Br	1,4-dibromo-2,5-dimethylbenzene	
linker8_C	C1=C(C(=CC(=C1Br)[N+](=O)[O-])Br)[N+](=O)[O-]	1,4-dibromo-2,5-dinitrobenzene	
linker9_C	CC1=C(C=CC(=C1C)Br)Br	1,4-dibromo-2,3-dimethylbenzene	
linker10_C	C1=CC(=C(C(=C1Br)[N+](=O)[O-])[N+](=O)[O-])Br	1,4-dibromo-2,3-dinitrobenzene	
linker11_C	C1(=C(C(=C(C(=C1Br)N)N)Br)N)N	3,6-dibromobenzene-1,2,4,5-tetramine	
linker12_C	BrC1=C(O)C(O)=C(Br)C(O)=C1O	3,6-dibromobenzene-1,2,4,5-tetrol	
linker13_C	CC1=C(C(=C(C(=C1Br)C)C)Br)C	1,4-dibromo-2,3,5,6-tetramethylbenzene	
linker14_C	BrC1=CC=C(C2=CC=C(Br)C=C2CO)C(CO)=C1	[5-bromo-2-[4-bromo-2-(hydroxymethyl)phenyl]phenyl]methanol	
linker15_C	BrC1=CC=C(C2=CC=C(Br)C=C2N)C=C1	5-bromo-2-(4-bromophenyl)aniline	
linker16_C	NC1=CC(Br)=CC=C1C2=CC=C(Br)C=C2N	2-(2-amino-4-bromophenyl)-5-bromoaniline	
linker17_C	BrC1=CC([N+]([O-])=O)=C(C2=C(N)C=C(Br)C([N+]([O-])=O)=C2)C=C1N	5-(2-amino-4-bromo-5-nitrophenyl)-2-bromo-4-nitroaniline	
linker18_C	BrC1=C(N)C=C(C2=C(N)C=C(Br)C([N+]([O-])=O)=C2)C=C1[N+]([O-])=O		ASK+AZF
linker19_C	BrC1=CC=C(C2=CC=C(Br)C=C2)C=C1	1-bromo-4-(4-bromophenyl)benzene	
linker20_C	BrC1=CC=C(C2=CC=C(Br)C=C2C)C(C)=C1	4-bromo-1-(4-bromo-2-methylphenyl)-2-methylbenzene	
linker21_C	BrC1=CC(C)=C(C2=C(C)C=C(Br)C=C2C)C(C)=C1	5-bromo-2-(4-bromo-2,6-dimethylphenyl)-1,3-dimethylbenzene	

Table S4(continued): List of bromide linkers (Linker_C)

Linker Name	SMILES	PubChem IUPAC Name	Solution
linker22_C	BrC1=CC=C(C2=C([N+]([O-])=O)C=C(Br)C=C2)C=C1	4-bromo-1-(4-bromophenyl)-2-nitrobenzene	
linker23_C	BrC1=CC([N+]([O-])=O)=C(C2=C([N+]([O-])=O)C=C(Br)C=C2)C=C1	4-bromo-1-(4-bromo-2-nitrophenyl)-2-nitrobenzene	
linker24_C	BrC1=CC([N+]([O-])=O)=C(C2=CC=C(Br)C=C2)C([N+]([O-])=O)=C1	5-bromo-2-(4-bromophenyl)-1,3-dinitrobenzene	
linker25_C	BrC1=CC([N+]([O-])=O)=C(C2=CC=C(Br)C([N+]([O-])=O)=C2)C=C1	1-bromo-4-(4-bromo-2-nitrophenyl)-2-nitrobenzene	
linker26_C	BrC1=CC([N+]([O-])=O)=C(C2=C([N+]([O-])=O)C=C(Br)C=C2[N+]([O-])=O)C([N+]([O-])=O)=C1	5-bromo-2-(4-bromo-2,6-dinitrophenyl)-1,3-dinitrobenzene	
linker27_C	BrC1=CC2=C(C3=C([N+]([O-])=O)C=C(Br)C=C3CC2)C=C1	2,7-dibromo-4-nitro-9,10-dihydrophenanthrene	
linker28_C	BrC1=CC=C(C2=CC=C(Br)C([N+]([O-])=O)=C2)C=C1	1-bromo-4-(4-bromophenyl)-2-nitrobenzene	
linker29_C	BrC1=CC(N)=C(C2=C(N)C=C(Br)C([N+]([O-])=O)=C2)C=C1[N+]([O-])=O	2-(2-amino-4-bromo-5-nitrophenyl)-5-bromo-4-nitroaniline	
linker30_C	BrC1=CC=C(C2=CC=C(Br)C=N2)N=C1	5-bromo-2-(5-bromopyridin-2-yl)pyridine	
linker31_C	BrC1=CC(C)=C(C2=C(C)C=C(Br)C=N2)N=C1	5-bromo-2-(5-bromo-3-methylpyridin-2-yl)-3-methylpyridine	
linker32_C	BrC1=CC(N)=C(C2=C(N)C=C(Br)C=N2)N=C1	2-(3-amino-5-bromopyridin-2-yl)-5-bromopyridin-3-amine	
linker33_C	CC(C(Br)=C1)=NC2=C1C=CC3=CC(Br)=C(C)N=C32	3,8-dibromo-2,9-dimethyl-1,10-phenanthroline	
linker34_C	BrC1=CC(C(O)=O)=C(C2=C(C(O)=O)C=C(Br)C=N2)N=C1	5-bromo-2-(5-bromo-3-carboxypyridin-2-yl)pyridine-3-carboxylic acid	
linker35_C	C1=CC2=CC(=CN=C2C3=NC=C(C=C31)Br)Br	3,8-dibromo-1,10-phenanthroline	
linker36_C	BrC1=CN=C2C3=NC(C)=C(Br)C=C3C=CC2=C1	3,8-dibromo-2-methyl-1,10-phenanthroline	

Table S4(continued): List of bromide linkers (Linker_C)

Linker Name	SMILES	PubChem IUPAC Name	Solution
linker37_C	BrC1=CC([N+]([O-])=O)=C(C2=C([N+]([O-])=O)C=C(Br)C=N2)N=C1	5-bromo-2-(5-bromo-3-nitropyridin-2-yl)-3-nitropyridine	
linker38_C	C1=C(C=NC2=C1C(=C(C3=C2N=CC(=C3)Br)O)O)Br	3,8-dibromo-1,10-phenanthroline-5,6-diol	
linker39_C	BrC1=CC2=C(N=C1)C3=NC=C(Br)C=C3N=N2	5,12-dibromo-3,8,9,14-tetrazatricyclo[8.4.0.02,7]tetradeca-1(10),2(7),3,5,8,11,13-heptaene	
linker40_C	C1=C(C=NC2=C1C(=O)C(=O)C3=C2N=CC(=C3)Br)Br	3,8-dibromo-1,10-phenanthroline-5,6-dione	
linker41_C	BrC1=C2C(OC=N2)=C(Br)C=C1	4,7-dibromo-1,3-benzoxazole	
linker42_C	BrC1=C2C(OC=N2)=C(Br)C3=C1OC=N3	4,8-dibromo-[1,3]oxazolo[5,4-f][1,3]benzoxazole	
linker43_C	BrC1=C2C(OC(C)=N2)=C(Br)C3=C1OC(C)=N3	4,8-dibromo-2,6-dimethyl-[1,3]oxazolo[5,4-f][1,3]benzoxazole	
linker44_C	BrC1=C2C(OC(CC)=N2)=C(Br)C3=C1OC(CC)=N3	4,8-dibromo-2,6-diethyl-[1,3]oxazolo[5,4-f][1,3]benzoxazole	
linker45_C	C1=CC(=C2C(=C1Br)N=CC=N2)Br	5,8-dibromoquinoxaline	
linker46_C	CC1=NC2=C(C=CC(=C2N=C1)Br)Br	5,8-dibromo-2-methylquinoxaline	
linker47_C	BrC1=CC=C(Br)C2=C1N=C3C(C=CC=C3)=N2	1,4-dibromophenazine	
linker48_C	CC1=NC2=C(C=CC(=C2N=C1C)Br)Br	5,8-dibromo-2,3-dimethylquinoxaline	
linker49_C	O=C1NC2=C(Br)C=CC(Br)=C2N=C1	5,8-dibromo-1H-quinoxalin-2-one	
linker50_C	BrC1=C2C(N=CC(NC)=N2)=C(Br)C=C1	5,8-dibromo-N-methylquinoxalin-2-amine	
linker51_C	C1CCC2=NC3=C(C=CC(=C3N=C2C1)Br)Br	6,9-dibromo-1,2,3,4-tetrahydrophenazine	
linker52_C	COC1=NC2=C(C=CC(=C2N=C1)Br)Br	5,8-dibromo-2-methoxyquinoxaline	
linker53_C	CCC1=NC2=C(C=CC(=C2N=C1CC)Br)Br	5,8-dibromo-2,3-diethylquinoxaline	
linker54_C	O=C1NC2=C(Br)C=CC(Br)=C2N=C1C(O)=O	5,8-dibromo-3-oxo-4H-quinoxaline-2-carboxylic acid	
linker55_C	C1=NC2=C(C3=NSN=C3C(=C2N=C1)Br)Br	4,9-dibromo-[1,2,5]thiadiazolo[3,4-g]quinoxaline	
linker56_C	CC1=NC2=C(C3=NSN=C3C(=C2N=C1C)Br)Br	4,9-dibromo-6,7-dimethyl-[1,2,5]thiadiazolo[3,4-g]quinoxaline	

Table S4(continued): List of bromide linkers (Linker_C)

Linker Name	SMILES	PubChem IUPAC Name	Solution
linker57_C	BrC1=C(NC=NC2=O)C2=C(Br)C=C1	5,8-dibromo-3H-quinazolin-4-one	
linker58_C	C1=C(N=CC(=N1)Br)Br	2,5-dibromopyrazine	
linker59_C	CC1=NC(=CN=C1Br)Br	2,5-dibromo-3-methylpyrazine	
linker60_C	CC1=C(N=C(C(=N1)Br)C)Br	2,5-dibromo-3,6-dimethylpyrazine	
linker61_C	C1=C(N=C(C(=N1)Br)N)Br	3,6-dibromopyrazin-2-amine	
linker62_C	C1=C(N=C(C(=N1)Br)C#N)Br	3,6-dibromopyrazine-2-carbonitrile	
linker63_C	C1=C(N=C(C(=N1)Br)CO)Br	(3,6-dibromopyrazin-2-yl)methanol	
linker64_C	BrC1=NC=C(Br)N=C1C=O	3,6-dibromopyrazine-2-carbaldehyde	
linker65_C	BrC1=NC(C#N)=C(Br)N=C1C#N	3,6-dibromopyrazine-2,5-dicarbonitrile	
linker66_C	COC1=NC(=CN=C1Br)Br	2,5-dibromo-3-methoxypyrazine	
linker67_C	C1=C(N=C(C(=N1)Br)C(=O)O)Br	3,6-dibromopyrazine-2-carboxylic acid	
linker68_C	C1=C(N=C(C(=N1)Br)[N+](=O)[O-])Br	2,5-dibromo-3-nitropyrazine	
linker69_C	CC(=O)C1=NC(=CN=C1Br)Br	1-(3,6-dibromopyrazin-2-yl)ethanone	
linker70_C	COC1=NC(Br)=C(OC)N=C1Br		ASK
linker71_C	C1=C(N=C(C(=N1)Br)C(=O)N)Br	3,6-dibromopyrazine-2-carboxamide	
linker72_C	C1(=C(N=C(C(=N1)Br)C(=O)O)Br)C(=O)O	3,6-dibromopyrazine-2,5-dicarboxylic acid	
linker73_C	BrC1=NC(/N=C/N(C)C)=C(Br)N=C1	N'-(3,6-dibromopyrazin-2-yl)-N,N-dimethylmethanimidamide	
linker74_C	COC(=O)C1=NC(=CN=C1Br)Br	methyl 3,6-dibromopyrazine-2-carboxylate	
linker75_C	CCOC(=O)C1=NC(=CN=C1Br)Br	ethyl 3,6-dibromopyrazine-2-carboxylate	
linker76_C	C1=C(C=NC(=N1)Br)Br	2,5-dibromopyrimidine	
linker77_C	CC1=NC(=NC=C1Br)Br	2,5-dibromo-4-methylpyrimidine	
linker78_C	COC1=NC(=NC=C1Br)Br	2,5-dibromo-4-methoxypyrimidine	
linker79_C	C1CC1C2=NC(=NC=C2Br)Br	2,5-dibromo-4-cyclopropylpyrimidine	
linker80_C	CC(C)(C)C(N=C(Br)N1)=C(Br)C1=O	2,5-dibromo-4-tert-butyl-1H-pyrimidin-6-one	
linker81_C	C1=CC(=NN=C1Br)Br	3,6-dibromopyridazine	
linker82_C	CC1=CC(=NN=C1Br)Br	3,6-dibromo-4-methylpyridazine	
linker83_C	C1=CC=C2C(=C1)C(=NN=C2Br)Br	1,4-dibromophthalazine	
linker84_C	C1=C(C(=NN=C1Br)Br)N	3,6-dibromopyridazin-4-amine	

Table S4(continued): List of bromide linkers (Linker_C)

Linker Name	SMILES	PubChem IUPAC Name	Solution
linker85_C	BrC1=NN=C(Br)C(C(C)=O)=C1	1-(3,6-dibromopyridazin-4-yl)ethanone	
linker86_C	BrC1=NN=C(Br)C2=C1C=C(C(F)F)S2		Unsolved
linker87_C	C1=CC(=CC=C1Br)Br	1,4-dibromobenzene	
linker88_C	C1=C(C(=CC(=C1Br)O)Br)O	2,5-dibromobenzene-1,4-diol	
linker89_C	C1CC2=C(C=CC(=C2)Br)C3=C1C=C(C=C3)Br	2,7-dibromo-9,10-dihydrophenanthrene	
linker90_C	C1=CC=C2C=C3C(=CC2=C1)C(=C4C=C5C=CC=CC5=CC4=C3Br)Br	6,13-dibromopentacene	
linker91_C	C1(=NC(=NC(=N1)Br)Br)Br	2,4,6-tribromo-1,3,5-triazine	
linker92_C	C1=C(C=C(C=C1Br)Br)Br	1,3,5-tribromobenzene	
linker93_C	C1=CC(=CC=C1N(C2=CC=C(C=C2)Br)C3=CC=C(C=C3)Br)Br	4-bromo-N,N-bis(4-bromophenyl)aniline	
linker94_C	C1=CC(=CC=C1C2=CC(=CC(=C2)C3=CC=C(C=C3)Br)C4=CC=C(C=C4)Br)Br	1,3,5-tris(4-bromophenyl)benzene	
linker95_C	BrC1=CC=C(/C=C/C2=CC(/C=C/C3=CC=C(Br)C=C3)=CC(/C=C/C4=CC=C(Br)C=C4)=C2)C=C1	1,3,5-tris[(E)-2-(4-bromophenyl)ethenyl]benzene	
linker96_C	C1=CC(=CC=C1C2=NC(=NC(=N2)C3=CC=C(C=C3)Br)C4=CC=C(C=C4)Br)Br	2,4,6-tris(4-bromophenyl)-1,3,5-triazine	
linker97_C	BrC(C=C1)=CC=C1C(C=C2)=CC=C2/C3=C4C=CC(/C(C5=CC=C(C6=CC=C(Br)C=C6)C=C5)=C7C=C/C(N/7)=C(C8=CC=C(C9=CC=C(Br)C=C9)C=C8)/C(C=C/%10)=NC%10=C(C%11=CC=C(C%12=CC=C(Br)C=C%12)C=C%11)/C%13=CC=C3N%13)=N\4	5,10,15,20-tetrakis[4-(4-bromophenyl)phenyl]-21,23-dihydroporphyrin	
linker98_C	BrC1=CC=C(/C2=C3C=CC(/C(C4=CC=C(Br)C=C4)=C5C=C/C(N/5)=C(C6=CC=C(Br)C=C6)/C(C=C/7)=NC7=C(C8=CC=C(Br)C=C8)/C9=CC=C2N9)=N\3)C=C1	5,10,15,20-tetrakis(4-bromophenyl)-21,23-dihydroporphyrin	
linker99_C	BrC1=CC(C2=CC(Br)=CC(Br)=C2)=CC(Br)=C1	1,3-dibromo-5-(3,5-dibromophenyl)benzene	
linker100_C	BrC1=CC(C2=C(O)C(Br)=CC(Br)=C2)=C(O)C(Br)=C1	2,4-dibromo-6-(3,5-dibromo-2-hydroxyphenyl)phenol	

Table S4(continued): List of bromide linkers (Linker_C)

Linker Name	SMILES	PubChem IUPAC Name	Solution
linker101_C	BrC1=CC(C2=C(N)C(Br)=CC(Br)=C2)=C(N)C(Br)=C1	2-(2-amino-3,5-dibromophenyl)-4,6-dibromoaniline	
linker102_C	BrC1=CC(C2(C3=C(C=CC(Br)=C3)CC4=CC=C(Br)C=C42)C5=CC(Br)=CC=C5C6)=C6C=C1		Unsolved
linker103_C	BrC1=CC(C23C4=C(C5=C2C=C(Br)C=C5)C=CC(Br)=C4)=C(C6=C3C=C(Br)C=C6)C=C1	2,2',7,7'-tetrabromo-9,9'-spirobi[fluorene]	
linker104_C	OC1=C(Br)C=C(C2=C(O)C(Br)=C(O)C(Br)=C2)C(O)=C1Br	2,4-dibromo-6-(3,5-dibromo-2,4-dihydroxyphenyl)benzene-1,3-diol	
linker105_C	OC1=C(Br)C=C(C2=CC(Br)=C(O)C(Br)=C2)C=C1Br	2,6-dibromo-4-(3,5-dibromo-4-hydroxyphenyl)phenol	
linker106_C	NC1=C(Br)C=C(C2=C(N)C(Br)=C(N)C(Br)=C2)C(N)=C1Br		ASK
linker107_C	NC1=C(Br)C=C(C2=CC(Br)=C(N)C(Br)=C2)C=C1Br	4-(4-amino-3,5-dibromophenyl)-2,6-dibromoaniline	
linker108_C	C1=CC2=C3C(=C(C=C2Br)Br)C=CC4=C(C=C(C1=C43)Br)Br	1,3,6,8-tetrabromopyrene	
linker109_C	BrC1=CC=C(C(C2=CC=C(Br)C=C2)(C3=CC=C(Br)C=C3)C4=CC=C(Br)C=C4)C=C1	1-bromo-4-[tris(4-bromophenyl)methyl]benzene	
linker110_C	C1C2(CC3(CC1(CC(C2)(C3)Br)Br)Br)Br	1,3,5,7-tetrabromoadamantane	
linker111_C	BrC1=CC=C([Si](C2=CC=C(Br)C=C2)(C3=CC=C(Br)C=C3)C4=CC=C(Br)C=C4)C=C1	tetrakis(4-bromophenyl)silane	

Table S5: List of aldehyde linkers (Linker_CH, Linker_CH2, Linker_CO)

**Linker Name (X= CH,CH2,CO)**	**SMILES**	**PubChem IUPAC Name**	**Solution**
linker1_X	Nc1cc(C=O)ccc1C=O	2-aminoterephthalaldehyde	
linker2_X	O=Cc1ccc(C=O)c(O)c1	2-hydroxyterephthalaldehyde	
linker3_X	Cc1cc(C=O)ccc1C=O	2-methylterephthalaldehyde	
linker4_X	O=Cc1ccc(C=O)c([N+](=O)[O-])c1	2-nitroterephthalaldehyde	
linker5_X	Cc1cc(C=O)cc(C)c1C=O	2,6-dimethylterephthalaldehyde	
linker6_X	O=Cc1cc([N+](=O)[O-])c(C=O)c([N+](=O)[O-])c1		ASK+AZF
linker7_X	Cc1cc(C=O)c(C)cc1C=O	2,5-dimethylterephthalaldehyde	
linker8_X	O=Cc1cc([N+](=O)[O-])c(C=O)cc1[N+](=O)[O-]	2,5-dinitroterephthalaldehyde	
linker9_X	Cc1c(C=O)ccc(C=O)c1C	2,3-dimethylterephthalaldehyde	
linker10_X	O=Cc1ccc(C=O)c([N+](=O)[O-])c1[N+](=O)[O-]	2,3-dinitroterephthalaldehyde	
linker11_X	Nc1c(N)c(C=O)c(N)c(N)c1C=O		ASK
linker12_X	O=Cc1c(O)c(O)c(C=O)c(O)c1O		ASK+AZF
linker13_X	Cc1c(C)c(C=O)c(C)c(C)c1C=O	2,3,5,6-tetramethylterephthalaldehyde	
linker14_X	O=Cc1ccc(-c2ccc(C=O)cc2CO)c(CO)c1		ASK+AZF
linker15_X	Nc1cc(C=O)ccc1-c1ccc(C=O)cc1	3-amino-4-(4-formylphenyl)benzaldehyde	
linker16_X	Nc1cc(C=O)ccc1-c1ccc(C=O)cc1N		ASK+AZF
linker17_X	Nc1cc(-c2cc([N+](=O)[O-])c(C=O)cc2N)c([N+](=O)[O-])cc1C=O		Unsolved
linker18_X	Nc1cc(C=O)c([N+](=O)[O-])cc1-c1cc(N)c(C=O)c([N+](=O)[O-])c1		ASK
linker19_X	O=Cc1ccc(-c2ccc(C=O)cc2)cc1	4-(4-formylphenyl)benzaldehyde	
linker20_X	Cc1cc(C=O)ccc1-c1ccc(C=O)cc1C	4-(4-formyl-2-methylphenyl)-3-methylbenzaldehyde	
linker21_X	Cc1cc(C=O)cc(C)c1-c1c(C)cc(C=O)cc1C	4-(4-formyl-2,6-dimethylphenyl)-3,5-dimethylbenzaldehyde	
linker22_X	O=Cc1ccc(-c2ccc(C=O)cc2[N+](=O)[O-])cc1		ASK+AZF
linker23_X	O=Cc1ccc(-c2ccc(C=O)cc2[N+](=O)[O-])c([N+](=O)[O-])c1	4-(4-formyl-2-nitrophenyl)-3-nitrobenzaldehyde	

Table S5(continued): List of aldehyde linkers (Linker_CH, Linker_CH2, Linker_CO)

**Linker Name (X= CH,CH2,CO)**	**SMILES**	**PubChem IUPAC Name**	**Solution**
linker24_X	O=Cc1ccc(-c2c([N+](=O)[O-])cc(C=O)cc2[N+](=O)[O-])cc1		ASK+AZF
linker25_X	O=Cc1ccc(-c2ccc(C=O)c([N+](=O)[O-])c2)c([N+](=O)[O-])c1		ASK+AZF
linker26_X	O=Cc1cc([N+](=O)[O-])c(-c2c([N+](=O)[O-])cc(C=O)cc2[N+](=O)[O-])c([N+](=O)[O-])c1	4-(4-formyl-2,6-dinitrophenyl)-3,5-dinitrobenzaldehyde	
linker27_X	O=Cc1ccc2c(c1)CCc1cc(C=O)cc([N+](=O)[O-])c1-2		Unsolved
linker28_X	O=Cc1ccc(-c2ccc(C=O)c([N+](=O)[O-])c2)cc1		ASK+AZF
linker29_X	Nc1cc(C=O)c([N+](=O)[O-])cc1-c1cc([N+](=O)[O-])c(C=O)cc1N		Unsolved
linker30_X	O=Cc1ccc(-c2ccc(C=O)cn2)nc1	6-(5-formylpyridin-2-yl)pyridine-3-carbaldehyde	
linker31_X	Cc1cc(C=O)cnc1-c1ncc(C=O)cc1C		ASK
linker32_X	Nc1cc(C=O)cnc1-c1ncc(C=O)cc1N		ASK
linker33_X	Cc1nc2c(ccc3cc(C=O)c(C)nc32)cc1C=O		ASK
linker34_X	O=Cc1cnc(-c2ncc(C=O)cc2C(=O)O)c(C(=O)O)c1		ASK
linker35_X	O=Cc1cnc2c(ccc3cc(C=O)cnc32)c1	1,10-phenanthroline-3,8-dicarbaldehyde	
linker36_X	Cc1nc2c(ccc3cc(C=O)cnc32)cc1C=O		ASK+AZF
linker37_X	O=Cc1cnc(-c2ncc(C=O)cc2[N+](=O)[O-])c([N+](=O)[O-])c1		ASK
linker38_X	O=Cc1cnc2c(c1)c(O)c(O)c1cc(C=O)cnc12		Unsolved
linker39_X	O=Cc1cnc2c(c1)nnc1cc(C=O)cnc12		Unsolved
linker40_X	O=Cc1cnc2c(c1)C(=O)C(=O)c1cc(C=O)cnc1-2		ASK
linker41_X	O=Cc1ccc(C=O)c2ocnc12		ASK+AZF
linker42_X	O=Cc1c2ncoc2c(C=O)c2ncoc12		Unsolved
linker43_X	Cc1nc2c(C=O)c3oc(C)nc3c(C=O)c2o1		Unsolved
linker44_X	CCc1nc2c(C=O)c3oc(CC)nc3c(C=O)c2o1		Unsolved

Table S5(continued): List of aldehyde linkers (Linker_CH, Linker_CH2, Linker_CO)

Linker Name (X= CH,CH2,CO)	SMILES	PubChem IUPAC Name	Solution
linker45_X	O=Cc1ccc(C=O)c2nccnc12		ASK+AZF
linker46_X	Cc1cnc2c(C=O)ccc(C=O)c2n1		ASK+AZF
linker47_X	O=Cc1ccc(C=O)c2nc3ccccc3nc12		ASK+AZF
linker48_X	Cc1nc2c(C=O)ccc(C=O)c2nc1C		ASK+AZF
linker49_X	O=Cc1ccc(C=O)c2[nH]c(=O)cnc12		ASK+AZF
linker50_X	CNc1cnc2c(C=O)ccc(C=O)c2n1		AZF
linker51_X	O=Cc1ccc(C=O)c2nc3c(nc12)CCCC3		ASK
linker52_X	COc1cnc2c(C=O)ccc(C=O)c2n1		ASK+AZF
linker53_X	CCc1nc2c(C=O)ccc(C=O)c2nc1CC		ASK+AZF
linker54_X	O=Cc1ccc(C=O)c2[nH]c(=O)c(C(=O)O)nc 12		AZF
linker55_X	O=Cc1c2nccnc2c(C=O)c2nsnc12		Unsolved
linker56_X	Cc1nc2c(C=O)c3nsnc3c(C=O)c2nc1C		ASK
linker57_X	O=Cc1ccc(C=O)c2c(=O)nc[nH]c12		ASK+AZF
linker58_X	O=Cc1cnc(C=O)cn1	pyrazine-2,5-dicarbaldehyde	
linker59_X	Cc1nc(C=O)cnc1C=O		ASK+AZF
linker60_X	Cc1nc(C=O)c(C)nc1C=O	3,6-dimethylpyrazine-2,5-dicarbaldehyde	
linker61_X	Nc1nc(C=O)cnc1C=O		ASK+AZF
linker62_X	N#Cc1nc(C=O)cnc1C=O		ASK+AZF
linker63_X	O=Cc1cnc(C=O)c(CO)n1		ASK+AZF
linker64_X	O=Cc1cnc(C=O)c(C=O)n1	pyrazine-2,3,5-tricarbaldehyde	
linker65_X	N#Cc1nc(C=O)c(C#N)nc1C=O		ASK
linker66_X	COc1nc(C=O)cnc1C=O		ASK+AZF
linker67_X	O=Cc1cnc(C=O)c(C(=O)O)n1		ASK+AZF
linker68_X	O=Cc1cnc(C=O)c([N+](=O)[O-])n1		ASK
linker69_X	CC(=O)c1nc(C=O)cnc1C=O	3-acetylpyrazine-2,5-dicarbaldehyde	
linker70_X	COc1nc(C=O)c(OC)nc1C=O		ASK+AZF
linker71_X	NC(=O)c1nc(C=O)cnc1C=O		ASK+AZF
linker72_X	O=Cc1nc(C(=O)O)c(C=O)nc1C(=O)O		ASK
linker73_X	CN(C)/C=N/c1nc(C=O)cnc1C=O		ASK+AZF
linker74_X	COC(=O)c1nc(C=O)cnc1C=O		ASK+AZF
linker75_X	CCOC(=O)c1nc(C=O)cnc1C=O		ASK+AZF
linker76_X	O=Cc1cnc(C=O)nc1	pyrimidine-2,5-dicarbaldehyde	
linker77_X	Cc1nc(C=O)ncc1C=O		ASK+AZF

Table S5(continued): List of aldehyde linkers (Linker_CH, Linker_CH2, Linker_CO)

Linker Name (X= CH,CH2,CO)	SMILES	PubChem IUPAC Name	Solution
linker78_X	COc1nc(C=O)ncc1C=O		ASK+AZF
linker79_X	O=Cc1ncc(C=O)c(C2CC2)n1		ASK+AZF
linker80_X	CC(C)(C)c1nc(C=O)[nH]c(=O)c1C=O		ASK+AZF
linker81_X	O=Cc1ccc(C=O)nn1	pyridazine-3,6-dicarbaldehyde	
linker82_X	Cc1cc(C=O)nnc1C=O		ASK+AZF
linker83_X	O=Cc1nnc(C=O)c2ccccc12		ASK+AZF
linker84_X	Nc1cc(C=O)nnc1C=O		ASK
linker85_X	CC(=O)c1cc(C=O)nnc1C=O		ASK
linker86_X	O=Cc1nnc(C=O)c2sc(C(F)F)cc12		Unsolved
linker87_X	O=Cc1ccc(C=O)cc1	terephthalaldehyde	
linker88_X	O=Cc1cc(O)c(C=O)cc1O	2,5-dihydroxyterephthalaldehyde	
linker89_X	O=Cc1ccc2c(c1)CCc1cc(C=O)ccc1-2	9,10-dihydrophenanthrene-2,7-dicarbaldehyde	
linker90_X	O=Cc1c2cc3ccccc3cc2c(C=O)c2cc3ccccc3cc12	pentacene-6,13-dicarbaldehyde	
linker91_X	O=Cc1nc(C=O)nc(C=O)n1	1,3,5-triazine-2,4,6-tricarbaldehyde	
linker92_X	O=Cc1cc(C=O)cc(C=O)c1	benzene-1,3,5-tricarbaldehyde	
linker93_X	O=Cc1ccc(N(c2ccc(C=O)cc2)c2ccc(C=O)cc2)cc1	4-(4-formyl-N-(4-formylphenyl)anilino)benzaldehyde	
linker94_X	O=Cc1ccc(-c2cc(-c3ccc(C=O)cc3)cc(-c3ccc(C=O)cc3)c2)cc1	4-[3,5-bis(4-formylphenyl)phenyl]benzaldehyde	
linker95_X	O=Cc1ccc(/C=C/c2cc(/C=C/c3ccc(C=O)cc3)cc(/C=C/c3ccc(C=O)cc3)c2)cc1	4-[(E)-2-[3,5-bis[(E)-2-(4-formylphenyl)ethenyl]phenyl]ethenyl]benzaldehyde	
linker96_X	O=Cc1ccc(-c2nc(-c3ccc(C=O)cc3)nc(-c3ccc(C=O)cc3)n2)cc1	4-[4,6-bis(4-formylphenyl)-1,3,5-triazin-2-yl]benzaldehyde	
linker97_X	O=Cc1ccc(-c2ccc(-c3c4nc(c(-c5ccc(-c6ccc(C=O)cc6)cc5)c5ccc([nH]5)c(-c5ccc(-c6ccc(C=O)cc6)cc5)c5nc(c(-c6ccc(-c7ccc(C=O)cc7)cc6)c6ccc3[nH]6)C=C5)C=C4)cc2)cc1	4-[4-[10,15,20-tris[4-(4-formylphenyl)phenyl]-21,23-dihydroporphyrin-5-yl]phenyl]benzaldehyde	

Table S5(continued): List of aldehyde linkers (Linker_CH, Linker_CH2, Linker_CO)

**Linker Name (X= CH,CH2,CO)**	**SMILES**	**PubChem IUPAC Name**	**Solution**
linker98_X	O=Cc1ccc(-c2c3nc(c(-c4ccc(C=O)cc4)c4ccc([nH]4)c(-c4ccc(C=O)cc4)c4nc(c(-c5ccc(C=O)cc5)c5ccc2[nH]5)C=C4)C=C3)cc1	4-[10,15,20-tris(4-formylphenyl)-21,23-dihydroporphyrin-5-yl]benzaldehyde	
linker99_X	O=Cc1cc(C=O)cc(-c2cc(C=O)cc(C=O)c2)c1	5-(3,5-diformylphenyl)benzene-1,3-dicarbaldehyde	
linker100_X	O=Cc1cc(C=O)c(O)c(-c2cc(C=O)cc(C=O)c2O)c1		ASK
linker101_X	Nc1c(C=O)cc(C=O)cc1-c1cc(C=O)cc(C=O)c1N		ASK
linker102_X	O=Cc1ccc2c(c1)C1(c3cc(C=O)ccc3C2)c2cc(C=O)ccc2Cc2ccc(C=O)cc21		Unsolved
linker103_X	O=Cc1ccc2c(c1)C1(c3cc(C=O)ccc3-2)c2cc(C=O)ccc2-c2ccc(C=O)cc21	9,9'-spirobi[fluorene]-2,2',7,7'-tetracarbaldehyde	
linker104_X	O=Cc1cc(-c2cc(C=O)c(O)c(C=O)c2O)c(O)c(C=O)c1O		ASK
linker105_X	O=Cc1cc(-c2cc(C=O)c(O)c(C=O)c2)cc(C=O)c1O	5-(3,5-diformyl-4-hydroxyphenyl)-2-hydroxybenzene-1,3-dicarbaldehyde	
linker106_X	Nc1c(C=O)cc(-c2cc(C=O)c(N)c(C=O)c2N)c(N)c1C=O		Unsolved
linker107_X	Nc1c(C=O)cc(-c2cc(C=O)c(N)c(C=O)c2)cc1C=O		Unsolved
linker108_X	O=Cc1cc(C=O)c2ccc3c(C=O)cc(C=O)c4ccc1c2c43	pyrene-1,3,6,8-tetracarbaldehyde	
linker109_X	O=Cc1ccc(C(c2ccc(C=O)cc2)(c2ccc(C=O)cc2)c2ccc(C=O)cc2)cc1	4-[tris(4-formylphenyl)methyl]benzaldehyde	
linker110_X	O=CC12CC(CC(C3)(C=O)C2)(C=O)CC3(C=O)C1	adamantane-1,3,5,7-tetracarbaldehyde	
linker111_X	O=Cc1ccc([Si](c2ccc(C=O)cc2)(c2ccc(C=O)cc2)c2ccc(C=O)cc2)cc1	4-tris(4-formylphenyl)silylbenzaldehyde	

Table S6: List of aldehyde linkers (Linker_N, Linker_NH)

Linker Name (Y= N,NH)	SMILES	PubChem IUPAC Name	Solution
linker1_Y	Nc1ccc(N)c(N)c1	benzene-1,2,4-triamine	
linker2_Y	Nc1ccc(N)c(O)c1	2,5-diaminophenol	
linker3_Y	Cc1cc(N)ccc1N	2-methylbenzene-1,4-diamine	
linker4_Y	Nc1ccc(N)c([N+](=O)[O-])c1	2-nitrobenzene-1,4-diamine	
linker5_Y	Cc1cc(N)cc(C)c1N	2,6-dimethylbenzene-1,4-diamine	
linker6_Y	Nc1cc([N+](=O)[O-])c(N)c([N+](=O)[O-])c1	2,6-dinitrobenzene-1,4-diamine	
linker7_Y	Cc1cc(N)c(C)cc1N	2,5-dimethylbenzene-1,4-diamine	
linker8_Y	Nc1cc([N+](=O)[O-])c(N)cc1[N+](=O)[O-]	2,5-dinitrobenzene-1,4-diamine	
linker9_Y	Cc1c(N)ccc(N)c1C	2,3-dimethylbenzene-1,4-diamine	
linker10_Y	Nc1ccc(N)c([N+](=O)[O-])c1[N+](=O)[O-]	2,3-dinitrobenzene-1,4-diamine	
linker11_Y	Nc1c(N)c(N)c(N)c(N)c1N	benzene-1,2,3,4,5,6-hexamine	
linker12_Y	Nc1c(O)c(O)c(N)c(O)c1O	3,6-diaminobenzene-1,2,4,5-tetrol	
linker13_Y	Cc1c(C)c(N)c(C)c(C)c1N	2,3,5,6-tetramethylbenzene-1,4-diamine	
linker14_Y	Nc1ccc(-c2ccc(N)cc2CO)c(CO)c1	[5-amino-2-[4-amino-2-(hydroxymethyl)phenyl]phenyl]methanol	
linker15_Y	Nc1ccc(-c2ccc(N)cc2N)cc1	4-(4-aminophenyl)benzene-1,3-diamine	
linker16_Y	Nc1ccc(-c2ccc(N)cc2N)c(N)c1	4-(2,4-diaminophenyl)benzene-1,3-diamine	
linker17_Y	Nc1cc(-c2cc([N+](=O)[O-])c(N)cc2N)c([N+](=O)[O-])cc1N		ASK+AZF
linker18_Y	Nc1cc(N)c([N+](=O)[O-])cc1-c1cc(N)c(N)c([N+](=O)[O-])c1		ASK+AZF
linker19_Y	Nc1ccc(-c2ccc(N)cc2)cc1	4-(4-aminophenyl)aniline	
linker20_Y	Cc1cc(N)ccc1-c1ccc(N)cc1C	4-(4-amino-2-methylphenyl)-3-methylaniline	
linker21_Y	Cc1cc(N)cc(C)c1-c1c(C)cc(N)cc1C	4-(4-amino-2,6-dimethylphenyl)-3,5-dimethylaniline	
linker22_Y	Nc1ccc(-c2ccc(N)cc2[N+](=O)[O-])cc1	4-(4-aminophenyl)-3-nitroaniline	

Table S6(continued): List of aldehyde linkers (Linker_N, Linker_NH)

Linker Name (Y= N,NH)	SMILES	PubChem IUPAC Name	Solution
linker23_Y	Nc1ccc(-c2ccc(N)cc2[N+](=O)[O-])c([N+](=O)[O-])c1	4-(4-amino-2-nitrophenyl)-3-nitroaniline	
linker24_Y	Nc1ccc(-c2c([N+](=O)[O-])cc(N)cc2[N+](=O)[O-])cc1	4-(4-aminophenyl)-3,5-dinitroaniline	
linker25_Y	Nc1ccc(-c2ccc(N)c([N+](=O)[O-])c2)c([N+](=O)[O-])c1	4-(4-amino-2-nitrophenyl)-2-nitroaniline	
linker26_Y	Nc1cc([N+](=O)[O-])c(-c2c([N+](=O)[O-])cc(N)cc2[N+](=O)[O-])c([N+](=O)[O-])c1	4-(4-amino-2,6-dinitrophenyl)-3,5-dinitroaniline	
linker27_Y	Nc1ccc2c(c1)CCc1cc(N)cc([N+](=O)[O-])c1-2		Unsolved
linker28_Y	Nc1ccc(-c2ccc(N)c([N+](=O)[O-])c2)cc1	4-(4-aminophenyl)-2-nitroaniline	
linker29_Y	Nc1cc(N)c([N+](=O)[O-])cc1-c1cc([N+](=O)[O-])c(N)cc1N		ASK
linker30_Y	Nc1ccc(-c2ccc(N)cn2)nc1	6-(5-aminopyridin-2-yl)pyridin-3-amine	
linker31_Y	Cc1cc(N)cnc1-c1ncc(N)cc1C		ASK
linker32_Y	Nc1cnc(-c2ncc(N)cc2N)c(N)c1	2-(3,5-diaminopyridin-2-yl)pyridine-3,5-diamine	
linker33_Y	Cc1nc2c(ccc3cc(N)c(C)nc32)cc1N		ASK
linker34_Y	Nc1cnc(-c2ncc(N)cc2C(=O)O)c(C(=O)O)c1		ASK
linker35_Y	Nc1cnc2c(ccc3cc(N)cnc32)c1	1,10-phenanthroline-3,8-diamine	
linker36_Y	Cc1nc2c(ccc3cc(N)cnc32)cc1N		ASK
linker37_Y	Nc1cnc(-c2ncc(N)cc2[N+](=O)[O-])c([N+](=O)[O-])c1		ASK
linker38_Y	Nc1cnc2c(c1)c(O)c(O)c1cc(N)cnc12		Unsolved
linker39_Y	Nc1cnc2c(c1)nnc1cc(N)cnc12		Unsolved
linker40_Y	Nc1cnc2c(c1)C(=O)C(=O)c1cc(N)cnc1-2		ASK
linker41_Y	Nc1ccc(N)c2ocnc12	1,3-benzoxazole-4,7-diamine	
linker42_Y	Nc1c2ncoc2c(N)c2ncoc12		Unsolved
linker43_Y	Cc1nc2c(N)c3oc(C)nc3c(N)c2o1	2,6-dimethyl-[1,3]oxazolo[5,4-f][1,3]benzoxazole-4,8-diamine	

Table S6(continued): List of aldehyde linkers (Linker_N, Linker_NH)

Linker Name (Y= N,NH)	SMILES	PubChem IUPAC Name	Solution
linker44_Y	CCc1nc2c(N)c3oc(CC)nc3c(N)c2o1		Unsolved
linker45_Y	Nc1ccc(N)c2nccnc12	quinoxaline-5,8-diamine	
linker46_Y	Cc1cnc2c(N)ccc(N)c2n1		ASK+AZF
linker47_Y	Nc1ccc(N)c2nc3ccccc3nc12	phenazine-1,4-diamine	
linker48_Y	Cc1nc2c(N)ccc(N)c2nc1C		ASK+AZF
linker49_Y	Nc1ccc(N)c2[nH]c(=O)cnc12		ASK+AZF
linker50_Y	CNc1cnc2c(N)ccc(N)c2n1		ASK
linker51_Y	Nc1ccc(N)c2nc3c(nc12)CCCC3		ASK+AZF
linker52_Y	COc1cnc2c(N)ccc(N)c2n1		ASK
linker53_Y	CCc1nc2c(N)ccc(N)c2nc1CC		ASK
linker54_Y	Nc1ccc(N)c2[nH]c(=O)c(C(=O)O)nc12		Unsolved
linker55_Y	Nc1c2nccnc2c(N)c2nsnc12	[1,2,5]thiadiazolo[3,4-g]quinoxaline-4,9-diamine	
linker56_Y	Cc1nc2c(N)c3nsnc3c(N)c2nc1C		Unsolved
linker57_Y	Nc1ccc(N)c2c(=O)nc[nH]c12		ASK
linker58_Y	Nc1cnc(N)cn1	pyrazine-2,5-diamine	
linker59_Y	Cc1nc(N)cnc1N	3-methylpyrazine-2,5-diamine	
linker60_Y	Cc1nc(N)c(C)nc1N	3,6-dimethylpyrazine-2,5-diamine	
linker61_Y	Nc1cnc(N)c(N)n1	pyrazine-2,3,5-triamine	
linker62_Y	N#Cc1nc(N)cnc1N	3,6-diaminopyrazine-2-carbonitrile	
linker63_Y	Nc1cnc(N)c(CO)n1		ASK+AZF
linker64_Y	Nc1cnc(N)c(C=O)n1	3,6-diaminopyrazine-2-carbaldehyde	
linker65_Y	N#Cc1nc(N)c(C#N)nc1N	3,6-diaminopyrazine-2,5-dicarbonitrile	
linker66_Y	COc1nc(N)cnc1N	3-methoxypyrazine-2,5-diamine	
linker67_Y	Nc1cnc(N)c(C(=O)O)n1	3,6-diaminopyrazine-2-carboxylic acid	
linker68_Y	Nc1cnc(N)c([N+](=O)[O-])n1	3-nitropyrazine-2,5-diamine	
linker69_Y	CC(=O)c1nc(N)cnc1N		ASK+AZF
linker70_Y	COc1nc(N)c(OC)nc1N		ASK
linker71_Y	NC(=O)c1nc(N)cnc1N	3,6-diaminopyrazine-2-carboxamide	
linker72_Y	Nc1nc(C(=O)O)c(N)nc1C(=O)O	3,6-diaminopyrazine-2,5-dicarboxylic acid	
linker73_Y	CN(C)/C=N/c1nc(N)cnc1N		ASK+AZF

Table S6(continued): List of aldehyde linkers (Linker_N, Linker_NH)

Linker Name (Y= N,NH)	SMILES	PubChem IUPAC Name	Solution
linker74_Y	COC(=O)c1nc(N)cnc1N	methyl 3,6-diaminopyrazine-2-carboxylate	
linker75_Y	CCOC(=O)c1nc(N)cnc1N		ASK+AZF
linker76_Y	Nc1cnc(N)nc1	pyrimidine-2,5-diamine	
linker77_Y	Cc1nc(N)ncc1N	4-methylpyrimidine-2,5-diamine	
linker78_Y	COc1nc(N)ncc1N	4-methoxypyrimidine-2,5-diamine	
linker79_Y	Nc1ncc(N)c(C2CC2)n1	4-cyclopropylpyrimidine-2,5-diamine	
linker80_Y	CC(C)(C)c1nc(N)[nH]c(=O)c1N		ASK+AZF
linker81_Y	Nc1ccc(N)nn1	pyridazine-3,6-diamine	
linker82_Y	Cc1cc(N)nnc1N	4-methylpyridazine-3,6-diamine	
linker83_Y	Nc1nnc(N)c2ccccc12	phthalazine-1,4-diamine	
linker84_Y	Nc1cc(N)c(N)nn1	pyridazine-3,4,6-triamine	
linker85_Y	CC(=O)c1cc(N)nnc1N		AZF
linker86_Y	Nc1nnc(N)c2sc(C(F)F)cc12		Unsolved
linker87_Y	Nc1ccc(N)cc1	benzene-1,4-diamine	
linker88_Y	Nc1cc(O)c(N)cc1O	2,5-diaminobenzene-1,4-diol	
linker89_Y	Nc1ccc2c(c1)CCc1cc(N)ccc1-2	9,10-dihydrophenanthrene-2,7-diamine	
linker90_Y	Nc1c2cc3ccccc3cc2c(N)c2cc3ccccc3cc1 2	pentacene-6,13-diamine	
linker91_Y	Nc1nc(N)nc(N)n1	1,3,5-triazine-2,4,6-triamine	
linker92_Y	Nc1cc(N)cc(N)c1	benzene-1,3,5-triamine	
linker93_Y	Nc1ccc(N(c2ccc(N)cc2)c2ccc(N)cc2)cc 1	4-N,4-N-bis(4-aminophenyl)benzene-1,4-diamine	
linker94_Y	Nc1ccc(-c2cc(-c3ccc(N)cc3)cc(-c3ccc(N)cc3)c2)cc1	4-[3,5-bis(4-aminophenyl)phenyl]aniline	
linker95_Y	Nc1ccc(/C=C/c2cc(/C=C/c3ccc(N)cc3)c c(/C=C/c3ccc(N)cc3)c2)cc1	4-[(E)-2-[3,5-bis[(E)-2-(4-aminophenyl)ethenyl]phenyl]ethenyl] aniline	
linker96_Y	Nc1ccc(-c2nc(-c3ccc(N)cc3)nc(-c3ccc(N)cc3)n2)cc1	4-[4,6-bis(4-aminophenyl)-1,3,5-triazin-2-yl]aniline	

Table S6(continued): List of aldehyde linkers (Linker_N, Linker_NH)

Linker Name (Y= N,NH)	SMILES	PubChem IUPAC Name	Solution
linker97_Y	Nc1ccc(-c2ccc(-c3c4nc(c(-c5ccc(-c6ccc(N)cc6)cc5)c5ccc([nH]5)c(-c5ccc(-c6ccc(N)cc6)cc5)c5nc(c(-c6ccc(-c7ccc(N)cc7)cc6)c6ccc3[nH]6)C=C5)C=C4)cc2)cc1	4-[4-[10,15,20-tris[4-(4-aminophenyl)phenyl]-21,23-dihydroporphyrin-5-yl]phenyl]aniline	
linker98_Y	Nc1ccc(-c2c3nc(c(-c4ccc(N)cc4)c4ccc([nH]4)c(-c4ccc(N)cc4)c4nc(c(-c5ccc(N)cc5)c5ccc2[nH]5)C=C4)C=C3)cc1	4-[10,15,20-tris(4-aminophenyl)-21,23-dihydroporphyrin-5-yl]aniline	
linker99_Y	Nc1cc(N)cc(-c2cc(N)cc(N)c2)c1	5-(3,5-diaminophenyl)benzene-1,3-diamine	
linker100_Y	Nc1cc(N)c(O)c(-c2cc(N)cc(N)c2O)c1	2,4-diamino-6-(3,5-diamino-2-hydroxyphenyl)phenol	
linker101_Y	Nc1cc(N)c(N)c(-c2cc(N)cc(N)c2N)c1		ASK
linker102_Y	Nc1ccc2c(c1)C1(c3cc(N)ccc3C2)c2cc(N)ccc2Cc2ccc(N)cc21		Unsolved
linker103_Y	Nc1ccc2c(c1)C1(c3cc(N)ccc3-2)c2cc(N)ccc2-c2ccc(N)cc21	9,9'-spirobi[fluorene]-2,2',7,7'-tetramine	
linker104_Y	Nc1cc(-c2cc(N)c(O)c(N)c2O)c(O)c(N)c1O		Unsolved
linker105_Y	Nc1cc(-c2cc(N)c(O)c(N)c2)cc(N)c1O	2,6-diamino-4-(3,5-diamino-4-hydroxyphenyl)phenol	
linker106_Y	Nc1cc(-c2cc(N)c(N)c(N)c2N)c(N)c(N)c1N		Unsolved
linker107_Y	Nc1cc(-c2cc(N)c(N)c(N)c2)cc(N)c1N	5-(3,4,5-triaminophenyl)benzene-1,2,3-triamine	
linker108_Y	Nc1cc(N)c2ccc3c(N)cc(N)c4ccc1c2c43	pyrene-1,3,6,8-tetramine	
linker109_Y	Nc1ccc(C(c2ccc(N)cc2)(c2ccc(N)cc2)c2ccc(N)cc2)cc1	4-[tris(4-aminophenyl)methyl]aniline	
linker110_Y	NC(C1)(C2)CC3(N)CC2(N)CC1(N)C3	adamantane-1,3,5,7-tetramine	
linker111_Y	Nc1ccc([Si](c2ccc(N)cc2)(c2ccc(N)cc2)c2ccc(N)cc2)cc1	4-tris(4-aminophenyl)silylaniline	

References

[1] A Bettelheim, BA White, SA Raybuck, and Royce W Murray. Electrochemical polymerization of amino-, pyrrole-, and hydroxy-substituted tetraphenylporphyrins. *Inorganic Chemistry*, 26(7):1009–1017, 1987.

[2] Ricardo JGB Campello, Davoud Moulavi, and Jörg Sander. Density-based clustering based on hierarchical density estimates. In *Pacific-Asia conference on knowledge discovery and data mining*, pages 160–172. Springer, 2013.

[3] Samuel Genheden, Amol Thakkar, Veronika Chadimová, Jean-Louis Reymond, Ola Engkvist, and Esben Bjerrum. Aizynthfinder: a fast, robust and flexible open-source software for retrosynthetic planning. *Journal of cheminformatics*, 12(1):70, 2020.

[4] Suk Joong Lee and Wenbin Lin. A chiral molecular square with metallo-corners for enantioselective sensing. *Journal of the American Chemical Society*, 124(17):4554–4555, 2002.

[5] Leland McInnes, John Healy, and James Melville. Umap: uniform manifold approximation and projection for dimension reduction. arxiv. *arXiv preprint arXiv:1802.03426*, 10, 2018.

[6] Rocío Mercado, Rueih-Sheng Fu, Aliaksandr V. Yakutovich, Leopold Talirz, Maciej Haranczyk, and Berend Smit. In silico design of 2d and 3d covalent organic frameworks for methane storage applications. *Chemistry of Materials*, 30(15):5069–5086, 2018.

[7] Adrian Mirza, Nawaf Alampara, Sreekanth Kunchapu, Martiño Ríos-García, Benedict Emoekabu, Aswanth Krishnan, Tanya Gupta, Mara Schilling-Wilhelmi, Macjonathan Okereke, Anagha Aneesh, et al. A framework for evaluating the chemical knowledge and reasoning abilities of large language models against the expertise of chemists. *Nature Chemistry*, 17(7):1027–1034, 2025.

[8] Perumal Rajakumar, Merikapudi Gayatri Swaroop, S Jayavelu, and K Murugesan. Synthesis, complexation studies and biological applications of some novel stilbenophanes, indolophanes and bisindolostilbenophanes via mcmurry coupling. *Tetrahedron*, 62(51):12041–12050, 2006.

[9] Fernando J Uribe-Romo, Christian J Doonan, Hiroyasu Furukawa, Kounosuke Oisaki, and Omar M Yaghi. Crystalline covalent organic frameworks with hydrazone linkages. *Journal of the American Chemical Society*, 133(30):11478–11481, 2011.

[10] Cory Valente, Eunwoo Choi, Matthew E Belowich, Christian J Doonan, Qiaowei Li, Travis B Gasa, Youssry Y Botros, Omar M Yaghi, and J Fraser Stoddart. Metal–organic frameworks with designed chiral recognition sites. *Chemical communications*, 46(27):4911–4913, 2010.

[11] Xiaowei Wu, Xing Han, Qisong Xu, Yuhao Liu, Chen Yuan, Shi Yang, Yan Liu, Jianwen Jiang, and Yong Cui. Chiral binol-based covalent organic frameworks for enantioselective sensing. *Journal of the American Chemical Society*, 141(17):7081–7089, 2019.
